# Efficacy and safety of ketamine and esketamine for unipolar and bipolar depression: an overview of systematic reviews with meta-analysis

**DOI:** 10.3389/fpsyt.2024.1325399

**Published:** 2024-02-01

**Authors:** Alessandro Rodolico, Pierfelice Cutrufelli, Antonio Di Francesco, Andrea Aguglia, Gaetano Catania, Carmen Concerto, Alessandro Cuomo, Andrea Fagiolini, Giuseppe Lanza, Ludovico Mineo, Antimo Natale, Laura Rapisarda, Antonino Petralia, Maria Salvina Signorelli, Eugenio Aguglia

**Affiliations:** ^1^Department of Clinical and Experimental Medicine, Institute of Psychiatry, University of Catania, Catania, Italy; ^2^Department of Neuroscience, Rehabilitation, Ophthalmology, Genetics, Maternal and Child Health, Section of Psychiatry, University of Genoa, Genoa, Italy; ^3^IRCCS Ospedale Policlinico San Martino, Genoa, Italy; ^4^University of Catania, Catania, Italy; ^5^Department of Molecular Medicine, University of Siena, Siena, Italy; ^6^Department of Surgery and Medical-Surgical Specialties, University of Catania, Catania, Italy; ^7^Clinical Neurophysiology Research Unit, Oasi Research Institute-IRCCS, Troina, Italy; ^8^Department of Psychiatry, Adult Psychiatry Service (SPA), University Hospitals of Geneva (HUG), Geneva, Switzerland; ^9^Department of Biomedical and Biotechnological Sciences, Section of Pharmacology, University of Catania, Catania, Italy

**Keywords:** unipolar depression, bipolar depression, ketamine, esketamine, suicidal ideation, treatment resistance, Overview of Reviews

## Abstract

**Background:**

Unipolar and bipolar depression present treatment challenges, with patients sometimes showing limited or no response to standard medications. Ketamine and its enantiomer, esketamine, offer promising alternative treatments that can quickly relieve suicidal thoughts. This Overview of Reviews (OoR) analyzed and synthesized systematic reviews (SRs) with meta-analysis on randomized clinical trials (RCTs) involving ketamine in various formulations (intravenous, intramuscular, intranasal, subcutaneous) for patients with unipolar or bipolar depression. We evaluated the efficacy and safety of ketamine and esketamine in treating major depressive episodes across various forms, including unipolar, bipolar, treatment-resistant, and non-resistant depression, in patient populations with and without suicidal ideation, aiming to comprehensively assess their therapeutic potential and safety profile.

**Methods:**

Following PRIOR guidelines, this OoR’s protocol was registered on Implasy (ID:202150049). Searches in PubMed, Scopus, Cochrane Library, and Epistemonikos focused on English-language meta-analyses of RCTs of ketamine or esketamine, as monotherapy or add-on, evaluating outcomes like suicide risk, depressive symptoms, relapse, response rates, and side effects. We included studies involving both suicidal and non-suicidal patients; all routes and formulations of administration (intravenous, intramuscular, intranasal) were considered, as well as all available comparisons with control interventions. We excluded meta-analysis in which the intervention was used as anesthesia for electroconvulsive therapy or with a randomized ascending dose design. The selection, data extraction, and quality assessment of studies were carried out by pairs of reviewers in a blinded manner. Data on efficacy, acceptability, and tolerability were extracted.

**Results:**

Our analysis included 26 SRs and 44 RCTs, with 3,316 subjects. The intervention is effective and well-tolerated, although the quality of the included SRs and original studies is poor, resulting in low certainty of evidence.

**Limitations:**

This study is limited by poor-quality SRs and original studies, resulting in low certainty of the evidence. Additionally, insufficient available data prevents differentiation between the effects of ketamine and esketamine in unipolar and bipolar depression.

**Conclusion:**

While ketamine and esketamine show promising therapeutic potential, the current evidence suffers from low study quality. Enhanced methodological rigor in future research will allow for a more informed application of these interventions within the treatment guidelines for unipolar and bipolar depression.

**Systematic review registration:**

[https://inplasy.com/inplasy-2021-5-0049/], identifier (INPLASY202150049).

## 1 Introduction

Major Depressive Disorder (MDD) is a psychiatric condition with a prevalence of 4.4% worldwide (1). The text revision of the fifth edition of the Diagnostic and Statistical Manual of Mental Disorders (DSM-5-TR) defines the MDD as a minimum of 2 weeks of low mood or loss of interest in daily activities, accompanied by vegetative, motor, and cognitive symptoms. Depressed individuals may also have suicidal thoughts or tendencies (2). Bipolar disorder (BD) is characterized by alternating depressive and (hypo)manic episodes. In accordance with the DSM-5-TR, while the depressive phase of BD shares the same criteria as MDD, the manic and hypomanic phases are characterized by an elevation in mood, increased psychomotor activity, inflated self-esteem, risky behaviors, and reduced need for sleep. In more severe cases (mania), psychotic or more severe symptoms may also be present, leading to a decline in functioning or necessitating hospitalization (2). BD affects approximately 40 million individuals in the general population and has a significant impact on an individual’s quality of life, relationships, and occupational functioning (3).

The pathogenesis of MDD in both unipolar and bipolar depression is very complex and still partly unknown, due to the interaction between both genetic and environmental factors (4). The monoaminergic hypothesis, which postulates deficits in neurotransmission as the cause of depression, has historically been considered to explain depressive pathophysiology. In particular, dysfunctions in norepinephrine, serotonin, and dopamine neurotransmissions are implicated in the disorder (5). Treatment with antidepressants that increase serotonin levels alone is not recommended for BD, as it exposes the patient to the risk of a (hypo)manic switch. The preferred treatment involves the use of mood stabilizers, such as lithium or antiepileptic drugs, which exert their effect by stabilizing neurotransmission, and second-generation antipsychotics with a specific antagonistic action on the 5-HT_2A_ receptor (6). This antagonism would lead to an increase in the release of serotonin in the synaptic cleft, combined with the blockade of dopamine receptors to prevent potential bipolar switches (7). In general, the monoaminergic hypothesis does not provide a full understanding of neurochemistry of major depressive episode and alterations in γ-amino-butyric acid (GABA), glutamatergic and opioid endogenous neurotransmission may be also implied (8). As a result, multiple medications have been developed with varying degrees of specificity toward these neurotransmitter systems.

The most prescribed antidepressant drugs are selective serotonin reuptake inhibitors (SSRIs) with a more favorable balance between effectiveness and tolerability (9). The basic mechanism of action of SSRIs involves inhibition of the reuptake of serotonin released by neurons. Other antidepressant drugs also promote noradrenergic (norepinephrine and serotonine reuptake inhibitors, SNRIs) and dopaminergic (norepinephrine and dopamine reuptake inhibitors, NDRIs, i.e., bupropion) neurotransmission (5). On the other hand, the management of BD involves a combination of pharmacotherapy, psychotherapy, and lifestyle modifications (10), as well as several non-pharmacological approaches (11). While there are also other molecules with antidepressant action, which altogether would theoretically allow even more specific intervention toward individual depressive symptoms (12), still many patients achieve partial response or become resistant to treatment (13, 14). The Food and Drug Administration (FDA) and the European Medicines Agency (EMA) defines the treatment resistant depression (TRD) as a non-response to ≥2 antidepressant trials prescribed with adequate dose and duration (≥ 6 weeks) (15, 16). TRD can be also treated with the augmentation strategies as a second-generation antipsychotic or lithium (17).

In recent years, ketamine and its levogyre enantiomer, esketamine, have demonstrated a promising rapid antidepressant and anti-suicidal effect, particularly in individuals resistant to other medications (18). They were also remarkable for their status as the first antidepressants purportedly able to alleviate depression and, notably, suicidal ideation within hours for many patients (19). Intravenously administered ketamine is a racemic mixture of the R and S enantiomers, both of which have overlapping actions on the glutamatergic N-methyl-D-aspartic acid (NMDA) receptor contributing to its antidepressant action as well as on the σ1 receptor (20). Recently, the use of intranasally administered levogyre enantiomer of ketamine (i.e., esketamine) has been approved in TRD (21), resulting also a favorable alternative pharmacological approach for BD, especially in those cases resistant to traditional medications (22). Moreover, intranasal administration route has made clinical management more convenient by eliminating the need for intravenous infusion sessions. Specifically, the intranasal spray can be administered on a weekly or biweekly basis after an initial phase of twice-weekly administration (23).

Several clinical studies were conducted to test efficacy and tolerability of ketamine and derivatives in unipolar and bipolar depression (24). Consequently, a plethora of meta-analyses have been produced to synthesize the available data. Writing systematic reviews with meta-analysis involves the application of standard criteria (25), which are not always met (26). However, this is significant, both for clinicians and researchers, because, when available, guidelines that inform clinical practice rely heavily on meta-analyses (27). The study design suitable for synthesizing multiple systematic reviews is the Overview of Reviews (OoR) (28). In 2021, de Mendonça Lima and collaborators produced an OoR on the efficacy and tolerability of ketamine in the treatment of depression (29), whereas Shamabadi and colleagues produced an OoR on ketamine effect on suicidality (30). Given the number of new systematic reviews with meta-analysis to date produced, the aim of this study is to consolidate the rapidly growing body of literature on the efficacy and safety of ketamine and esketamine on unipolar and bipolar depression using standard criteria (31). By offering a comprehensive and cohesive overview of the existing evidence, this study is aimed to support evidence-based decision-making for clinicians, researchers, and policymakers in the field.

## 2 Materials and methods

### 2.1 Eligibility criteria

Only systematic reviews containing at least one meta-analysis on randomized clinical trials, which were either cluster type (where groups of individuals are randomized) or non-cluster (where individuals are randomized) have been included. Only English-language studies, published in indexed journals, without any restriction on publication date were retained. To be eligible for inclusion, meta-analyses had to analyze original studies involving human patients with unipolar, bipolar, resistant, or non-resistant major depressive episode, regardless of the diagnostic criteria used. We included studies involving both suicidal and non-suicidal patients. The study must have focused on the use of ketamine or its levogyre enantiomer (esketamine) as a treatment, administered via any route and formulation (either intravenous, intramuscular, intranasal, or subcutaneous), either as monotherapy or in combination with other drugs. The study must have included a comparator treatment, such as another antidepressant agent, an active or inactive placebo; finally, the included reviews had to contain at least one of the following outcomes: suicide risk, depressive symptomatology, relapse rate, treatment response rate, dropout rate, dissociative or psychotic symptomatology as side effects.

We excluded meta-analyses that included original studies investigating the effect of ketamine as an anesthetic treatment before electroconvulsive therapy, as well as studies with a randomized ascending dose design that did not report data separately for each time-point. The latter category of studies is designed to determine the optimal dose for efficacy and safety and often interrupts the control treatment during the trial. To include only those meta-analyses that met these inclusion/exclusion criteria, we read and extracted the original studies included in the individual meta-analyses, but we did not include or analyze any study not covered in the included systematic reviews. We followed the definition of systematic review proposed by the Cochrane Handbook, i.e., studies that are designed to “collate evidence that fits pre-specified eligibility criteria in order to answer a specific research question” (32).

### 2.2 Information sources and search strategy

The study search is updated to December 31, 2022. We searched two bibliographic databases (Scopus and MEDLINE via PubMed) and two systematic review databases (Cochrane Database of Systematic Reviews [CDSR] and Epistemonikos). We checked the references of the included systematic reviews, including any that did not appear in the search. We used the following search string: (‘ketamine’ OR ‘n-methylketamine’ OR ‘s-ketamine’ OR ((‘n-methylaspartate’ OR ‘nmda’) AND antagonist)). We used the official PubMed filter for systematic reviews and meta-analyses (systematic[sb]) (33) and adapted it to limit the search to reviews in Scopus. In Epistemonikos, the results were filtered by systematic reviews and in CDSR, only systematic reviews were considered.

### 2.3 Selection and data collection process

The Rayyan website was used for the title/abstract screening process. This website allows for semi-automatic deduplication of studies. Authors (PC, ADF, LR, AN) screened in pairs the studies to be included by checking their title and abstract. The same authors, again in pairs, selected the potentially candidate studies by checking their full text by using Airtable relational database. At each step, whenever disagreement emerged among the authors, a third author (AR) resolved it. The whole process was blinded, except in cases of disagreement. All reviews that met the predefined criteria were included, regardless of the degree of overlap in the populations involved or the interventions compared. Furthermore, systematic reviews with identical inclusion criteria were also retained.

To provide an overview of the overlap between different systematic reviews, we created multiple citation matrices categorized by the diagnosis of the patients included. These matrices indicated not only the presence of the study in the specific meta-analysis, but also the outcomes for which it had been considered. The authors (PC, ADF, LR, AN, GC) extracted the data contained in the studies independently and in a blind manner. The procedure was done using the relational database (Airtable) that automatically identified if there was disagreement in the extracted data, so that a final unique database was generated.

### 2.4 Data items

For each systematic review, we extracted the following study variables: search engines used, date of last search, inclusion and exclusion criteria of individual reviews, potential authors’ conflict of interest, project funding, diagnosis of included patients, drug(s) investigated, dose of interventional drug, and comparator(s). In addition, the following outcomes were extracted: response (as defined by the authors), remission (as defined by the authors), depressive symptoms, total dropouts, suicidality risk scales, all available adverse events (e.g., dissociative, psychotic, gastroenteric, neurological, etc.).

Regardless of the time points suggested in the individual meta-analyses, we grouped the time points as follows: ≤60 min, 61–90 min, 91–120 min, 121–240 min, 24–48 h, 3–6 days, 7–13 days, 14–28 days, >28 days. Time points that did not fall into these categories were adjusted. Endpoint data were collected. For each meta-analysis, when possible, statistical model adopted, type of effect size and its measure, with respective low and high confidence intervals, *p* value of statistical significance of comparisons, heterogeneity of the meta-analysis, the test used to measure it, and the statistical significance of the test were collected.

### 2.5 Quality assessment of the systematic reviews

The methodological quality of the included systematic reviews was assessed using the AMSTAR-2 (34). It is a widely used tool for conducting rapid, reliable, and reproducible critical quality assessment of RCT reviews on the effectiveness of health care interventions. The tool assesses the presence of any critical issues, distinguishing them into minor and major, thereby identifying the reliability of the review. A systematic review is considered having a high reliability if no more than one minor criticality is present, moderate if more than one minor criticality is present, low in the presence of at least one major criticality, and very low if multiple major critical elements are present. Each author used this tool independently and separately, blindly from each other. Reviewers in couples evaluated all studies. After blinding was broken, a final decision on AMSTAR-2 scoring was reached through discussion. If necessary, a third author (AR) was involved. Due to the absence of a specific tool to apprise the quality network meta-analyses, we adapted the AMSTAR-2 for this scope.

### 2.6 Confidence in results assessment

We took the Grading of Recommendations, Assessment, Development and Evaluations (GRADE) scores (35) of the systematic reviews whenever reported. GRADE is a widely used system for grading the quality of evidence of systematic reviews and meta-analyses to pose clinical recommendations. There are four distinct levels of evidence according to this framework, which can be very low, low, moderate, or high. These four levels of certainty correspond to the progressively increasing degree of fit between the estimated effect and the true effect. The scoring process considers the assessment of the risk of bias of the included studies, the degree of imprecision of the effect estimate, the degree of inconsistency among studies, the degree of correspondence between the measure being investigated and the instruments used to measure it (indirectness), and the impact of missing evidence (publication bias).

### 2.7 Risk of bias and reporting bias assessment

Where reported in the various systematic reviews, the risk of bias of the individual original studies was extracted. We’ve also synthesized the risk of bias to allow for a comparison of outcomes between the original and the 2.0 version of the tool, as well as between ketamine and esketamine. If present, the reporting bias, the statistical tool used to measure it and its statistical significance were also extracted from the reviews.

### 2.8 Synthesis methods

The data was summarized in descriptive tables, which were grouped by outcome and distinguished by the type of depression studied, including unipolar or bipolar depression, and TRD or non-TRD. In addition, data were summarized in a narrative manner. In the summary, the data presented do not distinguish between ketamine and its racemic formulation. However, where noteworthy differences arose, these were explicitly stated. In the extraction process, all sensitivity and subgroup analysis relevant to the clinical question of this paper (unipolar vs. bipolar; resistant vs. non-resistant; current suicidal ideation present vs. no suicidal ideation) were extracted separately and tabulated. Any discrepancy between systematic reviews was reported.

## 3 Results

### 3.1 Study selection

As reported in the PRISMA flowchart (Supplementary Figure 1), the search produced a total of 2,256 studies, reduced to 1,770 after the deduplication process. Thus, through the title/abstract screening process, 1,715 records were excluded. The full texts of the remaining 55 studies were viewed and 29 studies were excluded, which are shown in the Supplementary Table 1. Thirty-one reviews with meta-analysis were considered, of those two were updates of previous meta-analysis by the same group of authors (36, 37) and one had been retracted (38), thus the final number of individual independent reviews corresponded to 26 (Supplementary Table 1) and 44 RCTs (reported in the Supplementary Table 2) with a total of 3,316 subjects. Among the included studies, there were two network meta-analyses involving ketamine as intervention, one about all available medications for acute bipolar depression (36), and the other on TRD drugs (39). We excluded meta-analyses that contained original studies from the systematic reviews that did not meet the inclusion criteria for this overview. The specific individual original studies that were excluded are listed in the Supplementary Table 3.

### 3.2 Characteristics of included studies

Supplementary Table 1 shows the characteristics of the included systematic reviews. Most of the reviews used MEDLINE as a scientific search engine. Other commonly used engines were Embase and PsycINFO. The most recent scientific databases search of the included reviews was dated December 1, 2021. As per the inclusion criteria, all studies were on parallel or crossover RCTs. Most of the included studies indistinctly involved patients with unipolar and bipolar depression (40–50), with some exceptions, where only patients with unipolar (39, 51–56) or bipolar depression (36, 57, 58) were included. Only four reviews (39, 58–60) involved patients who had previously shown resistance to antidepressant treatment by inclusion criterion. Fifteen reviews considered any route of administration of the intervention (39, 41, 42, 44, 46, 47, 49, 50, 54, 55, 57–59, 61, 62). Three reviews considered only intravenous ketamine administration (36, 45, 60), whereas some others considered also the intranasal use (40, 41, 46, 51–53, 56, 63, 64). One of the included reviews considered only oral ketamine use (48). The majority of the reviews included in this OoR incorporated studies that used saline solution as the comparator for ketamine and esketamine (40, 45–47, 50, 51, 54, 56, 59, 63). Conversely, in other reviews, alternative comparators such as midazolam, diclofenac, and electroconvulsive therapy (ECT), were also included.

Regarding funding sources, nine studies reported public funding (41–45, 51, 56, 57, 62), one study reported private funding (39), and one study reported combined public and private fundings (55). Nine studies reported no funding (36, 40, 46, 52, 54, 58, 60, 63, 64), whereas information about funding was not available for six studies (47–50, 53, 59). In sixteen studies the authors reported conflict of interest (39–42, 45–49, 52, 53, 55, 57, 58, 63, 64). In eight studies the authors explicitly denied any conflicts of interest (44, 50, 51, 54, 56, 59, 60, 62). In one study, information about conflicts of interest was not reported (43).

### 3.3 Primary studies overlap

The citation matrices (Supplementary File 4) display the included studies and the outcomes analyzed in each meta-analysis. The most frequently included studies in the meta-analyses were Diazgranados et al. (65), Murrough et al. (66), Sos et al. (67), Zarate et al. (68), and Zarate et al. (69). The inclusion of the other studies was less consistent, across the various meta-analyses.

### 3.4 Risk of bias of included studies

AMSTAR-2 was applied on all systematic reviews. Most of the studies (23 of 26) had critically low quality. The remaining three studies had low quality (42, 57, 60). The scoring is given in more detail in Supplementary Table 2. Out of the 26 studies that were analyzed for quality scoring, only 5 of them (42, 48, 57, 58, 62) had a written protocol in advance. Additionally, only 6 studies (42, 46, 51, 57, 58, 60) included the list of the excluded studies, while 11 out of 26 studies argued in the discussion about the risk of bias of the included studies (36, 41–43, 46, 53, 54, 57, 59, 62, 70). In half of the studies (13 out of 26) (36, 42–45, 48, 53, 56–58, 60, 62, 63) a comprehensive literature search was performed and, in 15 out of 26 studies (36, 40, 41, 43–46, 49, 50, 53, 56, 59, 60, 63, 64), the authors explored how publication bias affected the outcomes of their meta-analysis. Although it is considered a minor issue in the scoring of AMSTAR-2, it should be noted that all but one (45) of the studies did not report data on the funding of the original studies included in the reviews.

### 3.5 Summary of results

Supplementary Table 3 provides a depiction of the meta-analyses, categorized by diagnosis and time points. A comprehensive report of the meta-analyses can be found in the Supplementary Table 5.

#### 3.5.1 Depressive symptoms

The intervention group shows greater reduction in depressive symptoms compared to the control group at all time points, up to 3–6 days. However, for patients with BD, there is no difference between the intervention and the comparator from 7 to 13-day time point. The lack of efficacy for BD primarily stems from meta-analyses on ketamine, not esketamine. For patients with MDD, the intervention’s efficacy persists in most of the analyses at later time points.

#### 3.5.2 Remission rate

Despite the absence of differences in the remission rate between the intervention and comparator groups at the 60-min time-point, the intervention arm generally displayed superiority over the control group in subsequent time-points, up until 3–6 days. Notably, the effectiveness of ketamine at the 24–48 h time-point revealed inconsistency, with half of the studies indicating no efficacy, irrespective of diagnosis and comparator. In the time-points exceeding 3–6 days, the differences in patients with MDD were not always consistent, with some meta-analyses showing the experimental arm superior to the control, while others did not. Conversely, no superiority of the intervention over control was observed in meta-analyses solely involving patients with BD. Even though results beyond 3–6 days generally did not favor the intervention, all meta-analyses on esketamine, which exclusively involved patients with unipolar depression, suggested a greater efficacy compared to the control arm.

#### 3.5.3 Response rate

Regarding the response rate, the intervention proved to be superior to the control arm for all time points, from <60 min to the 24–48-h range, except for one meta-analysis (57). Subsequently, analyses involving patients with unipolar depression demonstrated a substantial superiority of the intervention arm over control, except for a few meta-analyses, while those involving only patients with BD did not show any difference. It’s important to note that all available data on esketamine involve only patients with unipolar depression and consistently suggest greater efficacy in respect to the comparator. On the other hand, data on ketamine, involving both unipolar and bipolar depression patients, present less homogeneous results.

#### 3.5.4 Suicide scales

The suicide scales did not show any difference between the intervention and control groups at less than 60 min time point. There were no data available for the time points of 60–90 and 90–120 min. Meta-analyses showed that the intervention was more effective than the placebo from the time point of 120–240 min to 3–6 days. Only one meta-analysis, including patients with BD has been conducted (57); evaluating the outcome at the 24–48-h time point no difference between the two groups was found. While the available data for esketamine are consistent, favoring the intervention over the control, it is not the case for some time-points for ketamine, where the data for this outcome are scarce. Moreover, no data are available for esketamine beyond the 24–48 h.

#### 3.5.5 Dropout rates

Both the intervention, including both ketamine and esketamine, and control groups had similar dropout rates in all meta-analyses. This data was provided at >28 day time-point and at endpoints.

#### 3.5.6 Tolerability (adverse effects)

Ten reviews have thoroughly investigated the tolerability of treatment (41, 42, 45–48, 50, 51, 53, 56). Dissociative symptoms were investigated in three reviews (45–47) by using Clinician-Administered Dissociative States Scale (CADSS), revealing no notable discrepancies between intervention and control groups, aside from the results at the <60-min time-point, where the intervention group demonstrated higher scores. There is no data available for CADSS solely on esketamine, while data is available from meta-analyses solely on ketamine and from mixed meta-analyses. On the other hand, four reviews (41, 42, 53, 56) assessed the presence or absence of dissociation, challenging CADSS data and indicating an elevated occurrence of dissociative events at the 14–28 day and > 28-day periods. The only available data for ketamine, coming from a small number of patients, suggests no difference between ketamine and saline solution at the endpoint. A different result is found for esketamine, where dissociative symptoms persist even in the long term.

No differences were found between patients receiving the intervention or comparator for most of the other side effects, except for blurred vision, confusion, diplopia, dizziness, dysgeusia, emotional blunting, feeling abnormal, feeling drunk, hypoesthesia, headache, oral hypoesthesia, increased blood pressure, lethargy, paresthesia, postural dizziness, sedation, somnolence, throat irritation, vertigo, nausea, and vomiting. There were no obvious differences between the side effects for the different formulations, apart from a few exceptions. Dizziness did not vary between ketamine and the control at 7–13 days. Headache was typically the same for both groups, though one study found it to be slightly more common after 28 days with esketamine. Lastly, esketamine resulted in more nausea and vomiting compared to control, a trend not observed with ketamine.

#### 3.5.7 Data heterogeneity

Overall, heterogeneity data were reported unsystematically. Often statistical tests excluded its presence in meta-analyses. The only outcomes showing some statistical heterogeneity were depressive symptoms (36, 40, 43, 51, 54), response (36, 51, 56, 58), suicide scales (63), BPRS (50), and CADSS (45).

### 3.6 Reporting biases

A very small number of systematic reviews reported the presence of publication bias which, in most cases, was visually investigated with funnel plots. Moreover, those were often used non-canonically, as they included fewer than 10 original studies (71). In any case, of the few studies reporting the information, the data were discordant and inconclusive for most outcomes.

### 3.7 Risk of bias of original studies and outcomes certainty of evidence

#### 3.7.1 Risk of bias of original studies

A complete report of the risk of bias of the included studies is detailed in the Supplementary Table 6. Study quality was measured in most of the included reviews. Four studies did not perform any Risk of Bias measurement (44, 47, 50, 52). The most used tool was the Cochrane’s Risk of Bias in its original version, while Risk of Bias 2.0 was used in four recent reviews (40, 49, 53, 63). In addition, the Jadad score (72) and the Downs and Black checklist (73) have only been used in three systematic reviews (56, 60, 64).

From the 16 systematic reviews that used the original Risk of Bias tool, it emerges that most studies performed randomization adequately. However, in several reviews, authors noted that there was a high risk of bias in the included studies for failure to allocate concealment and inadequate blinding of recruiting staff and assessors’ blinding domains. Additionally, original studies suffered from incomplete outcome reporting and selective reporting. For the Risk of Bias 2.0 domains, there was generally a satisfactory randomization process, although some studies exhibited a higher risk of bias due to possible deviations from the intervention and incomplete data reporting. Nevertheless, outcomes were overall adequately measured and there was no data selection bias detected. Regarding the presence of other biases in the studies, many reviews found a high risk of bias, but this category encompasses diverse information. In comparing ketamine and esketamine within the original Risk of Bias (RoB) framework, we find that the two treatments exhibit largely similar characteristics across the various domains. The notable exception is in the performance domain where esketamine studies received more “Some concerns” ratings than ketamine studies. Despite not having conducted a detailed analytical comparison, the other differences between esketamine and ketamine studies do not appear to be significantly distinct. Results from Jadad score and Downs & Black Checklist are limited, and their overall scoring may not always be consistent with the outcomes derived from Cochrane’s Risk of Bias assessment.

#### 3.7.2 Outcomes certainty of evidence

Except for Cochrane systematic reviews, almost all studies did not estimate the level of certainty of the evidence. Specifically, the studies measured the degree of certainty of the evidence as follows: Dean et al. (57) reported a low and very low degree of certainty for the response at 24–48 h when comparing ketamine vs. saline and ketamine vs. midazolam, respectively. The study also found a very low certainty of evidence for depressive symptoms at 24–48 h and 7–13 day time points, as well as a very low confidence level for total dropouts at endpoint and remission at both 24–48 h and 7–13 days. Caddy et al. (42) identified a low level of certainty for the response measure at the 24–48 h, 3–6 day, and 7–13 day time points, as well as a low level of evidence for depressive symptoms at the 24–48 h time point and emotional blunting at endpoint. Witt et al. (62) discovered a moderate degree of evidence for suicide rate at two time points: <60 min and 14–28 days. Finally, Zheng et al. (56) found a high level of evidence at endpoints for response, remission, and nearly all investigated adverse effects.

## 4 Discussion

### 4.1 Main findings

To the best of our knowledge, this OoR is the most comprehensive to date available, encompassing a total of 26 studies. In comparison to previous OoRs (29, 30), a particularly accurate selection process for reviews based on the original included studies was employed. Consequently, we excluded some outcomes or entire meta-analyses that did not meet the inclusion criteria, thus resulting in an enhanced methodological and data homogeneity.

As a whole, existing data confirm the rapid efficacy of antidepressant treatment of ketamine on affective symptoms and suicidal ideation, though the effect on the latter decreases at later time points. There is no available data on depressive symptoms separately for patients with unipolar and bipolar depression for the time points < 60 min, 60–90 min, and 90–120 min. Combined meta-analyses of patients with unipolar and bipolar depression indicate greater efficacy of the intervention compared to the control group. For subsequent time points, the intervention maintains good efficacy for patients with unipolar depression, whereas its efficacy declines after 2 weeks in patients with bipolar depression.

Regarding tolerability and acceptability, data is limited. Nevertheless, no significant difference emerges between intervention and control groups, except for adverse effects. Overall, however, the quality of the original studies included in the meta-analyses is poor.

Of note, all meta-analyses focusing solely on esketamine, which often shows to be more effective than the control across several outcomes, only include patients with unipolar depression. Conversely, the data for ketamine, which can display more inconsistent efficacy results, considers both patients with unipolar and bipolar depression. This leaves unresolved the question of efficacy between ketamine and its enantiomer. Indeed, the solitary study that directly contrasts esketamine and ketamine echoes this deficiency in data, reporting no substantial differences in either efficacy or tolerability between the two treatments (74). An analysis of study quality revealed that ketamine and esketamine have comparable Risk of Bias across most domains. One exception is the allocation concealment, where esketamine outperforms due to its differing administration route. However, preliminary data show no efficacy differences between ketamine and esketamine in patients with MDD, when both are administered intravenously in a triple-blind study (75).

### 4.2 Evidence in context

The available evidence for the treatment of TRD and for patients at suicidal risk offers viable alternatives (76–80); however, its prevalence and burden remain high (81). Our meta-summary highlighted the efficacy of the use of ketamine/esketamine in these clinical contexts, although the quality of the evaluated evidence is low. Despite its potential as a promising intervention, there are notable challenges associated with its use, including the requirement for hospital visits for administration and the restriction on driving after receiving the treatment. Additionally, the substantial costs involved in initiating and maintaining the treatment, which impact the healthcare system, should be considered. Indeed, according to NICE guidelines, the use of esketamine would have a too much high incremental cost-effectiveness ratio, leading to discontinuation of this approach even when adopted as a third-line intervention (82). Additionally, other studies showed how other therapeutic options had a better cost-effectiveness ratio in the treatment of patients with TRD, such as electroconvulsive therapy (83).

### 4.3 Limitations of the evidence

The available evidence does not allow to draw conclusions with a high level of confidence. Specifically, no available meta-analysis holds up to high quality criteria. In addition, almost all the included original studies had various methodological limitations, leading few studies to have a low risk of bias. In addition, few meta-analyses investigated the long-term efficacy of ketamine, thus leaving an evidence gap.

### 4.4 Implication for practice, policy and future research

At present, no guidelines recommend ketamine or esketamine as a treatment for depression, except as a third-line intervention, due to the limited available data. Consequently, in clinical practice, it is crucial to carefully consider the use of ketamine or esketamine against other interventions with a higher certainty of evidence. However, given the potential of ketamine treatment, especially for TRD and high suicidal risk cases, further research in ketamine is warranted. The two key priorities should be: (i) more methodologically rigorous studies, and (ii) long-term data on treatment efficacy.

### 4.5 Strengths and limitations of the overview

To our knowledge, the present OoR is the most extensive available evidence on ketamine for the treatment of depression. As such, this work has some strengths: (i) it is based on current standards regarding the preparation of OoRs, setting it apart from previous studies; (ii) it not only draws from bibliographic search engines, but also from aggregators of systematic reviews; (iii) we reviewed the individual studies included in various meta-analyses to improve the methodological homogeneity of the reported data; additionally, we performed a comprehensive and detailed representation of the data related to the side effects; and (iv) we also tried to synthesize the available data clearly and transparently, reporting both the excluded and included material.

This OoR has also limitations: (i) the literature review was not conducted on multiple search engines, although, compared to previous similar works, we included more than twice the number of studies; (ii) we only included studies written in English during the selection process; (iii) the attempt to be more comprehensive may have led to the possibility of combining heterogeneous reviews on one hand and having studies with similar inclusion criteria on the other, thus raising the risk of duplicated information; during this process, however, particular attention has been paid to disentangle the different research questions, to provide the reader with as much useful information as possible for clinical practice and to improve future research based on the present data; and (iv) we have not undertaken a detailed comparison of esketamine and ketamine’s effectiveness or tolerability. Nevertheless, our findings suggest esketamine has a more consistent advantage over control treatments. However, this conclusion should be interpreted cautiously due to the smaller number of studies pertaining to esketamine compared to those on ketamine. Interestingly, despite esketamine studies having undergone a rigorous registration process, the quality of these studies did not significantly surpass that of ketamine research which has not been subject to such stringent scrutiny. At present, the scarce esketamine-specific meta-analyses, the similar study quality between ketamine and esketamine research, and the variability within the ketamine data, collectively impede drawing any definitive conclusions regarding their comparative efficacy and tolerability, at least for patients with unipolar depression.

## 5 Conclusion and future outlooks

Although literature data suggest that ketamine and its derivatives is effective for treating depression, the available literature remains qualitatively limited. The production of evidence synthesis studies has been prolific; however, it has not improved the overall quality of the original studies, which remains poor. Additionally, concerns about long-term treatment efficacy data persist. Higher quality original studies are needed, particularly with improvements to allocation concealment and assessor blinding in future research. Though the quantity of available data for esketamine is lesser than that for ketamine, it’s crucial not to disregard its apparent consistent efficacy. This effectiveness could be attributed to the selection of a more uniform patient group, specifically those diagnosed with unipolar disorder. Future studies are also warranted to investigate the effectiveness of (es)ketamine in the treatment of major depressive episode with mixed features which appear to be burdened with a higher suicidal risk than pure depressive forms (84). The pharmacological management of mixed states during major depressive episode has always been a challenge for the clinicians not only for their insidious course but also due to the lack of robust evidence (85), that is slowly growing (86). Authors should also enhance data reporting and avoid to selectively present results. Furthermore, it is beneficial for future systematic reviews with meta-analyses to be pre-planned and have registered protocols. Addressing the risk of bias and publication bias in future reviews will provide more valid information on the reliability of the results. Lastly, given the commercial interest in these products for treating depression, the funding of original studies should not be overlooked. In a few words, only when the quality of evidence will reach a sufficient level of evidence, firm conclusions will be drawn about the benefit of using ketamine for the treatment of resistant depression and for patients at suicidal risk.

## Data availability statement

The original contributions presented in the study are included in the article/Supplementary material, further inquiries can be directed to the corresponding author.

## Author contributions

AR: Conceptualization, Data curation, Formal analysis, Investigation, Methodology, Project administration, Visualization, Writing – original draft, Writing – review & editing. PC: Data curation, Investigation, Methodology, Writing – original draft. ADF: Data curation, Formal analysis, Investigation, Methodology, Writing – original draft, Writing – review & editing. AA: Writing – original draft. GC: Investigation, Writing – original draft. CC: Writing – original draft. AC: Writing – original draft. AF: Supervision, Writing – review & editing. GL: Writing – original draft. LM: Writing – original draft. AN: Investigation, Writing – original draft. LR: Investigation, Writing – original draft. AP: Supervision, Writing – original draft. MS: Project administration, Supervision, Writing – review & editing. EA: Project administration, Supervision, Writing – review & editing.
