# Supplementary material for: Efficacy and safety of ketamine and esketamine for unipolar and bipolar depression: an overview of systematic reviews with meta-analysis
Source: Front Psychiatry. 2024 Feb 1;15:1325399. doi: 10.3389/fpsyt.2024.1325399 (PMC10867194; doi:10.3389/fpsyt.2024.1325399)
Supplement: Supplementary file 1 [file Data_Sheet_1.DOCX]

Supplementary Material

**Efficacy and safety of ketamine and esketamine for unipolar and bipolar depression: an overview of systematic reviews with meta-analysis**

**Alessandro Rodolico^1^****^,†,*^, Pierfelice Cutrufelli^1,†^, Antonio Di Francesco^1,†^, Andrea Aguglia^2,3^, Gaetano Catania^4^, Carmen Concerto^1^, Alessandro Cuomo^5^, Andrea Fagiolini^5^, Giuseppe Lanza^6,7^, Ludovico Mineo^1^, Antimo Natale^1,8^, Laura Rapisarda^9^, Antonino Petralia^1^, Maria Salvina Signorelli^1^, Eugenio Aguglia^1^**

^†^Alessandro Rodolico, Pierfelice Cutrufelli and Antonio Di Francesco contributed equally to this study and share first authorship.

^1^Department of Clinical and Experimental Medicine, Institute of Psychiatry, University of Catania, Catania, Italy

^2^Department of Neuroscience, Rehabilitation, Ophthalmology, Genetics, Maternal and Child Health, Section of Psychiatry, University of Genoa, Genoa, Italy

^3^IRCCS Ospedale Policlinico San Martino, Genoa, Italy

^4^University of Catania, Catania, Italy

^5^Department of Molecular Medicine, University of Siena, Siena, Italy

^6^Department of Surgery and Medical-Surgical Specialties, University of Catania, Catania, Italy

^7^Clinical Neurophysiology Research Unit, Oasi Research Institute-IRCCS, Troina, Italy

^8^Department of Psychiatry, Adult Psychiatry Service (SPA), University Hospitals of Geneva (HUG), Geneva, Switzerland

^9^Department of Biomedical and Biotechnological Sciences, Section of Pharmacology, University of Catania,Italy

*** Correspondence:**Alessandro Rodolico, Department of Clinical and Experimental Medicine, Psychiatry Unit, University of Catania, Via Santa Sofia 78, 95123 Catania, Italy. Email: [alessandro.rodolico@phd.unict.it](mailto:alessandro.rodolico@phd.unict.it); ORCID: 0000-0003-2196-0601

Summary

[1 Supplementary Data 1](#_Toc145847138)

[1.1 Supplementary Figure 1. PRISMA flowchart 2](#_Toc145847139)

[1.2 Supplementary Table 1. Included systematic reviews 3](#_Toc145847140)

[1.3 Supplementary Table 2. Excluded systematic reviews 10](#_Toc145847141)

[1.4 Supplementary Table 3. Table of the included randomized controlled trials 17](#_Toc145847142)

[1.5 Supplementary Table 4. Table of the wrongly included studies 33](#_Toc145847143)

[1.6 Supplementary Table 5. Extended outcomes table 36](#_Toc145847144)

[1.7 Supplementary Table 6. Risk of bias of the original studies 127](#_Toc145847145)

[1.8 Supplementary Table 7. Systematic reviews’ quality assessment (AMSTAR 2) 144](#_Toc145847146)

[1.9 Supplementary Table 8. Main analysis 149](#_Toc145847147)

[1.10 Supplementary Tables 9. Citation matrices 163](#_Toc145847148)

[1.11 Supplementary 10. Deviation from protocol 174](#_Toc145847149)

## Supplementary Figure 1. PRISMA flowchart

**Identification of studies via databases and registers**

Records identified from*:

Databases (n =2256)

Records removed *before screening*:

Duplicate records removed (n =486 )

**Identification**

Records screened

(n =1770 )

Records excluded**

(n =1715 )

Reports excluded: 29

Population undergoing ECT 1 (n =8 )

No control group is required, no only-RCT outcomes available (n =9 )

Wrong population (n =6 )

No meta-analysis (n=3)

Wrong intervention (n=1)

Wrong outcome (n=1)

Other (n=1)

Reports assessed for eligibility

(n =55)

**Screening**

**Included**

Studies included in review

(n =26 )

## Supplementary Table 1. Included systematic reviews

| **Simple ID** | **Search date and search engines** | **Intervention** | | **Dose Range** | | **Comparator** | **Outcomes** | **Funding** |
| --- | --- | --- | --- | --- | --- | --- | --- | --- |
| Population (by eligibility):   - Major Depressive Disorder or Bipolar Disorder - Resistant: Not specified - Suicidality: Not specified | | | | | | | | |
| Rhee 2022 [1] | Last search date: 2022-04-19  Bibliographic search engines: PubMed/MEDLINE, Cochrane Library, Embase, [ClinicalTrials.gov](http://ClinicalTrials.gov), EU Clinical Trials Register, Google Scholar | Ketamine (Any route) | | IV: 0.5mg/kg  IM: 0.5mg/kg | | ECT | Efficacy - Depressive symptoms | Not reported |
| McIntyre 2020 [2] | Last search date: 2019-01-24  Bibliographic search engines: PubMed, Google Scholar | Ketamine IV, Ketamine IN, Ketamine Oral | | IV: 0.1-1mg/kg  IN: 28-84mg  Oral: 1-150mg | | Placebo/Active placebo | Efficacy - Depressive symptoms | No-Funding |
| Bahji 2022 [3] | Last search date: 2021-11-23  Bibliographic search engines: Pubmed/MEDLINE, Embase, PsycInfo, Cochrane Central Register of Controlled Clinical Trials | Ketamine (Any route)  Esketamine (Any route) | | Ketamine IV: 0.1-0.5mg/kg  Ketamine oral: 50mg and 1mg/kg  Esketamine IN: 28-100mg | | Placebo/Active placebo | Efficacy - Response, Efficacy - Remission, Efficacy - Depressive symptoms, Retention in treatment, Tolerability - Dropouts due to adverse events, Tolerability - Adverse events (Total), Tolerability - Dizziness, Tolerability - Hypertension, Tolerability - Nausea, Tolerability - Vertigo, Tolerability - Somnolence, Tolerability - Hypoestesia, Tolerability - Paresthesia, Tolerability - Dissociative Symptoms, Tolerability - Anxiety, Tolerability - Dysgeusia, Tolerability - Headache | Public |
| Fond 2014 [4] | Last search date: 2013-09-15  Bibliographic search engines: PubMed, Embase, Cochrane, PsycInfo, BIOSIS, Science Direct, ProQuest Dissertations | Ketamine (Route not specified) | | NA | | Placebo/Active placebo | Efficacy - Depressive symptoms | Public |
| Han 2016 [5] | Last search date: 2016-04-15  Bibliographic search engines: PubMed, CENTRAL, Web of Science, Embase, CBM-disc, CNKI, World Health Organization’s trials portal (ICTRP) | Ketamine (Route not specified) | | IV: 0.5-0.54mg/kg | | Placebo/Active placebo | Efficacy - Response, Efficacy - Remission | Public |
| Kishimoto 2016 [6] | Last search date: 2015-08-25  Bibliographic search engines: PubMed, Web of Science, ClinicalTrials.gov, PsycInfo | Ketamine IV | | IV: 0.1-0.54mg/kg | | Placebo/Active placebo | Efficacy - Depressive symptoms, Efficacy - Response, Efficacy - Remission, Tolerability - BPRS, Acceptability - Total dropouts, Tolerability - CADSS | Combined |
| McGirr 2015 [7] | Last search date: 2014-01-14  Bibliographic search engines: MEDLINE, Embase, PsycInfo, CENTRAL | Ketamine IV, Ketamine IN,  Ketamine Oral, Ketamine parenteral | | IV: 0.5mg/kg/ - 0.54mg/kg  IN: 50mg | | Placebo/Active placebo | Efficacy - Remission, Efficacy - Response, Efficacy - Depressive symptoms, Acceptability - Total dropouts, Tolerability - BPRS, Tolerability - CADSS | No-Funding |
| Newport 2015 [8] | Last search date: 2015-05-15  Bibliographic search engines: MEDLINE, PsycInfo, Cochrane Central Register of Controlled Clinical Trials, Cumulative Index to Nursing and Allied Health Literature, Google Scholar, ClinicalTrials.gov | Ketamine (Any route) | | IV: 0.4-0.54mg/kg  IN: 0.5mg | | Placebo/Active placebo | Efficacy - Remission, Efficacy - Response, Tolerability - BPRS, Tolerability - CADSS | Not reported |
| Nuñez 2020 [9] | Last search date: 2020-04-07  Bibliographic search engines: Scopus, Ovid MEDLINE, Ovid Embase, Ovid PsycInfo, Ovid CCRCCT, Ovid CDSR | Ketamine Oral | | Oral: 0.1mg/kg x 3/week OR 50-150mg/day | | Placebo/Active placebo | Efficacy - Depressive symptoms, Efficacy - Remission, Efficacy - Response, Tolerability - Adverse events (Total), Tolerability - Gastrointestinal, Tolerability - Neurological, Tolerability - Psychiatric | Combined |
| Romeo 2015 [10] | Last search date: 2013-12-15  Bibliographic search engines: PsycInfo, MEDLINE | Ketamine (Any route) | | IV: 0.5-0.54mg/kg  IN: 50mg | | Placebo | Efficacy - Depressive symptoms, Tolerability - BPRS | Not reported |
| Xiong 2021 [11] | Last search date: 2020-07-01  Bibliographic search engines: PubMed, MEDLINE, Embase, Cochrane | Esketamine IN, Ketamine IV | | IN:84mg  IV: 0.2-0.5mg/kg | | Placebo/Active placebo | Efficacy - Suicide scales | No-Funding |
| Lee 2015 [12] | Last search date: 2013-09-10  Bibliographic search engines: PubMed, PsycInfo | Ketamine (Route not specified) | | IV: 0.5mg/kg - 0.54mg/kg  IN: 50mg | | Placebo/Active placebo | Efficacy - Depressive symptoms | Not reported |
| Marcantoni 2020 [13] | Last search date: 2019-01-04  Bibliographic search engines: PubMed, Embase, MEDLINE, PsycInfo, CINAHL, Cochrane Central Register of Controlled Clinical Trials | Ketamine IV | | NR | | Placebo/Active placebo | Efficacy - Depressive symptoms, Efficacy - Response, Efficacy - Remission | No-Funding |
| Population (by eligibility):   - Bipolar Disorder - Resistant: Not specified - Suicidality: Not specified | | | | | | | | |
| Bahji 2021 [14] | Last search date: 2020-04-15  Bibliographic search engines: CINAHL, Embase, LILACS database, PsycInfo, PubMed/ MEDLINE, World Health Organization's International Clinical Trials Registry Platform and Clinical Trials, Cochrane Central Register of Controlled Clinical Trials, Pre-CINAHL | NMDAR antagonists IV  NMDAR antagonists oral | |  | | Placebo/Active placebo | Efficacy - Response, Efficacy - Remission, Efficacy - Depressive symptoms | No-Funding |
| Dean 2021 [15] | Last search date: 2015-01-09  Bibliographic search engines: CCDANCTR, ClinicalTrials.gov, World Health Organization’s trials portal (ICTRP), GSK Study Register, Lilly Trial Guide, US Food and Drug Administration, Medicines and Healthcare products Regulatory Agency (UK),European Medicines Agency, Pharmaceuticals and Medical Devices Agency (Japan),Therapeutic Goods Administration (Australia),Embase, PsycInfo, MEDLINE, CENTRAL, CCMDCTR | Ketamine (Route not specified) | | IV: 0.5mg/kg/min | | Placebo/Active placebo | Efficacy - Response, Efficacy - Remission, Efficacy - Depressive symptoms, Acceptability - Total dropouts, Acceptability - Lack of efficacy, Efficacy - Suicide scales | Public |
| Population (by eligibility):   - Major Depressive Disorder - Resistant: Not specified - Suicidality: Not specified | | | | | | | | |
| Papakostas 2020 [16] | Last search date 15/01/2019  Bibliographic search engines: Pubmed | Esketamine IN | |  | | Placebo/Active placebo | Response, Remission, Mean change in scores from baseline and their corresponding standard deviations | No-Funding |
| Zheng 2020 [17] | Last search date: 1/08/2019  Bibliographic search engines: Pubmed, Embase, Psycinfo, Cochrane Library, WangFang, Chinese Journal Net | Esketamine IN | |  | | Placebo/Active placebo | Response, Remission | Public |
| Hock 2022 [18] | Last search date: 30-11-2020  Bibliographic search engines: Pubmed, ClinicalTrials.gov | Esketamine IN | | 28-84mg | | Placebo/Active placebo | Efficacy - Depressive symptoms | Public |
| Jawad 2022 [19] | Last search date: 2021-12-01  Bibliographic search engines: MEDLINE, Embase classic, Embase, Pharmaceuticals and Medical Devices Agency (Japan), PsycInfo, Ovid Healthstar, Journal@Ovid Full text, Cochrane, Google Scholar, CINAHL | Esketamine IN | |  | | Placebo/Active placebo | Efficacy - Depressive symptoms, Efficacy - Remission, Efficacy - Response, Tolerability - Anxiety, Tolerability - Blurred Vision, Tolerability - Confusion, Tolerability - Constipation, Tolerability - Diarrhea, Tolerability - Diplopia, Tolerability - Dissociation, Tolerability - Dizziness, Tolerability - Dry mouth, Tolerability - Dysgeusia, Tolerability - Euphoria, Tolerability - Fatigue, Tolerability - Feeling drunk, Tolerability - Headache, Tolerability - Hypoestesia, Tolerability - Hypoestesia oral, Tolerability - Increased blood pressure, Tolerability - Insomnia, Tolerability - Nasal Discomfort, Tolerability - Nausea, Tolerability - Oral paresthesia, Tolerability - Postural Dizziness, Tolerability - Sedation, Tolerability - Somnolence, Efficacy - Suicidal ideation, Tolerability - Throat irritation, Tolerability - Vertigo, Tolerability - Viral upper respiratory infection, Tolerability - Vomiting, Tolerability - Oropharyngeal pain, Tolerability - Paresthesia | Not reported |
| Caddy 2015 [20] | Last search date: 2015-01-09  Bibliographic search engines: Cochrane Depression, Anxiety and Neurosis Review Group’s Specialized Register (CCDANCTR), World Health Organization’s trials portal (ICTRP), ClinicalTrials.gov, US Food and Drug Administration, Medicines and Healthcare products Regulatory Agency (UK),European Medicines Agency Pharmaceuticals and Medical Devices Agency (Japan),Therapeutic Goods Administration (Australia) | Glutamate receptor modulators (Any route) | | IV: 0.4-0.54mg/kg  IN: 50mg | | Placebo/Active placebo/ECT | Efficacy - Response, Efficacy - Remission, Acceptability - Total dropouts, Tolerability - Dissociative Symptoms, Tolerability - Hallucinations, Efficacy - Depressive symptoms, Efficacy - Suicide scales, Tolerability - Confusion, Tolerability - Poor co-ordination, Tolerability - Tinnitus | Public |
| An 2021 [21] | Last search date: 2020-04-01  Bibliographic search engines: MEDLINE, Embase, Cochrane Library databases | Esketamine IN, Ketamine IN | |  | | Placebo | Efficacy - Depressive symptoms, Efficacy - Remission, Efficacy - Response, Tolerability - Dizziness, Tolerability - Dysgeusia, Tolerability - Vertigo, Tolerability - Nausea | Public |
| Kryst 2020 [22] | Last search date: 2019-02-22  Bibliographic search engines: Embase, Trip Database, PubMed, CENTRAL | Ketamine (Any route) | | IV: 0.1-1mgkg  Oral:  - 50mg/day  - 1mg/kg | | Placebo/Active placebo | Efficacy - Depressive symptoms, Efficacy - Response, Efficacy - Remission | No-Funding |
| Xu 2016 [23] | Last search date: 2014-08-15  Bibliographic search engines: MEDLINE, PsycInfo, Embase | Ketamine (Route not specified) | | IV: 0.1-0.54mg/kg  IN: 50mg | | Placebo/Active placebo | Efficacy - Response, Efficacy - Remission, Efficacy - Suicide scales, Efficacy - Depressive symptoms | Combined |
| Population (by eligibility):   - Major Depressive Disorder - Resistant: Yes - Suicidality: Not specified | | | | | | | | |
| Papadimitropoulou 2017 [24] | Last search date: 2013-10-21  Bibliographic search engines: MEDLINE, MEDLINE In-Process, Embase, PsycInfo, EconLit, CENTRAL, CDSR, CMR, DARE, HTAD, NHS EED, [ClinicalTrials.gov](http://ClinicalTrials.gov) | Intervention for treatment resistant depression, including Ketamine, esketamine (not limited to intranasal). | | NR | | Placebo | Efficacy - Depressive symptoms, Efficacy - Response | Private |
| Population (by eligibility)   - Bipolar disorder - Resistant: Yes - Suicidality: Not specified | | | | | | | | |
| Fornaro 2020 [25] | Last search date: 2020-05-25  Bibliographic search engines: PubMed, PsycInfo, ClinicalTrials.gov | | Active interventions for treatment resistant bipolar depression (including ketamine) | | NR | Placebo/Active placebo | Efficacy - Response, Efficacy - Depressive symptoms, Acceptability - Total dropouts | No-Funding |
| Population (by eligibility):   - Any psychiatric disorder - Resistant: Not specified - Suicidality: Yes | | | | | | | | |
| Witt 2020 [26] | Last search date: 2018-12-12  Bibliographic search engines: CENTRAL, Embase, MEDLINE, PsycInfo | Ketamine (Route not specified),  Esketamine (Route not specified) | | SC:  - 0.1 to 0.5mg/kg/min ascending  IV:  - 0.2-0.5mg/kg/min for non-ascending design  IN  - 84mg esketamine  Unknown (From kudoh)  - 1mg/kg | | Placebo/Active placebo | Efficacy - Suicide scales | Combined |

**References**

1. Rhee TG, Shim SR, Forester BP, Nierenberg AA, McIntyre RS, Papakostas GI, et al. Efficacy and Safety of Ketamine vs Electroconvulsive Therapy Among Patients With Major Depressive Episode: A Systematic Review and Meta-analysis. JAMA Psychiatry. 2022 Dec 1;79(12):1162-72.

2. McIntyre RS, Carvalho IP, Lui LMW, Majeed A, Masand PS, Gill H, et al. The effect of intravenous, intranasal, and oral ketamine in mood disorders: A meta-analysis. J Affect Disord. 2020 Nov 1;276:576-84.

3. Bahji A, Zarate CA, Vazquez GH. Efficacy and safety of racemic ketamine and esketamine for depression: a systematic review and meta-analysis. Expert Opin Drug Saf. 2022 Jun;21(6):853-66.

4. Fond G, Loundou A, Rabu C, Macgregor A, Lancon C, Brittner M, et al. Ketamine administration in depressive disorders: a systematic review and meta-analysis. Psychopharmacology (Berl). 2014 Sep;231(18):3663-76.

5. Han Y, Chen J, Zou D, Zheng P, Li Q, Wang H, et al. Efficacy of ketamine in the rapid treatment of major depressive disorder: a meta-analysis of randomized, double-blind, placebo-controlled studies. Neuropsychiatr Dis Treat. 2016;12:2859-67.

6. Kishimoto T, Chawla JM, Hagi K, Zarate CA, Kane JM, Bauer M, et al. Single-dose infusion ketamine and non-ketamine N-methyl-d-aspartate receptor antagonists for unipolar and bipolar depression: a meta-analysis of efficacy, safety and time trajectories. Psychol Med. 2016 May;46(7):1459-72.

7. McGirr A, Berlim MT, Bond DJ, Fleck MP, Yatham LN, Lam RW. A systematic review and meta-analysis of randomized, double-blind, placebo-controlled trials of ketamine in the rapid treatment of major depressive episodes. Psychol Med. 2015 Mar;45(4):693-704.

8. Newport DJ, Carpenter LL, McDonald WM, Potash JB, Tohen M, Nemeroff CB, et al. Ketamine and Other NMDA Antagonists: Early Clinical Trials and Possible Mechanisms in Depression. Am J Psychiatry. 2015 Oct;172(10):950-66.

9. Nuñez NA, Joseph B, Pahwa M, Seshadri A, Prokop LJ, Kung S, et al. An Update on the Efficacy and Tolerability of Oral Ketamine for Major Depression: A Systematic Review and Meta-Analysis. Psychopharmacol Bull. 2020 Sep 14;50(4):137-63.

10. Romeo B, Choucha W, Fossati P, Rotge JY. Meta-analysis of short- and mid-term efficacy of ketamine in unipolar and bipolar depression. Psychiatry Res. 2015 Dec 15;230(2):682-8.

11. Xiong J, Lipsitz O, Chen-Li D, Rosenblat JD, Rodrigues NB, Carvalho I, et al. The acute antisuicidal effects of single-dose intravenous ketamine and intranasal esketamine in individuals with major depression and bipolar disorders: A systematic review and meta-analysis. Journal of psychiatric research. 2021 Feb;134:57-68.

12. Lee EE, Della Selva MP, Liu A, Himelhoch S. Ketamine as a novel treatment for major depressive disorder and bipolar depression: a systematic review and quantitative meta-analysis. Gen Hosp Psychiatry. 2015 Mar-Apr;37(2):178-84.

13. Marcantoni WS, Akoumba BS, Wassef M, Mayrand J, Lai H, Richard-Devantoy S, et al. A systematic review and meta-analysis of the efficacy of intravenous ketamine infusion for treatment resistant depression: January 2009 - January 2019. J Affect Disord. 2020 Dec 1;277:831-41.

14. Bahji A, Ermacora D, Stephenson C, Hawken ER, Vazquez G. Comparative Efficacy and Tolerability of Adjunctive Pharmacotherapies for Acute Bipolar Depression: A Systematic Review and Network Meta-analysis. Can J Psychiatry. 2021 Mar;66(3):274-88.

15. Dean RL, Marquardt T, Hurducas C, Spyridi S, Barnes A, Smith R, et al. Ketamine and other glutamate receptor modulators for depression in adults with bipolar disorder. Cochrane Database Syst Rev. 2021 Oct 8;10(10):CD011611.

16. Papakostas GI, Salloum NC, Hock RS, Jha MK, Murrough JW, Mathew SJ, et al. Efficacy of Esketamine Augmentation in Major Depressive Disorder: A Meta-Analysis. J Clin Psychiatry. 2020 May 26;81(4).

17. Zheng W, Cai DB, Xiang YQ, Zheng W, Jiang WL, Sim K, et al. Adjunctive intranasal esketamine for major depressive disorder: A systematic review of randomized double-blind controlled-placebo studies. J Affect Disord. 2020 Mar 15;265:63-70.

18. Gi, S. HR, Feeney A, Iovieno N, Murrough JW, Mathew SJ, et al. Rapidity of Symptom Improvement With Intranasal Esketamine for Major Depressive Disorder: A Systematic Review and Meta-Analysis. The Journal of clinical psychiatry. 2022 12/12/2022;84(1).

19. Jawad MY, Di Vincenzo JD, Ceban F, Jaberi S, Lui LMW, Gillissie ES, et al. The efficacy and safety of adjunctive intranasal esketamine treatment in major depressive disorder: a systematic review and meta-analysis. Expert Opin Drug Saf. 2022 Jun;21(6):841-52.

20. Caddy C, Amit BH, McCloud TL, Rendell JM, Furukawa TA, McShane R, et al. Ketamine and other glutamate receptor modulators for depression in adults. Cochrane Database Syst Rev. 2015 Sep 23(9):CD011612.

21. An D, Wei C, Wang J, Wu A. Intranasal Ketamine for Depression in Adults: A Systematic Review and Meta-Analysis of Randomized, Double-Blind, Placebo-Controlled Trials. Front Psychol. 2021;12:648691.

22. Kryst J, Kawalec P, Mitoraj AM, Pilc A, Lason W, Brzostek T. Efficacy of single and repeated administration of ketamine in unipolar and bipolar depression: a meta-analysis of randomized clinical trials. Pharmacol Rep. 2020 Jun;72(3):543-62.

23. Xu Y, Hackett M, Carter G, Loo C, Galvez V, Glozier N, et al. Effects of Low-Dose and Very Low-Dose Ketamine among Patients with Major Depression: a Systematic Review and Meta-Analysis. Int J Neuropsychopharmacol. 2016 Apr;19(4).

24. Papadimitropoulou K, Vossen C, Karabis A, Donatti C, Kubitz N. Comparative efficacy and tolerability of pharmacological and somatic interventions in adult patients with treatment-resistant depression: a systematic review and network meta-analysis. Curr Med Res Opin. 2017 Apr;33(4):701-11.

25. Fornaro M, Carvalho AF, Fusco A, Anastasia A, Solmi M, Berk M, et al. The concept and management of acute episodes of treatment-resistant bipolar disorder: a systematic review and exploratory meta-analysis of randomized controlled trials. J Affect Disord. 2020 Nov 1;276:970-83.

26. Witt K, Potts J, Hubers A, Grunebaum MF, Murrough JW, Loo C, et al. Ketamine for suicidal ideation in adults with psychiatric disorders: A systematic review and meta-analysis of treatment trials. Aust N Z J Psychiatry. 2020 Jan;54(1):29-45.

## Supplementary Table 2. Excluded systematic reviews

| **Excluded review** | **Excluding reason** |
| --- | --- |
| Ainsworth 2020 (1) | P: Major Depressive Disorder and Bipolar Disorder  I: Population undergoing ECT |
| Alnefeesi 2022(2) | P: Major Depressive Disorder  I: Ketamine  C: No control group is required, not only-RCT outcome |
| Bartoli 2017 (3) | P: Does not include MDD or BD as inclusion criteria  I: Ketamine  C: No control group is required, not only-RCT outcome. |
| Caddy 2014 (4) | P: Major Depressive Disorder and Bipolar Disorder  I: Ketamine  C: No control group is required, not only-RCT outcome |
| Carter 2020 (5) | P: Major Depressive Disorder  I: Any treatment for treatment resistant Major Depressive Disorder  C: It includes all drugs targeting NMDA in the same node as intervention (not ketamine only) |
| Conley 2021(6) | P: Major Depressive Disorder and Bipolar Disorder  I: Ketamine  C: No control group is required, not only-RCT outcome |
| Coyle 2015 (7) | P: Major Depressive Disorder and Bipolar Disorder  I: Ketamine  C: No control group is required, not only-RCT outcome |
| Fond 2016 (8) | P: Major Depressive Disorder and Bipolar Disorder  I: Population undergoing ECT |
| Huang 2022 (9) | P: Inclusion criteria allows non-mood disorders; no outcomes including only mood disorders patients; |
| Kato 2021 (10) | P: Major Depressive Disorder and Bipolar Disorder  I: It does not investigate the use of ketamine but discontinuation of drugs |
| Li 2017 (11) | P: Major Depressive Disorder and Bipolar Disorder  I: Population undergoing ECT |
| McGirr 2015 (12) | P: Major Depressive Disorder and Bipolar Disorder  I: Population undergoing ECT |
| McGirr 2017 (13) | P: Major Depressive Disorder and Bipolar Disorder  I: Population undergoing ECT |
| Memon 2020 (14) | D: It isn't a meta-analysis |
| Meshkat 2022 (15) | P: It includes only pediatric population |
| Pang 2020 (16) | P: Patients without a diagnosis of MDD or BD; (some studies in chinese not retrievable or not understandable) |
| Papadimitropoulou 2015 (17) | P: Major Depressive Disorder  I: Any treatment for treatment resistant Major Depressive Disorder  C: Any treatment for treatment resistant Major Depressive Disorder  O: Ketamine Data not reported |
| Price 2022 (18) | P: Inclusion criteria allows non-mood disorders; no outcomes including only mood disorders patients; |
| Ren 2018 (19) | P: Major Depressive Disorder and Bipolar Disorder  I: Population undergoing ECT |
| Romeo 2016 (20) | P: Major Depressive Disorder  I: Population undergoing ECT |
| Rosenblat 2019 (21) | D: No meta-analysis |
| Scott 2022 (22) | P: Major Depressive Disorder  I: Any treatment for treatment resistant Major Depressive Disorders  C: No control group is required, no only-RCT outcome; |
| Smith-Apeldoorn 2022 (23) | D: No meta-analysis |
| Tashakkori 2021 (24) | P: Major Depressive Disorder and Bipolar Disorder  I: Ketamine  C: No control group is required, no only-RCT outcome; |
| Wang 2022 (25) | P: Patients were not required to have a depression diagnosis in some of the RCT |
| Webler 2019 (26) | P: Not reported  I: Ketamine  C: No control group is required, no only-RCT outcome |
| Wilkinson 2018 (27) | P: Any psychiatric disorder  I: Ketamine  C: Saline placebo or midazolam  O: It includes Ballard 2015 and Valentine 2011 which we considered non-RCT. No only-RCT outcomes available. |
| Yuan 2020 (28) | P: Major Depressive Disorder  I: Not specified if only RCT were eligible in eligible criteria.  Some of the outcomes contain ECT intervention (Loo 2012 and Ray Griffiths 2017) |
| Zheng 2019 (29) | P: Major Depressive Disorder and Bipolar Disorder  I: Population undergoing ECT |

**References**

1. Ainsworth NJ, Sepehry AA, Vila-Rodriguez F. Effects of Ketamine Anesthesia on Efficacy, Tolerability, Seizure Response, and Neurocognitive Outcomes in Electroconvulsive Therapy: A Comprehensive Meta-analysis of Double-Blind Randomized Controlled Trials. J ECT. 2020;36(2):94-105.

2. Alnefeesi Y, Chen-Li D, Krane E, Jawad MY, Rodrigues NB, Ceban F, et al. Real-world effectiveness of ketamine in treatment-resistant depression: A systematic review & meta-analysis. Journal of psychiatric research. 2022;151:693-709.

3. Bartoli F, Riboldi I, Crocamo C, Di Brita C, Clerici M, Carra G. Ketamine as a rapid-acting agent for suicidal ideation: A meta-analysis. Neurosci Biobehav Rev. 2017;77:232-6.

4. Caddy C, Giaroli G, White TP, Shergill SS, Tracy DK. Ketamine as the prototype glutamatergic antidepressant: pharmacodynamic actions, and a systematic review and meta-analysis of efficacy. Ther Adv Psychopharmacol. 2014;4(2):75-99.

5. Carter B, Strawbridge R, Husain MI, Jones BDM, Short R, Cleare AJ, et al. Relative effectiveness of augmentation treatments for treatment-resistant depression: a systematic review and network meta-analysis. Int Rev Psychiatry. 2020;32(5-6):477-90.

6. Conley AA, Norwood AEQ, Hatvany TC, Griffith JD, Barber KE. Efficacy of ketamine for major depressive episodes at 2, 4, and 6-weeks post-treatment: A meta-analysis. Psychopharmacology (Berl). 2021;238(7):1737-52.

7. Coyle CM, Laws KR. The use of ketamine as an antidepressant: a systematic review and meta-analysis. Hum Psychopharmacol. 2015;30(3):152-63.

8. Fond G, Bennabi D, Haffen E, Brunel L, Micoulaud-Franchi JA, Loundou A, et al. A Bayesian framework systematic review and meta-analysis of anesthetic agents effectiveness/tolerability profile in electroconvulsive therapy for major depression. Sci Rep. 2016;6:19847.

9. Huang X, Harris LM, Funsch KM, Fox KR, Ribeiro JD. Efficacy of psychotropic medications on suicide and self-injury: a meta-analysis of randomized controlled trials. Transl Psychiatry. 2022;12(1):400.

10. Kato H, Koizumi T, Takeuchi H, Tani H, Mimura M, Uchida H. Effects of Discontinuation of Drugs Used for Augmentation Therapy on Treatment Outcomes in Depression: A Systematic Review and Meta-analysis. Pharmacopsychiatry. 2021;54(3):106-16.

11. Li DJ, Wang FC, Chu CS, Chen TY, Tang CH, Yang WC, et al. Significant treatment effect of add-on ketamine anesthesia in electroconvulsive therapy in depressive patients: A meta-analysis. Eur Neuropsychopharmacol. 2017;27(1):29-41.

12. McGirr A, Berlim MT, Bond DJ, Neufeld NH, Chan PY, Yatham LN, et al. A systematic review and meta-analysis of randomized controlled trials of adjunctive ketamine in electroconvulsive therapy: efficacy and tolerability. Journal of psychiatric research. 2015;62:23-30.

13. McGirr A, Berlim MT, Bond DJ, Chan PY, Yatham LN, Lam RW. Adjunctive ketamine in electroconvulsive therapy: updated systematic review and meta-analysis. Br J Psychiatry. 2017;210(6):403-7.

14. Memon RI, Naveed S, Faquih AE, Fida A, Abbas N, Chaudhary AMD, et al. Effectiveness and Safety of Ketamine for Unipolar Depression: a Systematic Review. Psychiatr Q. 2020;91(4):1147-92.

15. Meshkat S, Rosenblat JD, Ho RC, Rhee TG, Cao B, Ceban F, et al. Ketamine use in pediatric depression: A systematic review. Psychiatry Research. 2022;317.

16. Pang L, Cui M, Dai W, Kong J, Chen H, Wu S. Can Intraoperative Low-Dose R,S-Ketamine Prevent Depressive Symptoms After Surgery? The First Meta-Analysis of Clinical Trials. Front Pharmacol. 2020;11:586104.

17. Papadimitropoulou K, Vossen C, Karabis A, Donatti C, Kubitz N. Comparative Efficacy Of Ketamine And Other Pharmacological And Somatic Interventions In Adult Patients With Treatment-Resistant Depression: A Network Meta-Analysis. Value in Health. 2015;18(7).

18. Price RB, Kissel N, Baumeister A, Rohac R, Woody ML, Ballard ED, et al. International pooled patient-level meta-analysis of ketamine infusion for depression: In search of clinical moderators. Mol Psychiatry. 2022;27(12):5096-112.

19. Ren L, Deng J, Min S, Peng L, Chen Q. Ketamine in electroconvulsive therapy for depressive disorder: A systematic review and meta-analysis. Journal of psychiatric research. 2018;104:144-56.

20. Romeo B, Choucha W, Fossati P, Rotge JY. Time-Course of Depression Improvement With Ketamine Adjunction in Electroconvulsive Therapy. J ECT. 2016;32(2):80-1.

21. Rosenblat JD, Carvalho AF, Li M, Lee Y, Subramanieapillai M, McIntyre RS. Oral Ketamine for Depression: A Systematic Review. J Clin Psychiatry. 2019;80(3).

22. Scott F, Hampsey E, Gnanapragasam S, Carter B, Marwood L, Taylor RW, et al. Systematic review and meta-analysis of augmentation and combination treatments for early-stage treatment-resistant depression. J Psychopharmacol. 2023;37(3):268-78.

23. Smith-Apeldoorn SY, Veraart JK, Spijker J, Kamphuis J, Schoevers RA. Maintenance ketamine treatment for depression: a systematic review of efficacy, safety, and tolerability. Lancet Psychiatry. 2022;9(11):907-21.

24. Tashakkori M, Ford A, Dragovic M, Gabriel L, Waters F. The time course of psychotic symptom side effects of ketamine in the treatment of depressive disorders: a systematic review and meta-analysis. Australas Psychiatry. 2021;29(1):80-7.

25. Wang J, Sun Y, Ai P, Cui V, Shi H, An D, et al. The effect of intravenous ketamine on depressive symptoms after surgery: A systematic review. J Clin Anesth. 2022;77:110631.

26. Webler R, Wilkinson S, Kirwin D, Bloch M, Kitay B, Sanacora G. S128. Expectancy and Ketamine’s Rapid Antidepressant Effect: A Meta-Analysis. Biological Psychiatry. 2019;85(10).

27. T. WS, Ballard ED, Bloch MH, Mathew SJ, Murrough JW, Feder A, et al. The Effect of a Single Dose of Intravenous Ketamine on Suicidal Ideation: A Systematic Review and Individual Participant Data Meta-Analysis. The American journal of psychiatry. 2018;175(2).

28. Yuan Z, Chen Z, Xue M, Zhang J, Leng L. Application of antidepressants in depression: A systematic review and meta-analysis. J Clin Neurosci. 2020;80:169-81.

29. Zheng W, Li XH, Zhu XM, Cai DB, Yang XH, Ungvari GS, et al. Adjunctive ketamine and electroconvulsive therapy for major depressive disorder: A meta-analysis of randomized controlled trials. J Affect Disord. 2019;250:123-31.

## Supplementary Table 3. Table of the included randomized controlled trials

| **Study Name** | **Participants** | **Treatment resistant major depression definition** | **Suicidality Definition** | **Interventions and control conditions** |
| --- | --- | --- | --- | --- |
| **Treatment resistant and with suicidal ideation** | | | | |
| Ionescu 2019 [1]  Study design: parallel | Sample size: 26  Diagnosis: Unipolar Depression  Treatment resistant: Included  Suicidal ideation: Included | History of ≥ 3 failed antidepressant treatment trials of adequate dose and duration during the current episode (including the current regimen), as measured by the MGH Antidepressant Treatment History Questionnaire (ATHQ); | Suicidal ideation for ≥ 3 months (as measured by ≥ 1 on the Columbia Suicide Severity Rating Scale (C-SSRS) SI score (Posner et al., 2011) without the requirement for immediate hospitalization, and have a HDRS suicide item score ≥ 2 (current SI, thoughts of own death) at screening or one of the other two pre-infusion phase visits | Intervention: Ketamine IV  Administration strategy: Augmentation of current therapy  Comparator: Saline |
| **Treatment resistant and without suicidal ideation** | | | | |
| Daly 2018 [2]  Study design: parallel multiarm | Sample size: 67  Diagnosis: Unipolar Depression  Treatment resistant: Included  Suicidal ideation: Excluded | Inadequate response to 2 or more antidepressants (assessed by Massachusetts General Hospital Antidepressant Treatment Response Questionnaire 21), with at least 1 inadequate response in the current depression episode. Otherwise, an antidepressant failure from a prior episode was acceptable. | No recent or current suicidal ideation with intent to act | Intervention: Esketamine IN  3 dosages: 28mg, 56mg, 84mg  Administration strategy: Augmentation of current therapy  Comparator: Saline |
| Diazgranados 2010 [3]  Study design: crossover | Sample size: 18  Diagnosis: Bipolar Depression  Treatment resistant: Included  Suicidal ideation: Excluded | Subjects were required to have a score of 20 or more on the Montgomery-Asberg Depression Rating Scale (MADRS) at screening and at the start of each ketamine or placebo infusion. Patients were also required to have a current major depressive episode of at least 4 weeks, to have previously failed at least 1 adequate antidepressant trial (as assessed by the Antidepressant Treatment History Form, modified32), and to have failed a prospective open trial of a mood stabilizer while at the NIMH (either lithium or valproate for a minimum period of 4 weeks at therapeutic levels [serum lithium, 0.6-1.2 mEq/L; or valproic acid, 50-125 μg/mL]). | Judged clinically not to be at serious risk of suicide | Intervention: Ketamine IV  Administration strategy: Augmentation of current therapy  Comparator: Saline |
| Fedgchin 2019 [4]  Study design: parallel multiarm | Sample size: 342  Diagnosis: Unipolar Depression  Treatment resistant: Included  Suicidal ideation: Excluded | Non-response to an adequate trial (dose, duration, adherence) of ≥2 antidepressants in the current episode of depression, In addition to at least 1 antidepressant with nonresponse (≤25% improvement) in the current depressive episode based on historical report, nonresponse to a different antidepressant taken at an adequate dose for a total duration of at least 6 weeks was observed prospectively in the screening/prospective observational phase. | No information | Intervention: Esketamine IN  2 Active arms: 56mg, 84mg  Administration strategy: Augmentation of new therapy  Comparator: Saline |
| Galvez 2018 [5]  Study design: parallel | Sample size: 5  Diagnosis: Unipolar Depression  Treatment resistant: Included  Suicidal ideation: Excluded | Insufficient therapeutic response to at least two adequate antidepressant trials during the current depressive episode, assessed through the Antidepressant Treatment History Form | No risk determined by clinical assessment and Columbia Suicide Severity Rating Scale | Intervention: Ketamine IN  Administration strategy: Augmentation of current therapy  Comparator: Midazolam |
| Lally 2014 [6]  Study design: crossover | Sample size: 36  Diagnosis: Bipolar Depression  Treatment resistant: Included  Suicidal ideation: Excluded | All subjects were required to be currently experiencing an MDE lasting at least 4 weeks and to have failed to respond to at least one adequate antidepressant trial before hospital admission, as assessed by the Antidepressant Treatment History Form. | No serious suicidal ideation | Intervention: Ketamine IV  Administration strategy: Augmentation of current therapy  Comparator: Saline |
| Lapidus 2014 [7]  Study design: crossover | Sample size: 20  Diagnosis: Unipolar Depression  Treatment resistant: Included  Suicidal ideation: Excluded | Participants were required to have failed to respond to at least one trial of adequate dose and duration of an antidepressant medication approved by the U.S. Food and Drug Administration in the current episode according to the Antidepressant Treatment History Form | No high risk of suicide | Intervention: Ketamine IN  Administration strategy: Augmentation of current therapy  Comparator: Saline |
| Li 2016 [8]  Study design: parallel | Sample size: 48  Diagnosis: Unipolar Depression  Treatment resistant: Included  Suicidal ideation: Excluded | All TRD patients were required to have a history of failing to respond to at least three different antidepressants with adequate dosage and treatment duration and of failing at least one trial of adequate antidepressant treatment during their current depressive episode. | No active suicide attempts in a hospital setting or homicide risks. | Intervention: Ketamine IV  Administration strategy: Augmentation of current therapy  Comparator: Saline |
| Murrough 2013 [9]  Study design: parallel | Sample size: 73  Diagnosis: Unipolar Depression  Treatment resistant: Included  Suicidal ideation: Excluded | Inadequate response to at least three therapeutic trials of an antidepressant according to the criteria of the Antidepressant Treatment History Form | No serious and imminent suicidal or homicidal risk | Intervention: Ketamine IV  Administration strategy: Monotherapy  Comparator: Midazolam |
| Ochs-Ross 2019 [10]  Study design: parallel | Sample size: 138  Diagnosis: Unipolar Depression  Treatment resistant: Included  Suicidal ideation: Excluded | No clinically meaningful improvement following treatment with ≥2 different antidepressant agents, prescribed in adequate dosages for adequate duration, during the current episode of depression.  Documented nonresponse to ≥1 but ≤8 oral antidepressant treatments taken at adequate dosage and for an adequate duration, within the current episode of depression (as assessed on the geriatric version of the MGH-ATRQ41 and confirmed by documented records) at the start of the 4-week screening/prospective observational phase.  Nonresponse to ≥1 one of the 2 failed antidepressant medications was confirmed prospectively during the screening phase of the study. All patients had to have been nonresponsive to at least 2 oral antidepressants prior to randomization | No suicidal ideation with intent to act within 6 months | Intervention: Esketamine IN  Administration strategy: Augmentation of new therapy  Comparator: Saline |
| Popova 2019 [11]  Study design: parallel | Sample size: 223  Diagnosis: Unipolar Depression  Treatment resistant: Included  Suicidal ideation: Excluded | Nonresponse to an adequate trial (dosage, duration, and adherence) of at least two antidepressants in the current episode (of which one was observed prospectively). | No current or recent (past 6 months) homicidal ideation/intent or suicidal ideation with intent to act or suicidal behavior within the past year | Intervention: Esketamine IN  Administration strategy: Augmentation of new therapy  Comparator: Saline |
| Singh 2016b [12]  Study design: parallel multiarm (0.2mg/kg, 0.4mg/kg) | Sample size: 19  Diagnosis: Unipolar Depression  Treatment resistant: Included  Suicidal ideation: Excluded | Patients were required to have had an inadequate response to at least one antidepressant drug in their current depressive episode and an inadequate response to at least one other antidepressant either in their current or in a previous depressive episode, as assessed by the Massachusetts General Hospital Antidepressant Treatment Response Questionnaire | No suicidal or homicidal requiring hospitalization in the past 12 months | Intervention: Esketamine IV  Administration strategy: Augmentation of current therapy  Comparator: Saline |
| Singh  2016a [13]  Study design: parallel multiarm (2 times a week, 3 times a week) | Sample size: 33  Diagnosis: Unipolar Depression  Treatment resistant: Included  Suicidal ideation: Excluded | Qualifying valid depressive episodes, as assessed with the SAFER criteria (defined as state versus trait, assessability, face validity, ecological validity, and rule of three Ps—pervasive, persistent, and pathological); inadequate response to at least two antidepressants (with at least one antidepressant failure in the current episode), assessed by medication history and the Massachusetts General Hospital Antidepressant Treatment Response Questionnaire; and a score >34 on the 30-item Inventory of Depressive Symptomatology–Clinician Rated at screening and pr infusion assessment on day 1. Independent SAFER raters from Massachusetts General Hospital verified that all randomized patients met the SAFER criteria, had treatment-resistant depression documented on the Antidepressant Treatment Response Questionnaire, and manifested the required depression severity. | No clinically significant suicidal or homicidal ideation (imminent risk of harm) | Intervention: Ketamine IV  Administration strategy: Augmentation of current therapy  Comparator: Saline |
| Takahashi 2021 [14]  Study design: parallel multiarm | Sample size: 202  Diagnosis: Unipolar Depression  Treatment resistant: Included  Suicidal ideation: Excluded | During the prospective lead-in phase, patients received a new OL oral AD therapy; non-responders to this oral AD (defined as those who achieved ≤ 25% improvement in the Montgomery-Asberg Depression Rating Scale [MADRS] total score during the prospective lead-in phase and at randomization) and patients with a MADRS total score of ≥ 28 at 2 weeks before randomization and at randomization were considered to meet the definition of TRD. Also, non-responders (≤25% improvement in the MADRS total score) to ≥ 1 but < 5 different oral ADs (based on Massachusetts General Hospital- Antidepressant Treatment Response Questionnaire [MGH-ATRQ]) in the current episode were enrolled. | No suicidal ideation with some intent to act within 6 months of screening or those with history of suicidal behavior within the past year | Intervention: Esketamine IN  3 dosages: 28mg, 56mg, 84mg  Administration strategy: Augmentation of current therapy  Comparator: Saline |
| Zarate 2006 [15]  Study design: crossover | Sample size: 18  Diagnosis: Unipolar Depression  Treatment resistant: Included  Suicidal ideation: Excluded | All subjects but 1 had failed an adequate antidepressant trial for the current major depressive episode. They were also required to have previously failed at least 2 adequate antidepressant trials (adequacy of antidepressant trials was determined with the Antidepressant Treatment History Form). | Judged clinically not to be a serious suicide risk | Intervention: Ketamine IV  Administration strategy: Monotherapy  Comparator: Saline |
| **Treatment resistant, no details on suicidal ideation** | | | | |
| Cao 2018 [16]  Study design: parallel | Sample size: 55  Diagnosis: Unipolar Depression  Treatment resistant: Included  Suicidal ideation: Not reported | Patients with poor treatment history with at least two different antidepressants (with sufficient dosages and treatment periods) and at least one invalid effort with adequate antidepressant treatment during their current depressive episode to ensure that each subject was resistant to antidepressant medications | No information |  |
| Chen 2018 [17]  Study design: parallel | Sample size: 24  Diagnosis: Unipolar Depression  Treatment resistant: Included  Suicidal ideation: Not reported | Who had failed to respond to at least three different antidepressants with adequate dosage and treatment duration were enrolled in our current study | No information | Intervention: Ketamine IV  Administration strategy: Augmentation of current therapy  Comparator: Saline |
| Domany 2018 [18]  Study design: parallel | Sample size: 41  Diagnosis: Unipolar Depression  Treatment resistant: Included  Suicidal ideation: Not reported | Inadequate response to documented therapeutic trials of at least two antidepressants of an adequate dose and duration. | No information | Intervention: Ketamine Oral  Administration strategy: Augmentation of current therapy  Comparator: Saline |
| Ekstrand 2022 [19]  Study design: parallel | Sample size: 199  Diagnosis: Unipolar Depression  Treatment resistant: Included  Suicidal ideation: Not reported | No information | No information | Intervention: Ketamine IV  Administration strategy: Augmentation of current therapy  Comparator: ECT |
| Fava 2018 [20]  Study design: parallel | Sample size: 99  Diagnosis: Unipolar Depression  Treatment resistant: Included  Suicidal ideation: Not reported | Failure to achieve a subjective satisfactory response (e.g., less than 50% improvement of depression symptoms) to at least two adequate treatment courses during the current depressive episode (including the current ADT) | No information | Intervention: Ketamine IV  Administration strategy: Augmentation of current therapy  Comparator: Midazolam |
| Ghasemi 2014 [21]  Study design: parallel | Sample size: 18  Diagnosis: Unipolar Depression  Treatment resistant: Included  Suicidal ideation: Not reported | Assumed from ECT eligibility of the patients | No information | Intervention: Ketamine IV  Administration strategy: Not reported  Comparator: ECT |
| Kheirabadi 2019 [22]  Study design: parallel multiarm | Sample size: 32  Diagnosis: Unipolar Depression  Treatment resistant: Included  Suicidal ideation: Not reported | Assumed from ECT eligibility of the patients | No information | Interventions: Ketamine IV, Ketamine oral, Ketamine IM  Administration strategy: Not reported  Comparator: ECT |
| Kheirabadi 2020 [23]  Study design: parallel multiarm (oral, IM) | Sample size: 39  Diagnosis: Unipolar Depression  Treatment resistant: Included  Suicidal ideation: Not reported | Assumed from ECT eligibility of the patients | No information | Interventions: Ketamine Oral, Ketamine IM  Administration strategy: Augmentation of current therapy  Comparator: ECT |
| Lai 2014 [24]  Study design: crossover | Sample size: 4  Diagnosis: Unipolar Depression  Treatment resistant: Included  Suicidal ideation: Not reported | Subjects were required to have a Montgomery–Asberg Depression Rating Scale (MADRS; Montgomery and Asberg, 1979) score > 20 and to have had insufficient therapeutic response to at least one adequate trial of an antidepressant medication during the current illness episode. Treatment resistance was measured using the Maudsley Staging of treatment Resistance | No information | Intervention: Ketamine IV  Administration strategy: Augmentation of current therapy  Comparator: Saline |
| NCT02918318 2020[25]  Study design: parallel | Sample size: 202  Diagnosis: Unipolar Depression  Treatment resistant: Included  Suicidal ideation: Not reported | At the start of the prospective lead-in phase, subjects must not have responded to >1 but <5 different oral antidepressants taken at adequate dosage and for adequate duration, as assessed on the MGH-ATRQ and documented by medical history/prescription records, for the current episode of depression. Additionally, in the prospective lead-in phase, all subjects will start a new switched antidepressant to confirm nonresponse to ≥2 antidepressants in their current episode. | No information | Intervention: Esketamine IN  Administration strategy: Augmentation of current therapy  Comparator: Saline |
| Phillips 2019 [26]  Study design: crossover | Sample size: 43  Diagnosis: Unipolar Depression  Treatment resistant: Included  Suicidal ideation: Not reported | Failure to respond to at least two antidepressant medications of different pharmacological classes and two augmentation strategies at adequate dosages for at least 6 weeks each during the present episode, as recorded in the Antidepressant Treatment History Form (20). | No information | Intervention: Ketamine IV  Administration strategy: Augmentation of current therapy  Comparator: Midazolam |
| Price 2014 [27]  Study design: parallel | Sample size: 57  Diagnosis: Unipolar Depression  Treatment resistant: Included  Suicidal ideation: Not reported | Three or more failed, adequate antidepressant trials, as determined by the Antidepressant Treatment History Form. [ | Suicide measures were not collected in all participants due to  unavailability of equipment/time constraints (n = 15) or patient refusal (n = 3). The Beck Scale for Suicide Ideation (BSS), [28] a 21-item self-report measure that is reliable and highly correlated (r> = .90) with the clinician-rated Scale for Suicide Ideation-->Come valutano | Intervention: Ketamine IV  Administration strategy: Monotherapy  Comparator: Midazolam |
| Sharma 2020 [28]  Study design: parallel | Sample size: 25  Diagnosis: Unipolar Depression, Bipolar Depression  Treatment resistant: Included  Suicidal ideation: Not reported | No information | No information | Intervention: Ketamine IV  Administration strategy: Augmentation of current therapy  Comparator: ECT |
| Su 2017 [29]  Study design: parallel multiarm (0.2mg/kg, 0.5mg/kg) | Sample size: 71  Diagnosis: Unipolar Depression  Treatment resistant: Included  Suicidal ideation: Not reported | Patients had failed to respond to more than two adequate antidepressant trials. | No information | Intervention: Ketamine IV  2 dosages: 0.2mg/kg, 0.5mg/kg  Administration strategy: Augmentation of current therapy  Comparator: Saline |
| Zarate 2012 [30]  Study design: crossover | Sample size: 15  Diagnosis: Bipolar Depression  Treatment resistant: Included  Suicidal ideation: Not reported | Subjects were required to have a MADRS score of 20 at screening and at the start of each ketamine/placebo infusion. Patients were also required to have previously failed at least one adequate antidepressant trial (as assessed by the Antidepressant Treatment History Form-modified) and to have failed a prospective open trial of a mood stabilizer while at the NIMH (lithium or valproate for at least 4 weeks at therapeutic levels [serum lithium .6 –1.2 mEq/L or valproic acid 50 –125 g/mL]). | No information | Intervention: Ketamine IV  Administration strategy: Augmentation of current therapy  Comparator: Saline |
| **Non treatment resistant, with suicidal ideation** | | | | |
| Sinyor 2018 [31]  Study design: parallel | Sample size: 9  Diagnosis: Unipolar Depression  Treatment resistant: Excluded  Suicidal ideation: Included | MDD did not have to be treatment-resistant (defined as ≥ 2 failed lifetime antidepressant trials of adequate dose and duration). | Defined by a score of > 0 on either of the Scale of Suicidal Ideation (SSI, clinician administered; Beck et al., 1979) and the Columbia-Suicide Severity Rating Scale (CSSRS) or both | Intervention: Ketamine IV  Administration strategy: Augmentation of current therapy  Comparator: None |
| **No details on treatment resistance, with suicidal ideation** | | | | |
| Canuso 2018 [32]  Study design: parallel | Sample size: 68  Diagnosis: Unipolar Depression  Treatment resistant: Not reported  Suicidal ideation: Included | No information | Participants, to be included, responded affirmatively to MINI questions B5 (“Think about suicide [killing yourself]?”) in the present and B9 (“Intend to act on thoughts of killing yourself?”) in the past 24 hours, that they be in clinical need of acute psychiatric hospitalization due to imminent risk for suicide, and that they have a score ≥22 on the Montgomery-Åsberg Depression Rating Scale (MADRS) on day 1 before dosing | Intervention: Esketamine IN  Administration strategy: Augmentation of current therapy  Comparator: Saline |
| Fu 2020 [33]  Study design: parallel | Sample size: 226  Diagnosis: Unipolar Depression  Treatment resistant: Not reported  Suicidal ideation: Included | No information | Patients respond affirmatively to MINI questions B3 (“Think about suicide [killing yourself]?”) and B10 (“Intend to act on thoughts of killing yourself in the past 24 hours?”) within 24 hours of randomization, be in clinical need of acute psychiatric hospitalization due to imminent suicide risk | Intervention: Esketamine IN  Administration strategy: Augmentation of new therapy  Comparator: Saline |
| Grunebaum 2017 [34]  Study design: parallel | Sample size: 16  Diagnosis: Bipolar Depression  Treatment resistant: Not reported  Suicidal ideation: Included | No information | A score of ≥4 on the Scale for Suicidal Ideation (SSI) | Intervention: Ketamine IV  Administration strategy: Augmentation of current therapy  Comparator: Midazolam |
| Grunebaum 2018 [35]  Study design: parallel | Sample size: 80  Diagnosis: Unipolar Depression  Treatment resistant: Not reported  Suicidal ideation: Included | No information | Score equal or more than. 4 on the SSI | Intervention: Ketamine IV  Administration strategy: Augmentation of current therapy, Monotherapy  Comparator: Midazolam |
| Ionescu 2021 [36]  Study design: parallel | Sample size: 230  Diagnosis: Unipolar Depression  Treatment resistant: Not reported  Suicidal ideation: Included | No information | Enrollees were also required to present with suicidal ideation with intent based on affirmative responses to Mini International Neuropsychiatric Interview (for DSM-5) questions B3 (Currently think about harming/hurting/injuring yourself or about suicide?) and B10 (Intend to act on thoughts of killing yourself?) assessed within 24 hours of randomization | Intervention: Esketamine IN  Administration strategy: Augmentation of new therapy  Comparator: |
| Murrough 2015 [37]  Study design: parallel | Sample size: 24  Diagnosis: Unipolar Depression, Bipolar Depression  Treatment resistant: Not reported  Suicidal ideation: Included | No information | Current clinically significant suicidal ideation, operationalized as a score of 54 on the suicide item of the Montgomery–Asberg Depression Rating Scale (MADRS-SI; range 0–6) at the time of screening. Outpatients were excluded if they experienced current intent to make a suicide attempt as reflected by a SI score on the Columbia Suicide Severity Scale (C-SSRS; Posner et al. 2007) of 4 or 5. | Intervention: Ketamine IV  Administration strategy: Augmentation of current therapy  Comparator: Midazolam |
| **No details on treatment resistance, without suicidal ideation** | | | | |
| Hu 2016 [38]  Study design: parallel | Sample size: 30  Diagnosis: Unipolar Depression  Treatment resistant: Not reported  Suicidal ideation: Excluded | No information | No information | Intervention: Ketamine IV  Administration strategy: Augmentation of new therapy  Comparator: Saline |
| Jafarinia 2016 [39]  Study design: parallel | Sample size: 46  Diagnosis: Unipolar Depression  Treatment resistant: Not reported  Suicidal ideation: Excluded | No information | Those who were judged to have substantial risk for suicide by the physician or score >2 on the suicide item of the HDRS | Intervention: Ketamine Oral  Administration strategy: Monotherapy  Comparator: Diclofenac |
| Sos 2013 [40]  Study design: crossover | Sample size: 30  Diagnosis: Unipolar Depression  Treatment resistant: Not reported  Suicidal ideation: Excluded | No information | Any suicidal risk assessed by clinical examination | Intervention: Ketamine IV  Administration strategy: Augmentation of current therapy  Comparator: Saline |
| **No details on treatment resistance, no details on suicidal ideation** | | | | |
| Arabzadeh 2018 [41]  Study design: parallel | Sample size: 90  Diagnosis: Unipolar Depression  Treatment resistant: Not reported  Suicidal ideation: Not reported | No information | No information | Intervention: Ketamine Oral  Administration strategy: Augmentation of current therapy  Comparator: Saline |
| Berman 2000 [42]  Study design: parallel | Sample size: 9  Diagnosis: Unipolar Depression  Treatment resistant: Not reported  Suicidal ideation: Not reported | No information | No information | Intervention: Ketamine IV  Administration strategy: Monotherapy  Comparator: Saline |
| Downey 2016a [43]  Study design: parallel | Sample size: 40  Diagnosis: Unipolar Depression  Treatment resistant: Not reported  Suicidal ideation: Not reported | No information | No information | Intervention: Ketamine IV  Administration strategy: Monotherapy  Comparator: Saline |
| Downey 2016b [43]  Study design: parallel | Sample size: 41  Diagnosis: Unipolar Depression  Treatment resistant: Not reported  Suicidal ideation: Not reported | No information | No information | Intervention: Ketamine IV  Administration strategy: Monotherapy  Comparator: Lanicemine |
| Kudoh 2002 [44]  Study design: parallel | Sample size: 70  Diagnosis: Unipolar Depression  Treatment resistant: Not reported  Suicidal ideation: Not reported | No information | No information | Intervention: Ketamine IV, Propofol, Fentanyl  Administration strategy: Augmentation of current therapy  Comparator: Propofol, Fentanyl |

References

1. Ionescu, D.F., et al., *Repeat-dose ketamine augmentation for treatment-resistant depression with chronic suicidal ideation: A randomized, double blind, placebo controlled trial.* J Affect Disord, 2019. **243**: p. 516-524.

2. Daly, E.J., et al., *Efficacy and Safety of Intranasal Esketamine Adjunctive to Oral Antidepressant Therapy in Treatment-Resistant Depression: A Randomized Clinical Trial.* JAMA Psychiatry, 2018. **75**(2): p. 139-148.

3. Diazgranados, N., et al., *A randomized add-on trial of an N-methyl-D-aspartate antagonist in treatment-resistant bipolar depression.* Arch Gen Psychiatry, 2010. **67**(8): p. 793-802.

4. Fedgchin, M., et al., *Efficacy and Safety of Fixed-Dose Esketamine Nasal Spray Combined With a New Oral Antidepressant in Treatment-Resistant Depression: Results of a Randomized, Double-Blind, Active-Controlled Study (TRANSFORM-1).* Int J Neuropsychopharmacol, 2019. **22**(10): p. 616-630.

5. Galvez, V., et al., *Repeated intranasal ketamine for treatment-resistant depression - the way to go? Results from a pilot randomised controlled trial.* J Psychopharmacol, 2018. **32**(4): p. 397-407.

6. Lally, N., et al., *Anti-anhedonic effect of ketamine and its neural correlates in treatment-resistant bipolar depression.* Transl Psychiatry, 2014. **4**(10): p. e469.

7. Lapidus, K.A., et al., *A randomized controlled trial of intranasal ketamine in major depressive disorder.* Biol Psychiatry, 2014. **76**(12): p. 970-6.

8. Li, D.J., et al., *Significant treatment effect of add-on ketamine anesthesia in electroconvulsive therapy in depressive patients: A meta-analysis.* Eur Neuropsychopharmacol, 2017. **27**(1): p. 29-41.

9. Murrough, J.W., et al., *Antidepressant efficacy of ketamine in treatment-resistant major depression: a two-site randomized controlled trial.* Am J Psychiatry, 2013. **170**(10): p. 1134-42.

10. Ochs-Ross, R., et al., *Efficacy and Safety of Esketamine Nasal Spray Plus an Oral Antidepressant in Elderly Patients With Treatment-Resistant Depression-TRANSFORM-3.* Am J Geriatr Psychiatry, 2020. **28**(2): p. 121-141.

11. Popova, V., et al., *Efficacy and Safety of Flexibly Dosed Esketamine Nasal Spray Combined With a Newly Initiated Oral Antidepressant in Treatment-Resistant Depression: A Randomized Double-Blind Active-Controlled Study.* Am J Psychiatry, 2019. **176**(6): p. 428-438.

12. Singh, J.B., et al., *Intravenous Esketamine in Adult Treatment-Resistant Depression: A Double-Blind, Double-Randomization, Placebo-Controlled Study.* Biol Psychiatry, 2016. **80**(6): p. 424-431.

13. Singh, J.B., et al., *A Double-Blind, Randomized, Placebo-Controlled, Dose-Frequency Study of Intravenous Ketamine in Patients With Treatment-Resistant Depression.* Am J Psychiatry, 2016. **173**(8): p. 816-26.

14. Takahashi, N., et al., *Efficacy and safety of fixed doses of intranasal Esketamine as an add-on therapy to Oral antidepressants in Japanese patients with treatment-resistant depression: a phase 2b randomized clinical study.* BMC Psychiatry, 2021. **21**(1): p. 526.

15. Zarate, C.A., Jr., et al., *A randomized trial of an N-methyl-D-aspartate antagonist in treatment-resistant major depression.* Arch Gen Psychiatry, 2006. **63**(8): p. 856-64.

16. Zehong, C., et al., *Identifying Ketamine Responses in Treatment-Resistant Depression Using a Wearable Forehead EEG.* IEEE Trans Biomed Eng, 2019. **66**(6): p. 1668-1679.

17. Chen, M.H., et al., *Persistent antidepressant effect of low-dose ketamine and activation in the supplementary motor area and anterior cingulate cortex in treatment-resistant depression: A randomized control study.* J Affect Disord, 2018. **225**: p. 709-714.

18. Domany, Y., et al., *Repeated oral ketamine for out-patient treatment of resistant depression: randomised, double-blind, placebo-controlled, proof-of-concept study.* Br J Psychiatry, 2019. **214**(1): p. 20-26.

19. Ekstrand, J., et al., *Racemic Ketamine as an Alternative to Electroconvulsive Therapy for Unipolar Depression: A Randomized, Open-Label, Non-Inferiority Trial (KetECT).* Int J Neuropsychopharmacol, 2022. **25**(5): p. 339-349.

20. Fava, M., et al., *Double-blind, placebo-controlled, dose-ranging trial of intravenous ketamine as adjunctive therapy in treatment-resistant depression (TRD).* Mol Psychiatry, 2020. **25**(7): p. 1592-1603.

21. Ghasemi, M., et al., *Rapid antidepressant effects of repeated doses of ketamine compared with electroconvulsive therapy in hospitalized patients with major depressive disorder.* Psychiatry Res, 2014. **215**(2): p. 355-61.

22. Kheirabadi, G., et al., *Comparative Effect of Intravenous Ketamine and Electroconvulsive Therapy in Major Depression: A Randomized Controlled Trial.* Adv Biomed Res, 2019. **8**: p. 25.

23. Kheirabadi, D., et al., *Comparison of Rapid Antidepressant and Antisuicidal Effects of Intramuscular Ketamine, Oral Ketamine, and Electroconvulsive Therapy in Patients With Major Depressive Disorder: A Pilot Study.* J Clin Psychopharmacol, 2020. **40**(6): p. 588-593.

24. Lai, R., et al., *Pilot dose-response trial of i.v. ketamine in treatment-resistant depression.* World J Biol Psychiatry, 2014. **15**(7): p. 579-84.

25. NCT02918318, *A Study to Evaluate the Efficacy, Safety and Tolerability of Fixed Doses of Intranasal Esketamine in Japanese Participants With Treatment Resistant Depression*. 2016, <https://ClinicalTrials.gov/show/NCT02918318>.

26. Phillips, J.L., et al., *Single, Repeated, and Maintenance Ketamine Infusions for Treatment-Resistant Depression: A Randomized Controlled Trial.* Am J Psychiatry, 2019. **176**(5): p. 401-409.

27. Price, R.B., et al., *Effects of ketamine on explicit and implicit suicidal cognition: a randomized controlled trial in treatment-resistant depression.* Depress Anxiety, 2014. **31**(4): p. 335-43.

28. Sharma, R.K., et al., *Antidepressant effects of ketamine and ECT: A pilot comparison.* J Affect Disord, 2020. **276**: p. 260-266.

29. Su, T.P., et al., *Dose-Related Effects of Adjunctive Ketamine in Taiwanese Patients with Treatment-Resistant Depression.* Neuropsychopharmacology, 2017. **42**(13): p. 2482-2492.

30. Zarate, C.A., Jr., et al., *Replication of ketamine's antidepressant efficacy in bipolar depression: a randomized controlled add-on trial.* Biol Psychiatry, 2012. **71**(11): p. 939-46.

31. Sinyor, M., et al., *Ketamine augmentation for major depressive disorder and suicidal ideation: Preliminary experience in an inpatient psychiatry setting.* J Affect Disord, 2018. **241**: p. 103-109.

32. Canuso, C.M., et al., *Efficacy and Safety of Intranasal Esketamine for the Rapid Reduction of Symptoms of Depression and Suicidality in Patients at Imminent Risk for Suicide: Results of a Double-Blind, Randomized, Placebo-Controlled Study.* Am J Psychiatry, 2018. **175**(7): p. 620-630.

33. Fu, D.J., et al., *Esketamine Nasal Spray for Rapid Reduction of Major Depressive Disorder Symptoms in Patients Who Have Active Suicidal Ideation With Intent: Double-Blind, Randomized Study (ASPIRE I).* J Clin Psychiatry, 2020. **81**(3).

34. Grunebaum, M.F., et al., *Ketamine versus midazolam in bipolar depression with suicidal thoughts: A pilot midazolam-controlled randomized clinical trial.* Bipolar Disord, 2017. **19**(3): p. 176-183.

35. Grunebaum, M.F., et al., *Ketamine for Rapid Reduction of Suicidal Thoughts in Major Depression: A Midazolam-Controlled Randomized Clinical Trial.* Am J Psychiatry, 2018. **175**(4): p. 327-335.

36. Ionescu, D.F., et al., *Esketamine Nasal Spray for Rapid Reduction of Depressive Symptoms in Patients With Major Depressive Disorder Who Have Active Suicide Ideation With Intent: Results of a Phase 3, Double-Blind, Randomized Study (ASPIRE II).* Int J Neuropsychopharmacol, 2021. **24**(1): p. 22-31.

37. Murrough, J.W., et al., *Ketamine for rapid reduction of suicidal ideation: a randomized controlled trial.* Psychological Medicine, 2015. **45**(16): p. 3571-3580.

38. Hu, Y.D., et al., *Single i.v. ketamine augmentation of newly initiated escitalopram for major depression: results from a randomized, placebo-controlled 4-week study.* Psychol Med, 2016. **46**(3): p. 623-35.

39. Jafarinia, M., et al., *Efficacy and safety of oral ketamine versus diclofenac to alleviate mild to moderate depression in chronic pain patients: A double-blind, randomized, controlled trial.* J Affect Disord, 2016. **204**: p. 1-8.

40. Sos, P., et al., *Relationship of ketamine's antidepressant and psychotomimetic effects in unipolar depression.* Neuro Endocrinol Lett, 2013. **34**(4): p. 287-93.

41. Arabzadeh, S., et al., *Does oral administration of ketamine accelerate response to treatment in major depressive disorder? Results of a double-blind controlled trial.* J Affect Disord, 2018. **235**: p. 236-241.

42. Berman, R.M., et al., *Antidepressant effects of ketamine in depressed patients.* Biol Psychiatry, 2000. **47**(4): p. 351-4.

43. Downey, D., et al., *Comparing the actions of lanicemine and ketamine in depression: key role of the anterior cingulate.* Eur Neuropsychopharmacol, 2016. **26**(6): p. 994-1003.

44. Kudoh, A., et al., *Small-dose ketamine improves the postoperative state of depressed patients.* Anesth Analg, 2002. **95**(1): p. 114-8, table of contents.

## Supplementary Table 4. Table of the wrongly included studies

| **Study name** | **Reason for exclusion** | **Reviews that included these studies** |
| --- | --- | --- |
| aan het Rot 2010 [1] | Design: no control arm | Marcantoni 2020 |
| Anderson 2017 [2] | Intervention: ketamine as electroconvulsive therapy augmentation | Witt 2020 |
| Ballard 2014 [3] | Design: no control arm | Wilkinson 2018 |
| Basso 2020 [4] | Design: no control arm | Rhee 2022 |
| Burger 2016 [5] | Participants: no diagnosis of affective disorder, only suicidal ideation | Xiong 2021 |
| Correia-Melo 2020 [6] | Comparator: compare ketamine and esketamine | Bahji 2021 |
| Fan 2016 [7] | Participants: no diagnosis of affective disorder, only suicidal ideation | McIntyre 2020 |
| Feder 2014 [8] | Participants: no diagnosis of affective disorder, only suicidal ideation | Wilkinson 2018 |
| George 2017 [9] | Design: crossover not correctly done | Witt 2020, Bahji 2022 |
| Ibrahim 2012 [10] | Comparator: ketamine + riluzole vs. ketamine + placebo | McIntyre 2020 |
| Jarventausta 2013 [11] | Intervention: ketamine as electroconvulsive therapy augmentation | Caddy 2015 |
| Loo 2012 [12] | Intervention: ketamine as electroconvulsive therapy augmentation | Caddy 2015 |
| Loo 2016 [13] | Design: no control arm | Xu 2016, Witt 2020, Bahji 2022 |
| Nugent 2019 [14] | Design: duplicate of Zarate 2006 [15] | Kryst 2020, Bahji 2022 |
| Ray-Griffith 2017 [16] | Intervention: ketamine as electroconvulsive therapy augmentation | Witt 2020 |
| Valentine 2011 [17] | Design: no control arm | Caddy 2015, Fond 2014, Wilkinson 2018 |
| Yoosefi 2014 [18] | Intervention: ketamine as electroconvulsive therapy augmentation | Caddy 2015 |

References

[1] aan het Rot M, Collins KA, Murrough JW, Perez AM, Reich DL, Charney DS, et al. Safety and efficacy of repeated-dose intravenous ketamine for treatment-resistant depression. Biol Psychiatry. 2010;67(2):139-45. <https://doi.org/10.1016/j.biopsych.2009.08.038>

[2] Anderson IM, Blamire A, Branton T, Clark R, Downey D, Dunn G, et al. Ketamine augmentation of electroconvulsive therapy to improve neuropsychological and clinical outcomes in depression (Ketamine-ECT): a multicentre, double-blind, randomised, parallel-group, superiority trial. Lancet Psychiatry. 2017;4(5):365-77. <https://doi.org/10.1016/S2215-0366(17)30077-9>

[3] Ballard ED, Ionescu DF, Vande Voort JL, Niciu MJ, Richards EM, Luckenbaugh DA, et al. Improvement in suicidal ideation after ketamine infusion: relationship to reductions in depression and anxiety. Journal of psychiatric research. 2014;58:161-6. <https://doi.org/10.1016/j.jpsychires.2014.07.027>

[4] Basso L, Bonke L, Aust S, Gartner M, Heuser-Collier I, Otte C, et al. Antidepressant and neurocognitive effects of serial ketamine administration versus ECT in depressed patients. Journal of psychiatric research. 2020;123:1-8. <https://doi.org/10.1016/j.jpsychires.2020.01.002>

[5] Burger J, Capobianco M, Lovern R, Boche B, Ross E, Darracq MA, et al. A Double-Blinded, Randomized, Placebo-Controlled Sub-Dissociative Dose Ketamine Pilot Study in the Treatment of Acute Depression and Suicidality in a Military Emergency Department Setting. Mil Med. 2016;181(10):1195-9. <https://doi.org/10.7205/MILMED-D-15-00431>

[6] Correia-Melo FS, Leal GC, Vieira F, Jesus-Nunes AP, Mello RP, Magnavita G, et al. Efficacy and safety of adjunctive therapy using esketamine or racemic ketamine for adult treatment-resistant depression: A randomized, double-blind, non-inferiority study. J Affect Disord. 2020;264:527-34. <https://doi.org/10.1016/j.jad.2019.11.086>

[7] Fan W, Yang H, Sun Y, Zhang J, Li G, Zheng Y, et al. Ketamine rapidly relieves acute suicidal ideation in cancer patients: a randomized controlled clinical trial. Oncotarget. 2017;8(2):2356-60. <https://doi.org/10.18632/oncotarget.13743>

[8] Feder A, Parides MK, Murrough JW, Perez AM, Morgan JE, Saxena S, et al. Efficacy of intravenous ketamine for treatment of chronic posttraumatic stress disorder: a randomized clinical trial. JAMA Psychiatry. 2014;71(6):681-8. <https://doi.org/10.1001/jamapsychiatry.2014.62>

[9] George D, Galvez V, Martin D, Kumar D, Leyden J, Hadzi-Pavlovic D, et al. Pilot Randomized Controlled Trial of Titrated Subcutaneous Ketamine in Older Patients with Treatment-Resistant Depression. Am J Geriatr Psychiatry. 2017;25(11):1199-209. <https://doi.org/10.1016/j.jagp.2017.06.007>

[10] Ibrahim L, Diazgranados N, Franco-Chaves J, Brutsche N, Henter ID, Kronstein P, et al. Course of improvement in depressive symptoms to a single intravenous infusion of ketamine vs add-on riluzole: results from a 4-week, double-blind, placebo-controlled study. Neuropsychopharmacology. 2012;37(6):1526-33. <https://doi.org/10.1038/npp.2011.338>

[11] Jarventausta K, Chrapek W, Kampman O, Tuohimaa K, Bjorkqvist M, Hakkinen H, et al. Effects of S-ketamine as an anesthetic adjuvant to propofol on treatment response to electroconvulsive therapy in treatment-resistant depression: a randomized pilot study. J ECT. 2013;29(3):158-61. <https://doi.org/10.1097/YCT.0b013e318283b7e9>

[12] Loo CK, Katalinic N, Garfield JB, Sainsbury K, Hadzi-Pavlovic D, Mac-Pherson R. Neuropsychological and mood effects of ketamine in electroconvulsive therapy: a randomised controlled trial. J Affect Disord. 2012;142(1-3):233-40. <https://doi.org/10.1016/j.jad.2012.04.032>

[13] Loo CK, Galvez V, O'Keefe E, Mitchell PB, Hadzi-Pavlovic D, Leyden J, et al. Placebo-controlled pilot trial testing dose titration and intravenous, intramuscular and subcutaneous routes for ketamine in depression. Acta Psychiatr Scand. 2016;134(1):48-56. <https://doi.org/10.1111/acps.12572>

[14] Nugent AC, Ballard ED, Gould TD, Park LT, Moaddel R, Brutsche NE, et al. Ketamine has distinct electrophysiological and behavioral effects in depressed and healthy subjects. Mol Psychiatry. 2019;24(7):1040-52. <https://doi.org/10.1038/s41380-018-0028-2>

[15] Zarate CA, Jr., Singh JB, Carlson PJ, Brutsche NE, Ameli R, Luckenbaugh DA, et al. A randomized trial of an N-methyl-D-aspartate antagonist in treatment-resistant major depression. Arch Gen Psychiatry. 2006;63(8):856-64. <https://doi.org/10.1001/archpsyc.63.8.856>

[16] Ray-Griffith SL, Eads LA, Han X, Golden K, Stowe ZN. A Randomized Pilot Study Comparing Ketamine and Methohexital Anesthesia for Electroconvulsive Therapy in Patients With Depression. J ECT. 2017;33(4):268-71. <https://doi.org/10.1097/YCT.0000000000000413>

[17] Valentine GW, Mason GF, Gomez R, Fasula M, Watzl J, Pittman B, et al. The antidepressant effect of ketamine is not associated with changes in occipital amino acid neurotransmitter content as measured by [(1)H]-MRS. Psychiatry Res. 2011;191(2):122-7. <https://doi.org/10.1016/j.pscychresns.2010.10.009>

[18] Yoosefi A, Sepehri AS, Kargar M, Akhondzadeh S, Sadeghi M, Rafei A, et al. Comparing effects of ketamine and thiopental administration during electroconvulsive therapy in patients with major depressive disorder: a randomized, double-blind study. J ECT. 2014;30(1):15-21. <https://doi.org/10.1097/YCT.0b013e3182a4b4c6>

## Supplementary Table 5. Extended outcomes table

| **Study name** | | **Population** | **Intervention** | **Comparator** | **Statistical model** | **Effect size type** | **Statistical Data** | **Heterogeneity (I², p-value) and Publication bias** |
| --- | --- | --- | --- | --- | --- | --- | --- | --- |
| **Acceptability – Lack of efficacy**  **(Endpoint)** | | | | | | | | |
| Dean 2021 | | Diagnosis: Bipolar Depression  Resistant: Yes  Suicidal: Not reported in some of the RCTs | Ketamine IV | Saline | Random Effect | OR | Sample size: 33  Effect measure: 5.65 (0.76, 41.87)  p-value: 0.09 | Het.: I²: 0%, p=0.93  Pub.B. Test: NA, p=NA |
| Zheng 2020 | | Diagnosis: Unipolar Depression  Resistant: No Information  Suicidal: No information | Esketamine IN | Saline | Random Effect | RR | Sample size: 133  Effect measure: 1.75 (0.41, 7.52)  p-value: 0.45 | Het.: I²: 22%, p=NA  Pub.B. Test: NA, p=NA |
| **Acceptability – Total Dropouts**  **(>28d)** | | | | | | | | |
| Fornaro 2020 | | Diagnosis: Bipolar Depression  Resistant: Yes  Suicidal: Not reported in some of the RCTs | Ketamine IV | Saline | Random Effect | OR | Sample size: 33  Effect measure: 4.149 (0.79, 21.787)  p-value: 0.093 | Het.: I²: 0%, p=0.093  Pub.B. Test: Egger, Begg-Mazumdar Kendall's tau, p=NA |
| **Acceptability – Total droupouts**  **(Endpoint)** | | | | | | | | |
| Caddy 2015 | | Diagnosis: Unipolar Depression  Resistant: Yes  Suicidal: No | Ketamine IV | Midazolam | NA - Single RCT | OR | Sample size: 72  Effect measure: 0.33 (0.05, 2.09)  p-value: 0.24 | Het.: I²: NA, p=NA  Pub.B. Test: NA, p=NA |
| Dean 2021 | | Diagnosis: Bipolar Depression  Resistant: Not reported in all of the RCTs  Suicidal: Yes | Ketamine IV | Midazolam | NA - Single RCT | OR | Sample size: 16  Effect measure: NA (NA, NA)  p-value: Not reported | Het.: I²: NA, p=NA  Pub.B. Test: NA, p=NA |
| Dean 2021 | | Diagnosis: Bipolar Depression  Resistant: Yes  Suicidal: Not reported in some of the RCTs | Ketamine IV | Saline | Random Effect | OR | Sample size: 33  Effect measure: 3.48 (0.56, 21.74)  p-value: 0.18 | Het.: I²: 0%, p=0.66  Pub.B. Test: NA, p=NA |
| Kishimoto 2016 | | Diagnosis: Unipolar Depression, Bipolar Depression  Resistant: Not reported in some of the RCTs  Suicidal: Not reported in some of the RCTs | Ketamine IV | Saline,Midazolam | Random Effect | RR | Sample size: 157  Effect measure: 1.14 (0.42, 3.1)  p-value: 0.81 | Het.: I²: 8.83%, p=0.36  Pub.B. Test: NA, p=NA |
| McGirr 2015 | | Diagnosis: Bipolar Depression, Unipolar Depression  Resistant: Not reported in some of the RCTs  Suicidal: Not reported in some of the RCTs | Ketamine IV,Ketamine IN | Saline,Midazolam | Random Effect | OR | Sample size: 175  Effect measure: 1.95 (0.86, 4.42)  p-value: 0.11 | Het.: I²: NA, p=NA  Pub.B. Test: NA, p=NA |
| Zheng 2020 | | Diagnosis: Unipolar Depression  Resistant: Not reported in some of the RCTs  Suicidal: Mixed | Esketamine IN | Saline | Random Effect | RR | Sample size: 267  Effect measure: 1.53 (0.9, 2.61)  p-value: 0.12 | Het.: I²: 32%, p=NA  Pub.B. Test: NA, p=NA |
| **Efficacy – Depressive symptoms**  **(<60 mins)** | | | | | | | | |
| Kishimoto 2016 | | Diagnosis: Unipolar Depression, Bipolar Depression  Resistant: Not reported in some of the RCTs  Suicidal: Not reported in some of the RCTs | Ketamine IV | Saline | Random Effect | Hedge's g | Sample size: 58  Effect measure: -0.5 (-1, 0)  p-value: 0.05 | Het.: I²: 44.3%, p=0.15  Pub.B. Test: NA, p=NA |
| **Efficacy – Depressive symptoms**  **(60-90 mins)** | | | | | | | | |
| Kishimoto 2016 | | Diagnosis: Bipolar Depression, Unipolar Depression  Resistant: Yes  Suicidal: Not reported in some of the RCTs | Ketamine IV | Saline | Random Effect | Hedge's g | Sample size: 51  Effect measure: -0.823 (-1.219, -0.426)  p-value: 0 | Het.: I²: NA, p=NA  Pub.B. Test: NA, p=NA |
| **Efficacy – Depressive symptoms**  **(90-120 mins)** | | | | | | | | |
| Kishimoto 2016 | | Diagnosis: Bipolar Depression, Unipolar Depression  Resistant: Yes  Suicidal: Not reported in some of the RCTs | Ketamine IV | Saline | Random Effect | Hedge's g | Sample size: 51  Effect measure: -0.863 (-1.26, -0.465)  p-value: 0 | Het.: I²: NA, p=NA  Pub.B. Test: NA, p=NA |
| **Efficacy – Depressive symptoms**  **(120-240 mins)** | | | | | | | | |
| An 2021 | | Diagnosis: Unipolar Depression  Resistant: Not reported in some of the RCTs  Suicidal: Mixed | Esketamine IN | Saline | Random Effect | WMD | Sample size: 66  Effect measure: -6.16 (-7.88, -4.44)  p-value: 0 | Het.: I²: 64.7%, p=0.037  Pub.B. Test: NA, p=NA |
| Kishimoto 2016 | | Diagnosis: Unipolar Depression, Bipolar Depression  Resistant: Not reported in some of the RCTs  Suicidal: Not reported in some of the RCTs | Ketamine IV | Saline | Random Effect | Hedge's g | Sample size: 62  Effect measure: -0.748 (-1.079, -0.418)  p-value: 0 | Het.: I²: NA, p=NA  Pub.B. Test: NA, p=NA |
| **Efficacy – Depressive symptoms**  **(24-48h)** | | | | | | | | |
| An 2021 | | Diagnosis: Unipolar Depression  Resistant: Yes  Suicidal: No | Esketamine IN | Saline | NA - Single RCT | WMD | Sample size: NA  Effect measure: -9.96 (-10.95, -8.97)  p-value: 0 | Het.: I²: 0%, p=0.435  Pub.B. Test: NA, p=NA |
| Caddy 2015 | | Diagnosis: Unipolar Depression  Resistant: Yes  Suicidal: Not reported in all of the RCTs | Ketamine IV | ECT | NA - Single RCT | MD | Sample size: 18  Effect measure: -8.9 (-11.72, -6.08)  p-value: 0.00001 | Het.: I²: NA, p=NA  Pub.B. Test: NA, p=NA |
| Caddy 2015 | | Diagnosis: Unipolar Depression  Resistant: Yes  Suicidal: No | Ketamine IV | Midazolam | NA - Single RCT | MD | Sample size: 72  Effect measure: -7.95 (-12.67, -3.23)  p-value: 0.001 | Het.: I²: NA, p=NA  Pub.B. Test: NA, p=NA |
| Caddy 2015 | | Diagnosis: Unipolar Depression  Resistant: Not reported in some of the RCTs  Suicidal: Not reported in some of the RCTs | Ketamine IV | Saline | Random Effect | SMD (Not Specified) | Sample size: 52  Effect measure: -1.42 (-2.26, -0.57)  p-value: 0.001 | Het.: I²: 36%, p=0.21  Pub.B. Test: NA, p=NA |
| Dean 2021 | | Diagnosis: Bipolar Depression  Resistant: Not reported in all of the RCTs  Suicidal: Yes | Ketamine IV | Midazolam | NA - Single RCT | MD | Sample size: 16  Effect measure: -5.85 (-12.13, 0.43)  p-value: 0.07 | Het.: I²: NA, p=NA  Pub.B. Test: NA, p=NA |
| Dean 2021 | | Diagnosis: Bipolar Depression  Resistant: Yes  Suicidal: Not reported in some of the RCTs | Ketamine IV | Saline | Random Effect | MD | Sample size: 33  Effect measure: -11.81 (-20.01, -3.61)  p-value: 0.005 | Het.: I²: 0%, p=0.47  Pub.B. Test: NA, p=NA |
| Hock 2022 | | Diagnosis: Unipolar Depression  Resistant: Yes  Suicidal: Mixed | Esketamine IN | Active Placebo | Random Effect | SMD | Sample size: NA  Effect measure: -0.35 (-0.49, -0.21)  p-value: 0.0001 | Het.: I²: NA, p=0.297  Pub.B. Test: NA, p=NA |
| Hock 2022 | | Diagnosis: Unipolar Depression  Resistant: Mixed  Suicidal: Yes | Esketamine IN | Active Placebo | Random Effect | SMD | Sample size: NA  Effect measure: -0.35 (-0.49, -0.21)  p-value: 0.0001 | Het.: I²: NA, p=0.297  Pub.B. Test: NA, p=NA |
| Hock 2022 | | Diagnosis: Unipolar Depression  Resistant: Mixed  Suicidal: Mixed | Esketamine IN | Active Placebo | Random Effect | SMD | Sample size: NA  Effect measure: -0.34 (-0.44, -0.24)  p-value: 0.0001 | Het.: I²: NA, p=0.446  Pub.B. Test: NA, p=NA |
| Kishimoto 2016 | | Diagnosis: Bipolar Depression, Unipolar Depression  Resistant: Yes  Suicidal: Not reported in some of the RCTs | Ketamine IV | Saline | Random Effect | Hedge's g | Sample size: 51  Effect measure: -0.879 (-1.202, -0.556)  p-value: 0 | Het.: I²: NA, p=NA  Pub.B. Test: NA, p=NA |
| Kishimoto 2016 | | Diagnosis: Unipolar Depression, Bipolar Depression  Resistant: Not reported in some of the RCTs  Suicidal: Not reported in some of the RCTs | Ketamine IV | Saline,Midazolam | Random Effect | Hedge's g | Sample size: 161  Effect measure: -1 (-1.28, -0.73)  p-value: 0.001 | Het.: I²: 0%, p=0.91  Pub.B. Test: NA, p=NA |
| Kryst 2020 | | Diagnosis: Bipolar Depression  Resistant: No Information  Suicidal: No Information | Ketamine | Placebo/Sham | Random Effect | SMD | Sample size:  Effect measure: -0.96 (-1.71, -0.21)  p-value: 0.01 | Het.: I²: %, p=0.46  Pub.B. Test: , p=NA |
| Kryst 2020 | | Diagnosis: Unipolar Depression  Resistant: No Information  Suicidal: No Information | Ketamine | Placebo/Sham | Random Effect | SMD | Sample size: NA  Effect measure: -0.89 (-1.29, -0.48)  p-value: 0.0001 | Het.: I²: 62%, p=0.004  Pub.B. Test: , p=NA |
| Lee 2015 | | Diagnosis: Bipolar Depression  Resistant: Yes  Suicidal: Not reported in some of the RCTs | Ketamine IV | Saline | Random Effect | SMD (Not Specified) | Sample size: 33  Effect measure: -1.29 (-1.89, -0.75)  p-value: 0.001 | Het.: I²: %, p=NA  Pub.B. Test: , p=NA |
| Lee 2015 | | Diagnosis: Bipolar Depression, Unipolar Depression  Resistant: Not reported in some of the RCTs  Suicidal: Not reported in some of the RCTs | Ketamine IV | Saline,Midazolam | Random Effect | SMD (Not Specified) | Sample size: 150  Effect measure: -1.01 (-1.34, -0.69)  p-value: 0.001 | Het.: I²: 30%, p=0.27  Pub.B. Test: NA, p=NA |
| Lee 2015 | | Diagnosis: Unipolar Depression  Resistant: Not reported in some of the RCTs  Suicidal: No | Ketamine IV | Saline,Midazolam | Random Effect | SMD (Not Specified) | Sample size: 117  Effect measure: -0.9 (-1.35, -0.46)  p-value: 0.001 | Het.: I²: %, p=NA  Pub.B. Test: , p=NA |
| Marcantoni 2020 | | Diagnosis: Unipolar Depression, Bipolar Depression  Resistant: Not reported in some of the RCTs  Suicidal: Not reported in some of the RCTs | Ketamine IV | Saline,Midazolam | Random Effect | Hedge's g | Sample size: 314  Effect measure: -0.77 (-1.08, -0.46)  p-value: Not reported | Het.: I²: 24%, p=0.25  Pub.B. Test: Egger, Funnel Plots, Rosenthal's fail-safe N, p=0.0013 |
| McGirr 2015 | | Diagnosis: Bipolar Depression  Resistant: Yes  Suicidal: Not reported in some of the RCTs | Ketamine IV | Saline | Random Effect | Rosenthal's r | Sample size: 33  Effect measure: -0.68 (-0.86, -0.5)  p-value: 0.001 | Het.: I²: NA, p=NA  Pub.B. Test: NA, p=NA |
| McGirr 2015 | | Diagnosis: Unipolar Depression  Resistant: Not reported in some of the RCTs  Suicidal: Not reported in some of the RCTs | Ketamine IV | Saline,Midazolam | Random Effect | Rosenthal's r | Sample size: 124  Effect measure: -1.21 (-1.5, -0.93)  p-value: 0.001 | Het.: I²: NA, p=NA  Pub.B. Test: NA, p=NA |
| McGirr 2015 | | Diagnosis: Unipolar Depression  Resistant: Not reported in some of the RCTs  Suicidal: Not reported in some of the RCTs | Ketamine IV,Ketamine IN | Saline,Midazolam | Random Effect | Rosenthal's r | Sample size: 142  Effect measure: -1.07 (-1.42, -0.72)  p-value: 0 | Het.: I²: NA, p=NA  Pub.B. Test: NA, p=NA |
| McGirr 2015 | | Diagnosis: Unipolar Depression, Bipolar Depression  Resistant: Not reported in some of the RCTs  Suicidal: Not reported in some of the RCTs | Ketamine IV,Ketamine IN | Saline,Midazolam | Random Effect | Rosenthal's r | Sample size: 175  Effect measure: -0.9 (-1.13, -0.66)  p-value: 0.001 | Het.: I²: NA, p=NA  Pub.B. Test: NA, p=NA |
| Romeo 2015 | | Diagnosis: Unipolar Depression  Resistant: Not reported in some of the RCTs  Suicidal: Not reported in some of the RCTs | Ketamine IV,Ketamine IN | Saline | Random Effect | Cohen's d | Sample size: 43  Effect measure: -0.81 (-1.44, -0.19)  p-value: 0.01 | Het.: I²: 0.45%, p=0.16  Pub.B. Test: Funnel Plots, p=NA |
| Romeo 2015 | | Diagnosis: Unipolar Depression, Bipolar Depression  Resistant: Not reported in some of the RCTs  Suicidal: Not reported in some of the RCTs | Ketamine IV | Saline | Random Effect | Cohen's d | Sample size: 85  Effect measure: -1.04 (-1.37, -0.71)  p-value: 0.00001 | Het.: I²: 3%, p=0.39  Pub.B. Test: NA, p=NA |
| Romeo 2015 | | Diagnosis: Unipolar Depression, Bipolar Depression  Resistant: Not reported in some of the RCTs  Suicidal: Not reported in some of the RCTs | Ketamine IV | Saline | Random Effect | Cohen's d | Sample size: 85  Effect measure: -1.24 (-1.64, -0.83)  p-value: 0.00001 | Het.: I²: 0%, p=0.55  Pub.B. Test: NA, p=NA |
| Romeo 2015 | | Diagnosis: Unipolar Depression, Bipolar Depression  Resistant: Not reported in some of the RCTs  Suicidal: Not reported in some of the RCTs | Ketamine IV,Ketamine IN | Saline | Random Effect | Cohen's d | Sample size: 103  Effect measure: -0.89 (-1.25, -0.54)  p-value: 0.00001 | Het.: I²: 1%, p=0.39  Pub.B. Test: Funnel Plots, p=NA |
| Romeo 2015 | | Diagnosis: Unipolar Depression, Bipolar Depression  Resistant: Not reported in some of the RCTs  Suicidal: Not reported in some of the RCTs | Ketamine IV,Ketamine IN | Saline | Random Effect | Cohen's d | Sample size: 103  Effect measure: -1 (-1.3, -0.71)  p-value: 0.00001 | Het.: I²: 0%, p=NA  Pub.B. Test: Funnel Plots, p=NA |
| Romeo 2015 | | Diagnosis: Unipolar Depression, Bipolar Depression  Resistant: Not reported in some of the RCTs  Suicidal: Not reported in some of the RCTs | Ketamine IV,Ketamine IN | Saline | Random Effect | Cohen's d | Sample size: 88  Effect measure: -1.03 (-1.45, -0.6)  p-value: 0.00001 | Het.: I²: 33%, p=NA  Pub.B. Test: Funnel Plots, p=NA |
| Romeo 2015 | | Diagnosis: Unipolar Depression, Bipolar Depression  Resistant: Not reported in some of the RCTs  Suicidal: Not reported in some of the RCTs | Ketamine IV,Ketamine IN | Saline | Random Effect | Cohen's d | Sample size: 76  Effect measure: -1.34 (-1.88, -0.8)  p-value: 0.00001 | Het.: I²: 0%, p=0.68  Pub.B. Test: Funnel Plots, p=NA |
| **Efficacy – Depressive symptoms**  **(3-6 days)** | | | | | | | | |
| Caddy 2015 | | Diagnosis: Unipolar Depression  Resistant: Yes  Suicidal: Not reported in all of the RCTs | Ketamine IV | ECT | NA - Single RCT | MD | Sample size: 18  Effect measure: -3.4 (-5.99, -0.81)  p-value: 0.0008 | Het.: I²: NA, p=NA  Pub.B. Test: NA, p=NA |
| Caddy 2015 | | Diagnosis: Unipolar Depression  Resistant: Yes  Suicidal: No | Ketamine IV | Midazolam | NA - Single RCT | MD | Sample size: 72  Effect measure: -6.8 (-13.82, 0.22)  p-value: 0.06 | Het.: I²: NA, p=NA  Pub.B. Test: NA, p=NA |
| Caddy 2015 | | Diagnosis: Unipolar Depression  Resistant: Not reported in some of the RCTs  Suicidal: Not reported in some of the RCTs | Ketamine IV | Saline | Random Effect | SMD (Not Specified) | Sample size: 52  Effect measure: -1.21 (-1.82, -0.59)  p-value: 0.0001 | Het.: I²: 0%, p=1  Pub.B. Test: NA, p=NA |
| Dean 2021 | | Diagnosis: Bipolar Depression  Resistant: Yes  Suicidal: Not reported in some of the RCTs | Ketamine IV | Saline | Random Effect | MD | Sample size: 33  Effect measure: -9.1 (-16, -2.21)  p-value: 0.01 | Het.: I²: 0%, p=0.6  Pub.B. Test: NA, p=NA |
| Kishimoto 2016 | | Diagnosis: Bipolar Depression, Unipolar Depression  Resistant: Not reported in some of the RCTs  Suicidal: Not reported in some of the RCTs | Ketamine IV | Saline | Random Effect | Hedge's g | Sample size: 89  Effect measure: -0.723 (-1, -0.445)  p-value: 0 | Het.: I²: NA, p=NA  Pub.B. Test: NA, p=NA |
| Kishimoto 2016 | | Diagnosis: Bipolar Depression, Unipolar Depression  Resistant: Not reported in some of the RCTs  Suicidal: Not reported in some of the RCTs | Ketamine IV | Saline | Random Effect | Hedge's g | Sample size: 82  Effect measure: -0.38 (-0.73, -0.03)  p-value: 0.036 | Het.: I²: 9.38%, p=0.35  Pub.B. Test: NA, p=NA |
| Kryst 2020 | | Diagnosis: Bipolar Depression  Resistant: No Information  Suicidal: No Information | Ketamine | Placebo/Sham | Random Effect | SMD | Sample size:  Effect measure: -0.87 (-1.62, -0.11)  p-value: 0.02 | Het.: I²: %, p=0.47  Pub.B. Test: , p=NA |
| Kryst 2020 | | Diagnosis: Unipolar Depression  Resistant: No Information  Suicidal: No Information | Ketamine | Placebo/Sham | Random Effect | SMD | Sample size:  Effect measure: -0.75 (-0.99, -0.51)  p-value: 0.00001 | Het.: I²: %, p=0.5  Pub.B. Test: , p=NA |
| McIntyre 2020 | | Diagnosis: No Information  Resistant: No Information  Suicidal: No information | Esketamine IN | Placebo/Sham | NA - Single RCT | Hedge's g | Sample size: NA  Effect measure: -0.487 (-0.83, -0.143)  p-value: 0.01 | Het.: I²: 62.54%, p=0.014  Pub.B. Test: Egger, p=NA |
| McIntyre 2020 | | Diagnosis: No Information  Resistant: No Information  Suicidal: No information | Esketamine IV | Placebo/Sham | Random Effect | Hedge's g | Sample size: NA  Effect measure: -1.637 (-2.283, -0.99)  p-value: 0.01 | Het.: I²: NA, p=NA  Pub.B. Test: Egger, p=NA |
| McIntyre 2020 | | Diagnosis: Unipolar Depression  Resistant: Not reported in some of the RCTs  Suicidal: Mixed | Ketamine IN | Saline | Random Effect | Hedge's g | Sample size: 84  Effect measure: -0.529 (-0.835, -0.223)  p-value: 0.01 | Het.: I²: 45.07%, p=0.016  Pub.B. Test: Egger, p=NA |
| Romeo 2015 | | Diagnosis: Unipolar Depression, Bipolar Depression  Resistant: Not reported in some of the RCTs  Suicidal: Not reported in some of the RCTs | Ketamine IV | Saline | Random Effect | Cohen's d | Sample size: 85  Effect measure: -0.87 (-1.19, -0.55)  p-value: 0.00001 | Het.: I²: 0%, p=0.47  Pub.B. Test: Funnel Plots, p=NA |
| Romeo 2015 | | Diagnosis: Unipolar Depression, Bipolar Depression  Resistant: Not reported in some of the RCTs  Suicidal: Not reported in some of the RCTs | Ketamine IV,Ketamine IN | Saline | Random Effect | Cohen's d | Sample size: 103  Effect measure: -0.77 (-1.1, -0.44)  p-value: 0.00001 | Het.: I²: 22%, p=NA  Pub.B. Test: Funnel Plots, p=NA |
| **Efficacy – Depressive symptoms**  **(7-13 days)** | | | | | | | | |
| Caddy 2015 | | Diagnosis: Unipolar Depression  Resistant: Yes  Suicidal: Not reported in all of the RCTs | Ketamine IV | ECT | NA - Single RCT | MD | Sample size: 18  Effect measure: -1 (-3.45, 1.45)  p-value: 0.004 | Het.: I²: NA, p=NA  Pub.B. Test: NA, p=NA |
| Caddy 2015 | | Diagnosis: Unipolar Depression  Resistant: Yes  Suicidal: No | Ketamine IV | Midazolam | NA - Single RCT | MD | Sample size: 72  Effect measure: -5.69 (-13.32, 1.94)  p-value: 0.14 | Het.: I²: NA, p=NA  Pub.B. Test: NA, p=NA |
| Dean 2021 | | Diagnosis: Bipolar Depression  Resistant: Yes  Suicidal: Not reported in some of the RCTs | Ketamine IV | Saline | Random Effect | MD | Sample size: 33  Effect measure: -0.88 (-5.88, 4.12)  p-value: 0.73 | Het.: I²: 0%, p=0.88  Pub.B. Test: NA, p=NA |
| Kishimoto 2016 | | Diagnosis: Bipolar Depression  Resistant: Yes  Suicidal: Not reported in some of the RCTs | Ketamine IV | Saline | Random Effect | Hedge's g | Sample size: 33  Effect measure: -0.057 (-0.529, 0.414)  p-value: 0.812 | Het.: I²: NA, p=NA  Pub.B. Test: NA, p=NA |
| Kryst 2020 | | Diagnosis: Bipolar Depression  Resistant: No Information  Suicidal: No Information | Ketamine | Placebo/Sham | Random Effect | SMD | Sample size:  Effect measure: -0.14 (-0.89, 0.61)  p-value: 0.72 | Het.: I²: %, p=0.98  Pub.B. Test: , p=NA |
| Kryst 2020 | | Diagnosis: Unipolar Depression  Resistant: No Information  Suicidal: No Information | Ketamine | Placebo/Sham | Random Effect | SMD | Sample size:  Effect measure: -0.43 (-0.84, -0.02)  p-value: 0.04 | Het.: I²: %, p=0.004  Pub.B. Test: , p=NA |
| Lee 2015 | | Diagnosis: Bipolar Depression  Resistant: Yes  Suicidal: Not reported in some of the RCTs | Ketamine IV | Saline | Random Effect | SMD (Not Specified) | Sample size: 33  Effect measure: -0.28 (-0.76, 0.21)  p-value: 0.26 | Het.: I²: %, p=NA  Pub.B. Test: , p=NA |
| Lee 2015 | | Diagnosis: Bipolar Depression, Unipolar Depression  Resistant: Not reported in some of the RCTs  Suicidal: Not reported in some of the RCTs | Ketamine IV | Saline,Midazolam | Random Effect | SMD (Not Specified) | Sample size: 150  Effect measure: -0.41 (-0.68, -0.14)  p-value: 0.003 | Het.: I²: 0%, p=0.87  Pub.B. Test: NA, p=NA |
| Lee 2015 | | Diagnosis: Unipolar Depression  Resistant: Not reported in some of the RCTs  Suicidal: No | Ketamine IV | Saline,Midazolam | Random Effect | SMD (Not Specified) | Sample size: 117  Effect measure: -0.46 (-0.78, -0.14)  p-value: 0 | Het.: I²: %, p=NA  Pub.B. Test: , p=NA |
| Marcantoni 2020 | | Diagnosis: Unipolar Depression  Resistant: Not reported in some of the RCTs  Suicidal: No | Ketamine IV | Saline,Midazolam | Random Effect | Hedge's g | Sample size: 117  Effect measure: -0.49 (-0.78, -0.2)  p-value: 0.051 | Het.: I²: 0%, p=0.36  Pub.B. Test: Egger, Funnel Plots, Rosenthal's fail-safe N, p=0.0013 |
| McIntyre 2020 | | Diagnosis: Unipolar Depression  Resistant: Not reported in some of the RCTs  Suicidal: Mixed | Esketamine IN | Placebo/Active Placebo | Random Effect | Hedge's g | Sample size: 66  Effect measure: -1.018 (-1.538, -0.499)  p-value: 0.01 | Het.: I²: 62.54%, p=0.014  Pub.B. Test: Egger, p=NA |
| McIntyre 2020 | | Diagnosis: Unipolar Depression  Resistant: Not reported in some of the RCTs  Suicidal: Not reported in all of the RCTs | Oral Ketamine | Placebo/Active Placebo | Random Effect | Hedge's g | Sample size: 121  Effect measure: -0.657 (-1.048, -0.267)  p-value: 0.01 | Het.: I²: NA, p=NA  Pub.B. Test: Egger, p=NA |
| McIntyre 2020 | | Diagnosis: Unipolar Depression  Resistant: Not reported in some of the RCTs  Suicidal: Not reported in some of the RCTs | Oral Ketamine | Saline,Diclofenac | Random Effect | Hedge's g | Sample size: 80  Effect measure: -0.392 (-0.694, -0.089)  p-value: 0.01 | Het.: I²: NA, p=NA  Pub.B. Test: Egger, p=NA |
| Romeo 2015 | | Diagnosis: Bipolar Depression, Unipolar Depression  Resistant: Not reported in some of the RCTs  Suicidal: Not reported in some of the RCTs | Ketamine IV,Ketamine IN | Saline | Random Effect | Cohen's d | Sample size: 96  Effect measure: -0.36 (-0.65, -0.08)  p-value: 0.01 | Het.: I²: 0%, p=NA  Pub.B. Test: Funnel Plots, p=NA |
| **Efficacy – Depressive symptoms**  **(14-28 days)** | | | | | | | | |
| An 2021 | | Diagnosis: Unipolar Depression  Resistant: Yes  Suicidal: No | Esketamine IN | Saline | Random Effect | WMD | Sample size: 338  Effect measure: -4.09 (-6, -2.18)  p-value: 0 | Het.: I²: 0%, p=0.995  Pub.B. Test: NA, p=NA |
| Caddy 2015_(was_Amit_2015) | | Diagnosis: Unipolar Depression  Resistant: Yes  Suicidal: Not reported in all of the RCTs | Ketamine IV | ECT | NA - Single RCT | MD | Sample size: 18  Effect measure: 1.2 (-1.2, 3.6)  p-value: 0.06 | Het.: I²: NA, p=NA  Pub.B. Test: NA, p=NA |
| Dean 2021 | | Diagnosis: Bipolar Depression  Resistant: Yes  Suicidal: Not reported in some of the RCTs | Ketamine IV | Saline | Random Effect | MD | Sample size: 33  Effect measure: -1.14 (-6.3, 4.01)  p-value: 0.66 | Het.: I²: 0%, p=0.63  Pub.B. Test: NA, p=NA |
| Fornaro 2020 | | Diagnosis: Bipolar Depression  Resistant: Yes  Suicidal: Not reported in some of the RCTs | Ketamine IV | Saline | Random Effect | SMD (Not Specified) | Sample size: 33  Effect measure: 0.352 (-0.135, 0.84)  p-value: 0.156 | Het.: I²: 0%, p=0.156  Pub.B. Test: Egger, Begg-Mazumdar Kendall's tau, p=NA |
| Jawad 2022 | | Diagnosis: Unipolar Depression  Resistant: Not reported in some of the RCTs  Suicidal: Mixed | Esketamine IN | Saline | Random Effect | Cohen's d | Sample size: 855  Effect measure: -0.239 (-0.335, -0.142)  p-value: 0.0001 | Het.: I²: 57%, p=NA  Pub.B. Test: Egger, p=0.23 |
| Kishimoto 2016 | | Diagnosis: Bipolar Depression  Resistant: Yes  Suicidal: Not reported in some of the RCTs | Ketamine IV | Saline | Random Effect | Hedge's g | Sample size: 33  Effect measure: -0.313 (-1.115, 0.489)  p-value: 0.444 | Het.: I²: NA, p=NA  Pub.B. Test: NA, p=NA |
| McIntyre 2020 | | Diagnosis: Unipolar Depression  Resistant: Not reported in some of the RCTs  Suicidal: Mixed | Esketamine IN | Placebo/Sham | Random Effect | Hedge's g | Sample size: 66  Effect measure: -0.417 (-0.596, -0.238)  p-value: 0.01 | Het.: I²: 62.54%, p=0.014  Pub.B. Test: Egger, p=NA |
| McIntyre 2020 | | Diagnosis: Unipolar Depression  Resistant: Not reported in some of the RCTs  Suicidal: Mixed | Ketamine IN | Placebo/Sham | Random Effect | Hedge's g | Sample size: 66  Effect measure: -0.417 (-0.596, -0.238)  p-value: 0.01 | Het.: I²: 45.07%, p=0.016  Pub.B. Test: Egger, p=NA |
| McIntyre 2020 | | Diagnosis: Unipolar Depression  Resistant: Not reported in some of the RCTs  Suicidal: Mixed | Ketamine IV | Placebo/Sham | Random Effect | Hedge's g | Sample size: 66  Effect measure: -0.293 (-0.509, -0.077)  p-value: 0.01 | Het.: I²: 59.54%, p=0.015  Pub.B. Test: Egger, p=NA |
| McIntyre 2020 | | Diagnosis: Unipolar Depression  Resistant: Not reported in some of the RCTs  Suicidal: Not reported in all of the RCTs | Oral Ketamine | Placebo/Sham | Random Effect | Hedge's g | Sample size: 121  Effect measure: -0.633 (-0.898, -0.368)  p-value: 0.01 | Het.: I²: NA, p=NA  Pub.B. Test: Egger, p=NA |
| Nuñez 2020 | | Diagnosis: Unipolar Depression  Resistant: Not reported in some of the RCTs  Suicidal: Not reported in some of the RCTs | Ketamine Oral | Diclofenac,Saline | Random Effect | SMD (Not Specified) | Sample size: 80  Effect measure: -0.79 (-1.4, -0.17)  p-value: 0.012 | Het.: I²: 43.76%, p=0.1824  Pub.B. Test: NA, p=NA |
| Nuñez 2020 | | Diagnosis: Unipolar Depression  Resistant: Not reported in some of the RCTs  Suicidal: Not reported in all of the RCTs | Ketamine Oral | Saline | Random Effect | SMD (Not Specified) | Sample size: 121  Effect measure: -0.71 (-1.08, -0.35)  p-value: 0.001 | Het.: I²: 0%, p=0.7523  Pub.B. Test: NA, p=NA |
| Papadimitropoulou 2017 | | Diagnosis: Unipolar Depression  Resistant: Yes  Suicidal: No | Ketamine IV | Saline | Random Effect | MD | Sample size: NA  Effect measure: -14 (-19.9, -8)  p-value: Not reported | Het.: I²: NA, p=NA  Pub.B. Test: NA, p=NA |
| Romeo 2015 | | Diagnosis: Bipolar Depression  Resistant: Yes  Suicidal: Not reported in some of the RCTs | Ketamine IV | Saline | Random Effect | Cohen's d | Sample size: 33  Effect measure: -0.38 (-0.87, 0.11)  p-value: 0.13 | Het.: I²: 0%, p=0.93  Pub.B. Test: Funnel Plots, p=NA |
| **Efficacy – Depressive symptoms**  **(Endpoint)** | | | | | | | | |
| An 2021 | | Diagnosis: Unipolar Depression  Resistant: Not reported in some of the RCTs  Suicidal: Mixed | Esketamine IN | Saline | Random Effect | WMD | Sample size: 404  Effect measure: -6.74 (-8.32, -5.17)  p-value: 0 | Het.: I²: 80.7%, p=0  Pub.B. Test: NA, p=NA |
| Bahji 2020 | | Diagnosis: Bipolar Depression  Resistant: Yes  Suicidal: Not reported in some of the RCTs | Ketamine IV | Saline | Random Effect | SMD (Not Specified) | Sample size: 33  Effect measure: -2.23 (-3.88, -0.59)  p-value: Not reported | Het.: I²: 96.5%, p=0.0001  Pub.B. Test: NA, p=NA |
| Fond 2014 | | Diagnosis: Unipolar Depression, Bipolar Depression  Resistant: Not reported in some of the RCTs  Suicidal: Not reported in some of the RCTs | Ketamine IV | Saline,ECT,Midazolam | Random Effect | SMD (Not Specified) | Sample size: 175  Effect measure: -1.1 (-1.39, -0.81)  p-value: 0.01 | Het.: I²: 2.9%, p=0.001  Pub.B. Test: Egger, Rosenthal's fail-safe N, p=0.76 |
| Hock 2022 | | Diagnosis: Unipolar Depression  Resistant: Yes  Suicidal: Mixed | Esketamine IN | Active Placebo | Random Effect | SMD | Sample size: NA  Effect measure: -0.27 (-0.42, -0.13)  p-value: 0.0001 | Het.: I²: NA, p=0.147  Pub.B. Test: NA, p=NA |
| Hock 2022 | | Diagnosis: Unipolar Depression  Resistant: Mixed  Suicidal: Yes | Esketamine IN | Active Placebo | Random Effect | SMD | Sample size: NA  Effect measure: -0.27 (-0.42, -0.13)  p-value: 0.0002 | Het.: I²: NA, p=0.147  Pub.B. Test: NA, p=NA |
| Hock 2022 | | Diagnosis: Unipolar Depression  Resistant: Mixed  Suicidal: Mixed | Esketamine IN | Active Placebo | Random Effect | SMD | Sample size: NA  Effect measure: -0.26 (-0.37, -0.16)  p-value: 0.004 | Het.: I²: NA, p=0.288  Pub.B. Test: NA, p=NA |
| Marcantoni 2020 | | Diagnosis: Unipolar Depression, Bipolar Depression  Resistant: Not reported in some of the RCTs  Suicidal: Not reported in some of the RCTs | Ketamine IV | Saline,Midazolam | Random Effect | Hedge's g | Sample size: 250  Effect measure: -0.69 (-0.94, -0.44)  p-value: 0.0001 | Het.: I²: 9.26%, p=0.36  Pub.B. Test: Egger, Funnel Plots, Rosenthal's fail-safe N, p=0.013 |
| McIntyre 2020 | | Diagnosis: Unipolar Depression  Resistant: Not reported in some of the RCTs  Suicidal: Mixed | Esketamine IN,Ketamine IN | Saline | Random Effect | Hedge's g | Sample size: 84  Effect measure: -0.667 (-0.882, -0.451)  p-value: 0.01 | Het.: I²: 45.07%, p=0.016  Pub.B. Test: Egger, p=NA |
| McIntyre 2020 | | Diagnosis: Unipolar Depression  Resistant: Not reported in some of the RCTs  Suicidal: Not reported in some of the RCTs | Ketamine Oral | Diclofenac,Saline | Random Effect | Hedge's g | Sample size: 161  Effect measure: -0.556 (-0.887, -0.224)  p-value: Not reported | Het.: I²: NA, p=NA  Pub.B. Test: Egger, p=NA |
| McIntyre 2020 | | Diagnosis: Unipolar Depression, Bipolar Depression  Resistant: Not reported in some of the RCTs  Suicidal: Not reported in some of the RCTs | Oral Ketamine | Saline,Diclofenac | Random Effect | Hedge's g | Sample size: 161  Effect measure: -0.556 (-0.887, -0.224)  p-value: Not reported | Het.: I²: NA, p=NA  Pub.B. Test: Egger, p=NA |
| Nuñez 2020 | | Diagnosis: Unipolar Depression  Resistant: Not reported in some of the RCTs  Suicidal: Not reported in some of the RCTs | Ketamine Oral | Diclofenac,Saline | Random Effect | SMD (Not Specified) | Sample size: 161  Effect measure: -0.75 (-1.08, -0.43)  p-value: 0.0001 | Het.: I²: 0%, p=0.4476  Pub.B. Test: NA, p=NA |
| Papakostas 2020 | | Diagnosis: Unipolar Depression  Resistant: Not reported in some of the RCTs  Suicidal: Mixed | Esketamine IN | Saline | Random Effect | SMD (Not Specified) | Sample size: 404  Effect measure: -0.36 (-0.49, -0.24)  p-value: 0.0001 | Het.: I²: NA, p=NA  Pub.B. Test: NA, p=NA |
| Rhee 2022 | | Diagnosis: Unipolar Depression, Bipolar Depression  Resistant: Yes  Suicidal: Not reported in all of the RCTs | Ketamine IV | ECT | Random Effect | Hedge's g | Sample size: 43  Effect measure: -0.859 (-1.5, -0.218)  p-value: Not reported | Het.: I²: 60%, p=0.11  Pub.B. Test: Egger, Begg-Mazumdar Kendall's tau, p=Egger test (P = .32) and Begg and Mazumdar test (P = .57). |
| Rhee 2022 | | Diagnosis: Unipolar Depression, Bipolar Depression  Resistant: Yes  Suicidal: Not reported in all of the RCTs | Ketamine IV,Ketamine Oral,Ketamine IM | ECT | Random Effect | Hedge's g | Sample size: 65  Effect measure: -0.832 (-1.221, -0.444)  p-value: Not reported | Het.: I²: 22%, p=0.28  Pub.B. Test: Egger, Begg-Mazumdar Kendall's tau, p=Egger test (P = .32) and Begg and Mazumdar test (P = .57). |
| Zheng 2020 | | Diagnosis: Unipolar Depression  Resistant: No Information  Suicidal: No information | Esketamine IN | Saline | Random Effect | Hedge's g | Sample size: 518  Effect measure: -0.3 (-0.48, -0.13)  p-value: 0.0008 | Het.: I²: 0%, p=0.96  Pub.B. Test: NA, p=NA |
| **Efficacy – Remission**  **(<60 mins)** | | | | | | | | |
| Kishimoto 2016 | | Diagnosis: Bipolar Depression  Resistant: Yes  Suicidal: Not reported in some of the RCTs | Ketamine IV | Saline | Random Effect | RR | Sample size: 33  Effect measure: 3.924 (0.456, 33.765)  p-value: 0.213 | Het.: I²: NA, p=NA  Pub.B. Test: NA, p=NA |
| Newport 2015 | | Diagnosis: Bipolar Depression, Unipolar Depression  Resistant: Yes  Suicidal: Not reported in some of the RCTs | Ketamine IV,Ketamine IN | Saline | NR | OR | Sample size: 69  Effect measure: 2.6 (0.5, 13.8)  p-value: 0.26 | Het.: I²: NA, p=NA  Pub.B. Test: NA, p=NA |
| **Efficacy – Remission**  **(60-90 mins)** | | | | | | | | |
| Kishimoto 2016 | | Diagnosis: Bipolar Depression, Unipolar Depression  Resistant: Yes  Suicidal: Not reported in some of the RCTs | Ketamine IV | Saline | Random Effect | RR | Sample size: 51  Effect measure: 6.631 (1.23, 35.7)  p-value: 0.028 | Het.: I²: 0%, p=0.91  Pub.B. Test: NA, p=NA |
| Newport 2015 | | Diagnosis: Bipolar Depression, Unipolar Depression  Resistant: Yes  Suicidal: Not reported in some of the RCTs | Ketamine IV | Saline | NR | OR | Sample size: 51  Effect measure: 7.3 (1.4, 39.3)  p-value: 0.02 | Het.: I²: NA, p=NA  Pub.B. Test: NA, p=NA |
| **Efficacy – Remission**  **(90-120 mins)** | | | | | | | | |
| Kishimoto 2016 | | Diagnosis: Bipolar Depression, Unipolar Depression  Resistant: Yes  Suicidal: Not reported in some of the RCTs | Ketamine IV | Saline | Random Effect | RR | Sample size: 51  Effect measure: 8.532 (1.629, 44.684)  p-value: 0.011 | Het.: I²: NA, p=NA  Pub.B. Test: NA, p=NA |
| Newport 2015 | | Diagnosis: Bipolar Depression, Unipolar Depression  Resistant: Yes  Suicidal: Not reported in some of the RCTs | Ketamine IV | Saline | NR | OR | Sample size: 51  Effect measure: 10.3 (1.9, 55.8)  p-value: 0.007 | Het.: I²: NA, p=NA  Pub.B. Test: NA, p=NA |
| Zheng 2020 | | Diagnosis: Unipolar Depression  Resistant: No Information  Suicidal: No information | Esketamine IN | Saline | Random Effect | RR | Sample size: 133  Effect measure: 7.71 (2.16, 27.55)  p-value: 0.002 | Het.: I²: 0%, p=0.97  Pub.B. Test: NA, p=NA |
| **Efficacy – Remission**  **(120-240 mins)** | | | | | | | | |
| Kishimoto 2016 | | Diagnosis: Bipolar Depression, Unipolar Depression  Resistant: Yes  Suicidal: Not reported in some of the RCTs | Ketamine IV | Saline | Random Effect | RR | Sample size: 51  Effect measure: 9.427 (1.824, 48.72)  p-value: 0.007 | Het.: I²: NA, p=NA  Pub.B. Test: NA, p=NA |
| Newport 2015 | | Diagnosis: Bipolar Depression, Unipolar Depression  Resistant: Yes  Suicidal: Not reported in some of the RCTs | Ketamine IV,Ketamine IN | Saline | NR | OR | Sample size: 69  Effect measure: 11.8 (2.2, 64.1)  p-value: 0.004 | Het.: I²: NA, p=NA  Pub.B. Test: NA, p=NA |
| **Efficacy – Remission**  **(24-48h)** | | | | | | | | |
| An 2021 | | Diagnosis: Unipolar Depression  Resistant: Not reported in some of the RCTs  Suicidal: Mixed | Esketamine IN | Saline | Random Effect | RR | Sample size: 66  Effect measure: 3.55 (1.5, 8.38)  p-value: 0.001 | Het.: I²: 60.2%, p=0.113  Pub.B. Test: NA, p=NA |
| Caddy 2015 | | Diagnosis: Unipolar Depression  Resistant: Yes  Suicidal: Not reported in all of the RCTs | Ketamine IV | ECT | NA - Single RCT | OR | Sample size: 18  Effect measure: 3.35 (0.12, 93.83)  p-value: 0.48 | Het.: I²: NA, p=NA  Pub.B. Test: NA, p=NA |
| Caddy 2015 | | Diagnosis: Unipolar Depression  Resistant: Yes  Suicidal: Not reported in all of the RCTs | Ketamine IV | ECT | NA - Single RCT | OR | Sample size: 18  Effect measure: 3.35 (0.12, 93.83)  p-value: 0.48 | Het.: I²: NA, p=NA  Pub.B. Test: NA, p=NA |
| Caddy 2015 | | Diagnosis: Unipolar Depression  Resistant: Yes  Suicidal: No | Ketamine IV | Midazolam | NA - Single RCT | OR | Sample size: 72  Effect measure: 4.16 (1.08, 15.95)  p-value: 0.04 | Het.: I²: NA, p=NA  Pub.B. Test: NA, p=NA |
| Caddy 2015 | | Diagnosis: Unipolar Depression  Resistant: Not reported in some of the RCTs  Suicidal: No | Ketamine IV | Saline | Random Effect | OR | Sample size: 45  Effect measure: 6.6 (0.96, 45.09)  p-value: 0.054 | Het.: I²: 0%, p=0.97  Pub.B. Test: NA, p=NA |
| Dean 2021 | | Diagnosis: Bipolar Depression  Resistant: Not reported in all of the RCTs  Suicidal: Yes | Ketamine IV | Midazolam | NA - Single RCT | OR | Sample size: 16  Effect measure: 1.33 (0.07, 25.91)  p-value: 0.85 | Het.: I²: NA, p=NA  Pub.B. Test: NA, p=NA |
| Dean 2021 | | Diagnosis: Bipolar Depression  Resistant: Yes  Suicidal: Not reported in some of the RCTs | Ketamine IV | Saline | Random Effect | OR | Sample size: 33  Effect measure: 5.16 (0.51, 52.3)  p-value: 0.16 | Het.: I²: 0%, p=0.72  Pub.B. Test: NA, p=NA |
| Han 2016 | | Diagnosis: Unipolar Depression  Resistant: Not reported in some of the RCTs  Suicidal: Not reported in some of the RCTs | Ketamine IV | Saline,ECT,Midazolam | Random Effect | OR | Sample size: 169  Effect measure: 5.25 (1.82, 15.17)  p-value: 0.002 | Het.: I²: 0%, p=0.94  Pub.B. Test: NA, p=NA |
| Hock 2022 | | Diagnosis: Unipolar Depression  Resistant: No Information  Suicidal: | Esketamine IN | Saline | Random Effect | RR | Sample size:  Effect measure: 2.31 (NA, NA)  p-value: 0.0001 | Het.: I²: %, p=0.7  Pub.B. Test: NA, p=NA |
| Kishimoto 2016 | | Diagnosis: Bipolar Depression, Unipolar Depression  Resistant: Yes  Suicidal: Not reported in some of the RCTs | Ketamine IV | Saline | Random Effect | RR | Sample size: 55  Effect measure: 9.885 (2.412, 40.501)  p-value: 0 | Het.: I²: 0%, p=0.96  Pub.B. Test: NA, p=NA |
| Kishimoto 2016 | | Diagnosis: Bipolar Depression, Unipolar Depression  Resistant: Yes  Suicidal: Not reported in some of the RCTs | Ketamine IV | Saline | Random Effect | RR | Sample size: 51  Effect measure: 7.467 (1.386, 40.244)  p-value: 0.019 | Het.: I²: NA, p=NA  Pub.B. Test: NA, p=NA |
| Kryst 2020 | | Diagnosis: Bipolar Depression, Unipolar Depression  Resistant: Not reported in some of the RCTs  Suicidal: Not reported in some of the RCTs | Ketamine IV | Saline | Random Effect | OR | Sample size: 105  Effect measure: 5.45 (1.41, 21)  p-value: 0.01 | Het.: I²: 0%, p=0.99  Pub.B. Test: NA, p=NA |
| Marcantoni 2020 | | Diagnosis: Unipolar Depression, Bipolar Depression  Resistant: Not reported in some of the RCTs  Suicidal: No | Ketamine IV | Saline,Midazolam | Random Effect | OR | Sample size: 135  Effect measure: 6.53 (1.13, 37.62)  p-value: Not reported | Het.: I²: 0%, p=0.69  Pub.B. Test: Egger, Funnel Plots, Rosenthal's fail-safe N, p=0.0013 |
| McGirr 2015 | | Diagnosis: Bipolar Depression, Unipolar Depression  Resistant: Not reported in some of the RCTs  Suicidal: Not reported in some of the RCTs | Ketamine IV | Saline,Midazolam | Random Effect | OR | Sample size: 150  Effect measure: 7.07 (2.5, 19.95)  p-value: 0.001 | Het.: I²: NA, p=0.61  Pub.B. Test: Egger, Fail-safe N, p=0.230 |
| Newport 2015 | | Diagnosis: Bipolar Depression, Unipolar Depression  Resistant: Not reported in some of the RCTs  Suicidal: Not reported in some of the RCTs | Ketamine IV,Ketamine IN | Saline,Midazolam | NR | OR | Sample size: 168  Effect measure: 14.5 (2.7, 78.5)  p-value: 0.002 | Het.: I²: NA, p=NA  Pub.B. Test: NA, p=NA |
| Newport 2015 | | Diagnosis: Bipolar Depression, Unipolar Depression  Resistant: Yes  Suicidal: Not reported in some of the RCTs | Ketamine IV,Ketamine IN | Saline,Midazolam | NR | OR | Sample size: 141  Effect measure: 8.4 (1.6, 45)  p-value: 0.01 | Het.: I²: NA, p=NA  Pub.B. Test: NA, p=NA |
| Xu 2016 | | Diagnosis: Bipolar Depression, Unipolar Depression  Resistant: Not reported in some of the RCTs  Suicidal: Not reported in some of the RCTs | Ketamine IV,Ketamine IN | Saline,Midazolam | Random Effect | RR | Sample size: 168  Effect measure: 5.2 (2.1, 12.9)  p-value: 0.0003 | Het.: I²: NA, p=NA  Pub.B. Test: NA, p=NA |
| Zheng 2020 | | Diagnosis: Unipolar Depression  Resistant: No Information  Suicidal: No information | Esketamine IN | Saline | Random Effect | RR | Sample size: 199  Effect measure: 6.87 (1.55, 30.35)  p-value: 0.01 | Het.: I²: 44%, p=0.15  Pub.B. Test: NA, p=NA |
| **Efficacy – Remission**  **(3-6 days)** | | | | | | | | |
| Caddy 2015 | | Diagnosis: Unipolar Depression  Resistant: Yes  Suicidal: Not reported in all of the RCTs | Ketamine IV | ECT | NA - Single RCT | OR | Sample size: 18  Effect measure: 3.35 (0.12, 93.83)  p-value: 0.48 | Het.: I²: NA, p=NA  Pub.B. Test: NA, p=NA |
| Caddy 2015 | | Diagnosis: Unipolar Depression  Resistant: Yes  Suicidal: No | Ketamine IV | Midazolam | NA - Single RCT | OR | Sample size: 72  Effect measure: 1.88 (0.59, 5.96)  p-value: 0.29 | Het.: I²: NA, p=NA  Pub.B. Test: NA, p=NA |
| Caddy 2015 | | Diagnosis: Unipolar Depression  Resistant: Not reported in some of the RCTs  Suicidal: Not reported in some of the RCTs | Ketamine IV | Saline | Random Effect | OR | Sample size: 52  Effect measure: 6.69 (1.25, 35.71)  p-value: 0.026 | Het.: I²: 0%, p=0.92  Pub.B. Test: NA, p=NA |
| Dean 2021 | | Diagnosis: Bipolar Depression  Resistant: Yes  Suicidal: Not reported in some of the RCTs | Ketamine IV | Saline | Random Effect | OR | Sample size: 33  Effect measure: 3.62 (0.34, 38.6)  p-value: 0.29 | Het.: I²: 0%, p=0.95  Pub.B. Test: NA, p=NA |
| Han 2016 | | Diagnosis: Unipolar Depression  Resistant: Not reported in some of the RCTs  Suicidal: Not reported in some of the RCTs | Ketamine IV | Saline,Midazolam,ECT | Random Effect | OR | Sample size: 169  Effect measure: 4.04 (1.66, 9.85)  p-value: 0.002 | Het.: I²: 0%, p=0.94  Pub.B. Test: NA, p=NA |
| Kishimoto 2016 | | Diagnosis: Bipolar Depression, Unipolar Depression  Resistant: Yes  Suicidal: Not reported in some of the RCTs | Ketamine IV | Saline | Random Effect | RR | Sample size: 51  Effect measure: 5.22 (1.203, 22.641)  p-value: 0.027 | Het.: I²: 0%, p=0.88  Pub.B. Test: NA, p=NA |
| Kryst 2020 | | Diagnosis: Unipolar Depression, Bipolar Depression  Resistant: Not reported in some of the RCTs  Suicidal: Not reported in some of the RCTs | Ketamine IV | Saline | Random Effect | OR | Sample size: 112  Effect measure: 5.58 (1.59, 19.53)  p-value: 0.007 | Het.: I²: 0%, p=0.95  Pub.B. Test: NA, p=NA |
| McGirr 2015 | | Diagnosis: Bipolar Depression, Unipolar Depression  Resistant: Not reported in some of the RCTs  Suicidal: Not reported in some of the RCTs | Ketamine IV | Saline,Midazolam | Random Effect | OR | Sample size: 150  Effect measure: 3.87 (1.54, 9.75)  p-value: 0.004 | Het.: I²: NA, p=0.54  Pub.B. Test: Egger, Fail-safe N, p=0.100 |
| Newport 2015 | | Diagnosis: Unipolar Depression, Bipolar Depression  Resistant: Not reported in some of the RCTs  Suicidal: Not reported in some of the RCTs | Ketamine IV,Ketamine IN | Saline,Midazolam | NR | OR | Sample size: 175  Effect measure: 5.6 (1.2, 27.1)  p-value: 0.03 | Het.: I²: NA, p=NA  Pub.B. Test: NA, p=NA |
| **Efficacy – Remission**  **(7-13 days)** | | | | | | | | |
| Caddy 2015 | | Diagnosis: Unipolar Depression  Resistant: Yes  Suicidal: Not reported in all of the RCTs | Ketamine IV | ECT | NA - Single RCT | OR | Sample size: 18  Effect measure: 10.23 (0.45, 233.23)  p-value: 0.14 | Het.: I²: NA, p=NA  Pub.B. Test: NA, p=NA |
| Caddy 2015 | | Diagnosis: Unipolar Depression  Resistant: Yes  Suicidal: No | Ketamine IV | Midazolam | NA - Single RCT | OR | Sample size: 72  Effect measure: 1.37 (0.42, 4.46)  p-value: 0.6 | Het.: I²: NA, p=NA  Pub.B. Test: NA, p=NA |
| Dean 2021 | | Diagnosis: Bipolar Depression  Resistant: Yes  Suicidal: No | Ketamine IV | Saline | NA - Single RCT | OR | Sample size: 18  Effect measure: 3.35 (0.12, 93.83)  p-value: 0.48 | Het.: I²: NA, p=NA  Pub.B. Test: NA, p=NA |
| Han 2016 | | Diagnosis: Unipolar Depression  Resistant: Not reported in some of the RCTs  Suicidal: Not reported in some of the RCTs | Ketamine IV | Saline,Midazolam,ECT | Random Effect | OR | Sample size: 169  Effect measure: 4.6 (1.88, 11.26)  p-value: 0.0008 | Het.: I²: 0%, p=0.96  Pub.B. Test: NA, p=NA |
| Kishimoto 2016 | | Diagnosis: Bipolar Depression  Resistant: Yes  Suicidal: No | Ketamine IV | Saline | NA - Single RCT | RR | Sample size: 18  Effect measure: 1 (0.068, 14.786)  p-value: 1 | Het.: I²: NA, p=NA  Pub.B. Test: NA, p=NA |
| Kishimoto 2016 | | Diagnosis: Bipolar Depression, Unipolar Depression  Resistant: Yes  Suicidal: No | Ketamine IV | Saline | Random Effect | RR | Sample size: 36  Effect measure: 4.275 (0.706, 25.903)  p-value: 0.114 | Het.: I²: NA, p=NA  Pub.B. Test: NA, p=NA |
| Kryst 2020 | | Diagnosis: Bipolar Depression, Unipolar Depression  Resistant: Not reported in some of the RCTs  Suicidal: No | Ketamine IV | Saline | Random Effect | OR | Sample size: 90  Effect measure: 7.47 (1.75, 31.94)  p-value: 0.007 | Het.: I²: 0%, p=0.95  Pub.B. Test: NA, p=NA |
| Marcantoni 2020 | | Diagnosis: Unipolar Depression, Bipolar Depression  Resistant: Not reported in some of the RCTs  Suicidal: No | Ketamine IV | Saline,Midazolam | Random Effect | OR | Sample size: 135  Effect measure: 4.18 (0.71, 24.64)  p-value: 0.569 | Het.: I²: 0%, p=0.86  Pub.B. Test: Egger, Funnel Plots, Rosenthal's fail-safe N, p=0.0013 |
| McGirr 2015 | | Diagnosis: Bipolar Depression, Unipolar Depression  Resistant: Not reported in some of the RCTs  Suicidal: Not reported in some of the RCTs | Ketamine IV | Saline,Midazolam | Random Effect | OR | Sample size: 150  Effect measure: 4 (1.53, 10.51)  p-value: 0.005 | Het.: I²: NA, p=0.58  Pub.B. Test: Egger, Fail-safe N, p=0.750 |
| Newport 2015 | | Diagnosis: Bipolar Depression, Unipolar Depression  Resistant: Not reported in some of the RCTs  Suicidal: Not reported in some of the RCTs | Ketamine IV,Ketamine IN | Saline,Midazolam | NA | OR | Sample size: 168  Effect measure: 3.1 (0.6, 15.4)  p-value: 0.17 | Het.: I²: NA, p=NA  Pub.B. Test: NA, p=NA |
| Zheng 2020 | | Diagnosis: Unipolar Depression  Resistant: No Information  Suicidal: No information | Esketamine IN | Saline | Random Effect | RR | Sample size: 133  Effect measure: 4.66 (1.12, 19.42)  p-value: 0.03 | Het.: I²: 0%, p=NA  Pub.B. Test: NA, p=NA |
| **Efficacy – Remission**  **(14-28 days)** | | | | | | | | |
| Caddy 2015 | | Diagnosis: Unipolar Depression  Resistant: Yes  Suicidal: Not reported in all of the RCTs | Ketamine IV | ECT | NA - Single RCT | OR | Sample size: 18  Effect measure: 4 (0.33, 48.66)  p-value: 0.28 | Het.: I²: NA, p=NA  Pub.B. Test: NA, p=NA |
| Jawad 2022 | | Diagnosis: Unipolar Depression  Resistant: Not reported in some of the RCTs  Suicidal: Mixed | Esketamine IN | Saline | Random Effect | RR | Sample size: 855  Effect measure: 1.366 (1.182, 1.578)  p-value: 0.0001 | Het.: I²: NA, p=NA  Pub.B. Test: Egger, p=0.61 |
| Kishimoto 2016 | | Diagnosis: Bipolar Depression  Resistant: Yes  Suicidal: No | Ketamine IV | Saline | NA - Single RCT | RR | Sample size: 18  Effect measure: 3 (0.13, 69.09)  p-value: 0.492 | Het.: I²: NA, p=NA  Pub.B. Test: NA, p=NA |
| Newport 2015 | | Diagnosis: Unipolar Depression, Bipolar Depression  Resistant: Not reported in some of the RCTs  Suicidal: Not reported in some of the RCTs | Ketamine IV | Saline | NA | OR | Sample size: 58  Effect measure: 1.5 (0.3, 7.9)  p-value: 0.65 | Het.: I²: NA, p=NA  Pub.B. Test: NA, p=NA |
| Zheng 2020 | | Diagnosis: Unipolar Depression  Resistant: No Information  Suicidal: No information | Esketamine IN | Saline | Random Effect | RR | Sample size: 626  Effect measure: 1.38 (1.11, 1.72)  p-value: 0.004 | Het.: I²: 9%, p=0.33  Pub.B. Test: NA, p=NA |
| **Efficacy – Remission**  **(>28 days)** | | | | | | | | |
| An 2021 | | Diagnosis: Unipolar Depression  Resistant: Not reported in some of the RCTs  Suicidal: Mixed | Esketamine IN | Saline | Random Effect | RR | Sample size: 404  Effect measure: 1.7 (1.28, 2.24)  p-value: 0.001 | Het.: I²: 0%, p=0.587  Pub.B. Test: NA, p=NA |
| **Efficacy – Remission**  **(Endpoint)** | | | | | | | | |
| An 2021 | | Diagnosis: Unipolar Depression  Resistant: Not reported in some of the RCTs  Suicidal: Mixed | Esketamine IN | Saline | Random Effect | RR | Sample size: 404  Effect measure: 1.89 (1.45, 2.47)  p-value: Not reported | Het.: I²: 12.4%, p=0.335  Pub.B. Test: NA, p=NA |
| Bahji 2020 | | Diagnosis: Bipolar Depression  Resistant: Yes  Suicidal: Not reported in some of the RCTs | Ketamine IV | Saline | Random Effect | RR | Sample size: 33  Effect measure: 4.92 (1.07, 22.71)  p-value: Not reported | Het.: I²: 54.1%, p=0.1859  Pub.B. Test: Egger, p=NA |
| Hock 2022 | | Diagnosis: Unipolar Depression  Resistant: No Information  Suicidal: No information | Esketamine IN | Saline | Random Effect | RR | Sample size:  Effect measure: 1.37 (NA, NA)  p-value: 0.0001 | Het.: I²: %, p=0.7  Pub.B. Test: NA, p=NA |
| Marcantoni 2020 | | Diagnosis: Unipolar Depression, Bipolar Depression  Resistant: Not reported in some of the RCTs  Suicidal: No | Ketamine IV | Saline,Midazolam | Random Effect | OR | Sample size: 135  Effect measure: 5.11 (2.15, 12.17)  p-value: 0.003 | Het.: I²: 0%, p=0.86  Pub.B. Test: Egger, Funnel Plots, Rosenthal's fail-safe N, p=0.0013 |
| Nuñez 2020 | | Diagnosis: Unipolar Depression  Resistant: Not reported in some of the RCTs  Suicidal: Not reported in some of the RCTs | Ketamine Oral | Diclofenac,Saline | Random Effect | RR | Sample size: 161  Effect measure: 2.77 (0.96, 8)  p-value: 0.06 | Het.: I²: 35.89%, p=0.21  Pub.B. Test: NA, p=NA |
| Papakostas 2020 | | Diagnosis: Unipolar Depression  Resistant: Not reported in some of the RCTs  Suicidal: Mixed | Esketamine IN | Saline | Random Effect | RR | Sample size: 404  Effect measure: 1.45 (1.2, 1.75)  p-value: 0.0001 | Het.: I²: NA, p=NA  Pub.B. Test: Egger, p=0.132 |
| Zheng 2020 | | Diagnosis: Unipolar Depression  Resistant: Not reported in some of the RCTs  Suicidal: Mixed | Esketamine IN | Saline | Random Effect | RR | Sample size: 267  Effect measure: 1.42 (1.17, 1.72)  p-value: 0.0004 | Het.: I²: 0%, p=0.49  Pub.B. Test: NA, p=NA |
| **Efficacy – Response**  **(<60 mins)** | | | | | | | | |
| Kishimoto 2016 | | Diagnosis: Bipolar Depression, Unipolar Depression  Resistant: Yes  Suicidal: Not reported in some of the RCTs | Ketamine IV | Saline | Random Effect | RR | Sample size: 51  Effect measure: 13.626 (2.67, 69.546)  p-value: 0.002 | Het.: I²: 0%, p=0.73  Pub.B. Test: NA, p=NA |
| Newport 2015 | | Diagnosis: Bipolar Depression, Unipolar Depression  Resistant: Yes  Suicidal: Not reported in some of the RCTs | Ketamine IV,Ketamine IN | Saline | NR | OR | Sample size: 69  Effect measure: 13.2 (3.2, 53.7)  p-value: 0.001 | Het.: I²: NA, p=NA  Pub.B. Test: NA, p=NA |
| **Efficacy – Response**  **(60-90 mins)** | | | | | | | | |
| Kishimoto 2016 | | Diagnosis: Bipolar Depression, Unipolar Depression  Resistant: Yes  Suicidal: Not reported in some of the RCTs | Ketamine IV | Saline | Random Effect | RR | Sample size: 51  Effect measure: 10.547 (2.591, 42.935)  p-value: 0.001 | Het.: I²: NA, p=NA  Pub.B. Test: NA, p=NA |
| Newport_2015 | | Diagnosis: Bipolar Depression, Unipolar Depression  Resistant: Yes  Suicidal: Not reported in some of the RCTs | Ketamine IV | Saline | NR | OR | Sample size: 51  Effect measure: 24.7 (5, 122.5)  p-value: 0.001 | Het.: I²: NA, p=NA  Pub.B. Test: NA, p=NA |
| **Efficacy – Response**  **(90-120 mins)** | | | | | | | | |
| Kishimoto 2016 | | Diagnosis: Bipolar Depression, Unipolar Depression  Resistant: Yes  Suicidal: Not reported in some of the RCTs | Ketamine IV | Saline | Random Effect | RR | Sample size: 51  Effect measure: 13.77 (3.463, 54.753)  p-value: 0 | Het.: I²: NA, p=NA  Pub.B. Test: NA, p=NA |
| Newport 2015 | | Diagnosis: Bipolar Depression, Unipolar Depression  Resistant: Yes  Suicidal: Not reported in some of the RCTs | Ketamine IV | Saline | NR | OR | Sample size: 51  Effect measure: 24.7 (5, 122.5)  p-value: 0.001 | Het.: I²: NA, p=NA  Pub.B. Test: NA, p=NA |
| Zheng 2020 | | Diagnosis: Unipolar Depression  Resistant: No Information  Suicidal: No information | Esketamine IN | Saline | Random Effect | RR | Sample size: 133  Effect measure: 2.77 (1.62, 4.76)  p-value: 0.0002 | Het.: I²: 0%, p=0.78  Pub.B. Test: NA, p=NA |
| **Efficacy – Response**  **(120-240 mins)** | | | | | | | | |
| Kishimoto 2016 | | Diagnosis: Bipolar Depression, Unipolar Depression  Resistant: Yes  Suicidal: Not reported in some of the RCTs | Ketamine IV | Saline | Random Effect | RR | Sample size: 51  Effect measure: 14.721 (3.716, 58.317)  p-value: 0.001 | Het.: I²: 0%, p=0.91  Pub.B. Test: NA, p=NA |
| **Efficacy – Response**  **(24-48h)** | | | | | | | | |
| An 2021 | | Diagnosis: Unipolar Depression  Resistant: Yes  Suicidal: No | Ketamine IN,Esketamine IN | Saline | Random Effect | RR | Sample size: 219  Effect measure: 3.22 (1.85, 5.61)  p-value: 0.001 | Het.: I²: 58.8%, p=0.063  Pub.B. Test: NA, p=NA |
| Caddy 2015 | | Diagnosis: Unipolar Depression  Resistant: Yes  Suicidal: Not reported in all of the RCTs | Ketamine IV | ECT | NA - Single RCT | OR | Sample size: 18  Effect measure: 28 (2.07, 379.25)  p-value: 0.01 | Het.: I²: NA, p=NA  Pub.B. Test: NA, p=NA |
| Caddy 2015 | | Diagnosis: Unipolar Depression  Resistant: Yes  Suicidal: No | Ketamine IV | Midazolam | NA - Single RCT | RD | Sample size: 72  Effect measure: 0.36 (0.14, 0.58)  p-value: 0.002 | Het.: I²: 0%, p=NA  Pub.B. Test: NA, p=NA |
| Caddy 2015 | | Diagnosis: Unipolar Depression  Resistant: Not reported in some of the RCTs  Suicidal: Not reported in some of the RCTs | Ketamine IV | Saline | Random Effect | OR | Sample size: 52  Effect measure: 10.77 (2, 58)  p-value: 0.0057 | Het.: I²: 0%, p=0.46  Pub.B. Test: NA, p=NA |
| Dean 2021 | | Diagnosis: Bipolar Depression  Resistant: Not reported in all of the RCTs  Suicidal: Yes | Ketamine IV | Midazolam | NA - Single RCT | OR | Sample size: 16  Effect measure: 3.2 (0.23, 45.19)  p-value: 0.39 | Het.: I²: NA, p=NA  Pub.B. Test: NA, p=NA |
| Dean 2021 | | Diagnosis: Bipolar Depression  Resistant: Yes  Suicidal: Not reported in some of the RCTs | Ketamine IV | Saline | Random Effect | OR | Sample size: 33  Effect measure: 11.61 (1.25, 107.74)  p-value: 0.03 | Het.: I²: 0%, p=0.91  Pub.B. Test: NA, p=NA |
| Fornaro 2020 | | Diagnosis: Bipolar Depression  Resistant: Yes  Suicidal: Not reported in some of the RCTs | Ketamine IV | Saline | Random Effect | OR | Sample size: 33  Effect measure: 10.682 (2.142, 53.272)  p-value: 0.005 | Het.: I²: 0%, p=0.005  Pub.B. Test: Egger, Begg-Mazumdar Kendall's tau, p=NA |
| Han 2016 | | Diagnosis: Unipolar Depression  Resistant: Not reported in some of the RCTs  Suicidal: Not reported in some of the RCTs | Ketamine IV,Ketamine IN | Saline,ECT,Midazolam | Random Effect | OR | Sample size: 169  Effect measure: 10.09 (4.96, 20.52)  p-value: 0.00001 | Het.: I²: 0%, p=0.57  Pub.B. Test: Egger, p=NA |
| Kishimoto 2016 | | Diagnosis: Bipolar Depression, Unipolar Depression  Resistant: Not reported in some of the RCTs  Suicidal: Not reported in some of the RCTs | Ketamine IV | Saline,Midazolam | Random Effect | RR | Sample size: 161  Effect measure: 4.701 (2.225, 9.936)  p-value: 0 | Het.: I²: NA, p=NA  Pub.B. Test: NA, p=NA |
| Kishimoto 2016 | | Diagnosis: Bipolar Depression, Unipolar Depression  Resistant: Not reported in some of the RCTs  Suicidal: Not reported in some of the RCTs | Ketamine IV | Saline,Midazolam | Random Effect | RR | Sample size: 130  Effect measure: 4.632 (2.182, 9.83)  p-value: 0 | Het.: I²: NA, p=NA  Pub.B. Test: NA, p=NA |
| Kryst 2020 | | Diagnosis: No Information  Resistant: No Information  Suicidal: No Information | Ketamine | Placebo/Sham | Random Effect | OR | Sample size:  Effect measure: 5.64 (3.23, 9.85)  p-value: 0.00001 | Het.: I²: %, p=NA  Pub.B. Test: , p=NA |
| Kryst 2020 | | Diagnosis: Unipolar Depression, Bipolar Depression  Resistant: Not reported in some of the RCTs  Suicidal: Not reported in some of the RCTs | Ketamine IV | Saline,Midazolam | Random Effect | OR | Sample size: 418  Effect measure: 8.05 (4.24, 15.3)  p-value: 0.00001 | Het.: I²: 0%, p=0.95  Pub.B. Test: NA, p=NA |
| Marcantoni 2020 | | Diagnosis: Unipolar Depression, Bipolar Depression  Resistant: Not reported in some of the RCTs  Suicidal: Not reported in some of the RCTs | Ketamine IV | Saline,Midazolam | Random Effect | OR | Sample size: 314  Effect measure: 7.39 (2.5, 21.83)  p-value: 0.34 | Het.: I²: 12%, p=0.34  Pub.B. Test: Egger, Funnel Plots, Rosenthal's fail-safe N, p=0.0013 |
| McGirr 2015 | | Diagnosis: Unipolar Depression  Resistant: Yes  Suicidal: No | Ketamine IV | Midazolam | NA - Single RCT | OR | Sample size: 72  Effect measure: 4.29 (1.5, 12.25)  p-value: 0.007 | Het.: I²: NA, p=NA  Pub.B. Test: NA, p=NA |
| McGirr 2015 | | Diagnosis: Unipolar Depression, Bipolar Depression  Resistant: Not reported in some of the RCTs  Suicidal: Not reported in some of the RCTs | Ketamine IV,Ketamine IN | Saline | Random Effect | OR | Sample size: 103  Effect measure: 18.73 (6.39, 54.87)  p-value: 0.001 | Het.: I²: NA, p=NA  Pub.B. Test: NA, p=NA |
| McGirr 2015 | | Diagnosis: Unipolar Depression, Bipolar Depression  Resistant: Not reported in some of the RCTs  Suicidal: Not reported in some of the RCTs | Ketamine IV,Ketamine IN | Saline,Midazolam | Random Effect | OR | Sample size: 175  Effect measure: 8.81 (4.16, 18.68)  p-value: 0 | Het.: I²: NA, p=0.25  Pub.B. Test: Egger, Fail-safe N, p=0.050 |
| Newport 2015 | | Diagnosis: Bipolar Depression  Resistant: Yes  Suicidal: Not reported in some of the RCTs | Ketamine IV | Saline | NR | OR | Sample size: 33  Effect measure: 24.05 (2.96, 195.56)  p-value: 0.003 | Het.: I²: NA, p=NA  Pub.B. Test: NA, p=NA |
| Newport 2015 | | Diagnosis: Bipolar Depression, Unipolar Depression  Resistant: Yes  Suicidal: Not reported in some of the RCTs | Ketamine IV,Ketamine IN | Saline | NR | OR | Sample size: 69  Effect measure: 24.4 (6, 99.5)  p-value: 0.001 | Het.: I²: NA, p=NA  Pub.B. Test: NA, p=NA |
| Newport 2015 | | Diagnosis: Bipolar Depression, Unipolar Depression  Resistant: Not reported in some of the RCTs  Suicidal: Not reported in some of the RCTs | Ketamine IV,Ketamine IN | Saline,Midazolam | NR | OR | Sample size: 168  Effect measure: 9.865 (4.366, 22.293)  p-value: 0.001 | Het.: I²: 0%, p=0.51  Pub.B. Test: NA, p=NA |
| Newport 2015 | | Diagnosis: Bipolar Depression, Unipolar Depression  Resistant: Yes  Suicidal: Not reported in some of the RCTs | Ketamine IV,Ketamine IN | Saline,Midazolam | NR | OR | Sample size: 141  Effect measure: 8.4 (3.4, 20.4)  p-value: 0.001 | Het.: I²: NA, p=NA  Pub.B. Test: NA, p=NA |
| Newport 2015 | | Diagnosis: Unipolar Depression  Resistant: Not reported in some of the RCTs  Suicidal: Not reported in some of the RCTs | Ketamine IV,Ketamine IN | Saline,Midazolam | NR | OR | Sample size: 115  Effect measure: 7.55 (2.89, 19.76)  p-value: 0.001 | Het.: I²: NA, p=NA  Pub.B. Test: NA, p=NA |
| Newport 2015 | | Diagnosis: Unipolar Depression, Bipolar Depression  Resistant: Not reported in some of the RCTs  Suicidal: Not reported in some of the RCTs | Ketamine IN,Ketamine IV | Saline,Midazolam | NR | OR | Sample size: 130  Effect measure: 8.42 (3.47, 20.39)  p-value: 0.001 | Het.: I²: NA, p=NA  Pub.B. Test: NA, p=NA |
| Zheng 2020 | | Diagnosis: Unipolar Depression  Resistant: No Information  Suicidal: No information | Esketamine IN | Saline | Random Effect | RR | Sample size: 344  Effect measure: 5.42 (1.38, 21.2)  p-value: 0.02 | Het.: I²: 63%, p=0.04  Pub.B. Test: NA, p=NA |
| **Efficacy – Response**  **(3-6 days)** | | | | | | | | |
| Caddy 2015 | | Diagnosis: Unipolar Depression  Resistant: Yes  Suicidal: Not reported in all of the RCTs | Ketamine IV | ECT | NA - Single RCT | OR | Sample size: 18  Effect measure: 12.25 (1.33, 113.06)  p-value: 0.03 | Het.: I²: NA, p=NA  Pub.B. Test: NA, p=NA |
| Caddy 2015 | | Diagnosis: Unipolar Depression  Resistant: Yes  Suicidal: No | Ketamine IV | Midazolam | NA - Single RCT | RD | Sample size: 72  Effect measure: 0.37 (0.16, 0.59)  p-value: 0.0005 | Het.: I²: NA, p=NA  Pub.B. Test: NA, p=NA |
| Caddy 2015 | | Diagnosis: Unipolar Depression  Resistant: Not reported in some of the RCTs  Suicidal: Not reported in some of the RCTs | Ketamine IV | Saline | Random Effect | OR | Sample size: 52  Effect measure: 12.59 (2.38, 66.73)  p-value: 0.0029 | Het.: I²: 0%, p=0.89  Pub.B. Test: NA, p=NA |
| Dean 2021 | | Diagnosis: Bipolar Depression  Resistant: Yes  Suicidal: Not reported in some of the RCTs | Ketamine IV | Saline | Random Effect | OR | Sample size: 33  Effect measure: 8.24 (0.84, 80.61)  p-value: 0.07 | Het.: I²: 0%, p=0.55  Pub.B. Test: NA, p=NA |
| Han 2016 | | Diagnosis: Unipolar Depression  Resistant: Not reported in some of the RCTs  Suicidal: Not reported in some of the RCTs | Ketamine IV,Ketamine IN | Saline,ECT,Midazolam | Random Effect | OR | Sample size: 169  Effect measure: 7.42 (3.97, 13.88)  p-value: 0.00001 | Het.: I²: 0%, p=0.9  Pub.B. Test: Egger, p=NA |
| Kishimoto 2016 | | Diagnosis: Bipolar Depression, Unipolar Depression  Resistant: Not reported in some of the RCTs  Suicidal: Not reported in some of the RCTs | Ketamine IV | Saline,Midazolam | Random Effect | RR | Sample size: 157  Effect measure: 3.889 (2.196, 6.888)  p-value: 0 | Het.: I²: NA, p=NA  Pub.B. Test: NA, p=NA |
| Kryst 2020 | | Diagnosis: Unipolar Depression, Bipolar Depression  Resistant: Not reported in some of the RCTs  Suicidal: Not reported in some of the RCTs | Ketamine IV,Ketamine IN | Saline,Midazolam | Random Effect | OR | Sample size: 356  Effect measure: 5.13 (2.9, 9.05)  p-value: 0.00001 | Het.: I²: 0%, p=0.92  Pub.B. Test: NA, p=NA |
| McGirr 2015 | | Diagnosis: Unipolar Depression  Resistant: Yes  Suicidal: No | Ketamine IV | Midazolam | NA - Single RCT | OR | Sample size: 72  Effect measure: 4.83 (1.63, 14.3)  p-value: 0.004 | Het.: I²: NA, p=NA  Pub.B. Test: NA, p=NA |
| McGirr 2015 | | Diagnosis: Unipolar Depression, Bipolar Depression  Resistant: Not reported in some of the RCTs  Suicidal: Not reported in some of the RCTs | Ketamine IV,Ketamine IN | Saline | Random Effect | OR | Sample size: 103  Effect measure: 8.19 (3.36, 19.94)  p-value: 0 | Het.: I²: NA, p=NA  Pub.B. Test: NA, p=NA |
| McGirr 2015 | | Diagnosis: Unipolar Depression, Bipolar Depression  Resistant: Not reported in some of the RCTs  Suicidal: Not reported in some of the RCTs | Ketamine IV,Ketamine IN | Saline,Midazolam | Random Effect | OR | Sample size: 175  Effect measure: 6.63 (3.33, 13.18)  p-value: 0 | Het.: I²: NA, p=0.42  Pub.B. Test: Egger, Fail-safe N, p=0.170 |
| Newport 2015 | | Diagnosis: Unipolar Depression, Bipolar Depression  Resistant: Not reported in some of the RCTs  Suicidal: Not reported in some of the RCTs | Ketamine IV,Ketamine IN | Saline,Midazolam | NR | OR | Sample size: 175  Effect measure: 7.1 (3.3, 14.9)  p-value: 0.001 | Het.: I²: NA, p=NA  Pub.B. Test: NA, p=NA |
| **Efficacy – Response**  **(7-13 days)** | | | | | | | | |
| Caddy 2015 | | Diagnosis: Unipolar Depression  Resistant: Yes  Suicidal: Not reported in all of the RCTs | Ketamine IV | ECT | NA - Single RCT | OR | Sample size: 18  Effect measure: 3.35 (0.12, 93.83)  p-value: 0.48 | Het.: I²: NA, p=NA  Pub.B. Test: NA, p=NA |
| Caddy 2015 | | Diagnosis: Unipolar Depression  Resistant: Yes  Suicidal: No | Ketamine IV | Midazolam | NA - Single RCT | RD | Sample size: 72  Effect measure: 0.29 (0.08, 0.49)  p-value: 0.005 | Het.: I²: NA, p=NA  Pub.B. Test: NA, p=NA |
| Dean 2021 | | Diagnosis: Bipolar Depression  Resistant: Yes  Suicidal: No | Ketamine IV | Saline | NA - Single RCT | OR | Sample size: 18  Effect measure: 4 (0.33, 48.66)  p-value: 0.28 | Het.: I²: NA, p=NA  Pub.B. Test: NA, p=NA |
| Han 2016 | | Diagnosis: Unipolar Depression  Resistant: Not reported in some of the RCTs  Suicidal: Not reported in some of the RCTs | Ketamine IV,Ketamine IN | Saline,ECT,Midazolam | Random Effect | OR | Sample size: 169  Effect measure: 5.66 (2.92, 10.97)  p-value: 0.00001 | Het.: I²: 0%, p=0.9  Pub.B. Test: Egger, p=NA |
| Kishimoto 2016 | | Diagnosis: Bipolar Depression  Resistant: Yes  Suicidal: Not reported in some of the RCTs | Ketamine IV | Saline | Random Effect | RR | Sample size: 33  Effect measure: 1.597 (0.208, 12.288)  p-value: 0.653 | Het.: I²: NA, p=NA  Pub.B. Test: NA, p=NA |
| Kishimoto 2016 | | Diagnosis: Bipolar Depression, Unipolar Depression  Resistant: Not reported in some of the RCTs  Suicidal: Not reported in some of the RCTs | Ketamine IV | Saline,Midazolam | Random Effect | RR | Sample size: 150  Effect measure: 3.428 (1.771, 6.633)  p-value: 0.001 | Het.: I²: 0%, p=0.88  Pub.B. Test: NA, p=NA |
| Kryst 2020 | | Diagnosis: Unipolar Depression, Bipolar Depression  Resistant: Not reported in some of the RCTs  Suicidal: Not reported in some of the RCTs | Ketamine IV,Ketamine IN | Saline,Midazolam | Random Effect | OR | Sample size: 235  Effect measure: 5.71 (2.48, 13.16)  p-value: 0.0001 | Het.: I²: 0%, p=0.9  Pub.B. Test: NA, p=NA |
| Marcantoni 2020 | | Diagnosis: Unipolar Depression, Bipolar Depression  Resistant: Not reported in some of the RCTs  Suicidal: No | Ketamine IV | Saline,Midazolam | Random Effect | OR | Sample size: 135  Effect measure: 5.09 (1.88, 13.76)  p-value: 0.8 | Het.: I²: 0%, p=NA  Pub.B. Test: Egger, Funnel Plots, Rosenthal's fail-safe N, p=0.0013 |
| McGirr 2015 | | Diagnosis: Bipolar Depression, Unipolar Depression  Resistant: Not reported in some of the RCTs  Suicidal: Not reported in some of the RCTs | Ketamine IV,Ketamine IN | Saline | Random Effect | OR | Sample size: 96  Effect measure: 5.35 (1.97, 14.58)  p-value: 0.001 | Het.: I²: NA, p=NA  Pub.B. Test: NA, p=NA |
| McGirr 2015 | | Diagnosis: Bipolar Depression, Unipolar Depression  Resistant: Not reported in some of the RCTs  Suicidal: Not reported in some of the RCTs | Ketamine IV,Ketamine IN | Saline,Midazolam | Random Effect | OR | Sample size: 168  Effect measure: 4.8 (2.22, 10.38)  p-value: 0 | Het.: I²: NA, p=0.94  Pub.B. Test: Egger, Fail-safe N, p=0.530 |
| McGirr 2015 | | Diagnosis: Unipolar Depression  Resistant: Yes  Suicidal: No | Ketamine IV | Midazolam | NA - Single RCT | OR | Sample size: 72  Effect measure: 4.08 (1.22, 13.72)  p-value: 0.023 | Het.: I²: NA, p=NA  Pub.B. Test: NA, p=NA |
| Newport 2015 | | Diagnosis: Bipolar Depression, Unipolar Depression  Resistant: Not reported in some of the RCTs  Suicidal: Not reported in some of the RCTs | Ketamine IV | Saline,Midazolam | NR | OR | Sample size: 150  Effect measure: 4.58 (1.82, 11.49)  p-value: 0.001 | Het.: I²: NA, p=NA  Pub.B. Test: NA, p=NA |
| Newport 2015 | | Diagnosis: Bipolar Depression, Unipolar Depression  Resistant: Not reported in some of the RCTs  Suicidal: Not reported in some of the RCTs | Ketamine IV,Ketamine IN | Saline,Midazolam | NR | OR | Sample size: 168  Effect measure: 4.61 (2.076, 10.236)  p-value: 0 | Het.: I²: 0%, p=0.95  Pub.B. Test: NA, p=NA |
| Newport 2015 | | Diagnosis: Unipolar Depression  Resistant: Yes  Suicidal: No | Ketamine IN | Saline | NA - Single RCT | OR | Sample size: 18  Effect measure: 4.71 (0.95, 23.3)  p-value: 0.058 | Het.: I²: NA, p=NA  Pub.B. Test: NA, p=NA |
| Zheng 2020 | | Diagnosis: Unipolar Depression  Resistant: No Information  Suicidal: No information | Esketamine IN | Saline | Random Effect | RR | Sample size: 133  Effect measure: 3.87 (1.37, 10.93)  p-value: 0.01 | Het.: I²: 0%, p=NA  Pub.B. Test: NA, p=NA |
| **Efficacy – Response**  **(14-28 days)** | | | | | | | | |
| An 2021 | | Diagnosis: Unipolar Depression  Resistant: Yes  Suicidal: No | Esketamine IN | Saline | Random Effect | RR | Sample size: 338  Effect measure: 1.48 (1.17, 1.86)  p-value: 0.001 | Het.: I²: 0%, p=0.334  Pub.B. Test: NA, p=NA |
| Caddy 2015 | | Diagnosis: Unipolar Depression  Resistant: Yes  Suicidal: Not reported in all of the RCTs | Ketamine IV | ECT | NA - Single RCT | OR | Sample size: 18  Effect measure: 3.35 (0.12, 93.83)  p-value: 0.48 | Het.: I²: NA, p=NA  Pub.B. Test: NA, p=NA |
| Jawad 2022 | | Diagnosis: Unipolar Depression  Resistant: Not reported in some of the RCTs  Suicidal: Mixed | Esketamine IN | Saline | Random Effect | RR | Sample size: 631  Effect measure: 1.221 (1.055, 1.428)  p-value: 0.017 | Het.: I²: NA, p=NA  Pub.B. Test: Egger, p=0.37 |
| Kishimoto 2016 | | Diagnosis: Bipolar Depression  Resistant: Yes  Suicidal: Not reported in some of the RCTs | Ketamine IV | Saline | Random Effect | RR | Sample size: 33  Effect measure: 3.932 (0.457, 33.802)  p-value: 0.212 | Het.: I²: NA, p=NA  Pub.B. Test: NA, p=NA |
| Newport 2015 | | Diagnosis: Unipolar Depression, Bipolar Depression  Resistant: Not reported in some of the RCTs  Suicidal: Not reported in some of the RCTs | Ketamine IV | Saline | NR | OR | Sample size: 58  Effect measure: 4.4 (1, 18.8)  p-value: 0.05 | Het.: I²: NA, p=NA  Pub.B. Test: NA, p=NA |
| Papadimitropoulou 2017 | | Diagnosis: Unipolar Depression  Resistant: Yes  Suicidal: No | Ketamine IV | Aripiprazole augmentation | Random Effect | OR | Sample size: NA  Effect measure: 5.2 (1.4, 27.5)  p-value: Not reported | Het.: I²: NA, p=NA  Pub.B. Test: NA, p=NA |
| Papadimitropoulou 2017 | | Diagnosis: Unipolar Depression  Resistant: Yes  Suicidal: No | Ketamine IV | rTMS (80-120%) | Random Effect | OR | Sample size: NA  Effect measure: 4.8 (1.1, 27.5)  p-value: Not reported | Het.: I²: NA, p=NA  Pub.B. Test: NA, p=NA |
| Papadimitropoulou 2017 | | Diagnosis: Unipolar Depression  Resistant: Yes  Suicidal: No | Ketamine IV | Saline | Random Effect | OR | Sample size: NA  Effect measure: 13.7 (3.8, 69.1)  p-value: Not reported | Het.: I²: NA, p=NA  Pub.B. Test: NA, p=NA |
| Zheng 2020 | | Diagnosis: Unipolar Depression  Resistant: No Information  Suicidal: No information | Esketamine IN | Saline | Random Effect | RR | Sample size: 678  Effect measure: 3.17 (1.4, 7.18)  p-value: 0.006 | Het.: I²: 15%, p=0.31  Pub.B. Test: NA, p=NA |
| Zheng 2020 | | Diagnosis: Unipolar Depression  Resistant: No Information  Suicidal: No information | Esketamine IN | Saline | Random Effect | RR | Sample size: 626  Effect measure: 1.36 (1.16, 1.58)  p-value: 0.0001 | Het.: I²: 0%, p=0.97  Pub.B. Test: NA, p=NA |
| **Efficacy – Response**  **(Endpoint)** | | | | | | | | |
| An 2021 | | Diagnosis: Unipolar Depression  Resistant: Yes  Suicidal: No | Ketamine IN,Esketamine IN | Saline | Random Effect | RR | Sample size: 356  Effect measure: 1.85 (1.48, 2.32)  p-value: Not reported | Het.: I²: 63.7%, p=0.017  Pub.B. Test: NA, p=NA |
| Bahji 2020 | | Diagnosis: Bipolar Depression  Resistant: Yes  Suicidal: Not reported in some of the RCTs | Ketamine IV | Saline | Random Effect | RR | Sample size: 33  Effect measure: 12.49 (3.06, 50.93)  p-value: 0.01 | Het.: I²: 56.3%, p=0.0441  Pub.B. Test: Egger, p=NA |
| Marcantoni 2020 | | Diagnosis: Bipolar Depression, Unipolar Depression  Resistant: Not reported in some of the RCTs  Suicidal: Not reported in some of the RCTs | Ketamine IV | Saline,Midazolam | Random Effect | OR | Sample size: 314  Effect measure: 6.33 (3.33, 12.05)  p-value: 0.0001 | Het.: I²: 0%, p=0.64  Pub.B. Test: Egger, Rosenthal's fail-safe N, Funnel Plots, p=0.0013 |
| Nuñez 2020 | | Diagnosis: Unipolar Depression  Resistant: Not reported in some of the RCTs  Suicidal: Not reported in some of the RCTs | Ketamine Oral | Saline,Diclofenac | Random Effect | RR | Sample size: 161  Effect measure: 2.58 (0.94, 7.08)  p-value: 0.06558 | Het.: I²: 65.85%, p=0.0535  Pub.B. Test: NA, p=NA |
| Papakostas 2020 | | Diagnosis: Unipolar Depression  Resistant: Not reported in some of the RCTs  Suicidal: Mixed | Esketamine IN | Saline | Random Effect | RR | Sample size: 404  Effect measure: 1.4 (1.22, 1.61)  p-value: 0.0001 | Het.: I²: NA, p=NA  Pub.B. Test: Egger, p=0.087 |
| Zheng 2020 | | Diagnosis: Unipolar Depression  Resistant: Yes  Suicidal: No | Esketamine IN | Saline | Random Effect | RR | Sample size: 201  Effect measure: 1.39 (1.18, 1.64)  p-value: 0.0001 | Het.: I²: 5%, p=0.39  Pub.B. Test: NA, p=NA |
| **Efficacy – Suicide scales**  **(<60 mins)** | | | | | | | | |
| Witt 2020 | | Diagnosis: Bipolar Depression, Unipolar Depression  Resistant: Not reported in all of the RCTs  Suicidal: Mixed | Ketamine IV | Midazolam,Saline | Random Effect | SMD (Not Specified) | Sample size: 123  Effect measure: -0.21 (-0.58, 0.16)  p-value: 0.27 | Het.: I²: 3%, p=0.36  Pub.B. Test: NA, p=NA |
| **Efficacy – Suicide scales**  **(120-240 mins)** | | | | | | | | |
| Xiong 2021 | | Diagnosis: Bipolar Depression, Unipolar Depression  Resistant: Not reported in some of the RCTs  Suicidal: Mixed | Ketamine IV | Midazolam,Saline | Random Effect | Hedge's g | Sample size: 149  Effect measure: -1.166 (-1.923, -0.409)  p-value: 0.003 | Het.: I²: 60.56%, p=0.013  Pub.B. Test: Funnel Plots, p=NA |
| Xiong 2021 | | Diagnosis: Unipolar Depression  Resistant: Not reported in all of the RCTs  Suicidal: Yes | Esketamine IN | Saline | NA - Single RCT | Hedge's g | Sample size: 66  Effect measure: 1.023 (0.615, 1.432)  p-value: 0.001 | Het.: I²: NA, p=NA  Pub.B. Test: Funnel Plots, p=NA |
| Xiong 2021 | | Diagnosis: Unipolar Depression  Resistant: Not reported in some of the RCTs  Suicidal: Not reported in some of the RCTs | Ketamine IV | Saline,Midazolam,None | Random Effect | Hedge's g | Sample size: 77  Effect measure: 1.633 (0.802, 2.465)  p-value: 0.001 | Het.: I²: 60.56%, p=0.013  Pub.B. Test: Funnel Plots, p=NA |
| Xiong 2021 | | Diagnosis: Unipolar Depression, Bipolar Depression  Resistant: Not reported in some of the RCTs  Suicidal: Mixed | Esketamine IN,Ketamine IV | Saline,Midazolam | Random Effect | Hedge's g | Sample size: 215  Effect measure: -1.096 (-1.617, -0.576)  p-value: 0.001 | Het.: I²: 60.56%, p=0.013  Pub.B. Test: Funnel Plots, p=NA |
| **Efficacy – Suicide scales**  **(24-48h)** | | | | | | | | |
| Dean 2021 | | Diagnosis: Bipolar Depression  Resistant: Not reported in all of the RCTs  Suicidal: Yes | Ketamine IV | Midazolam | NA - Single RCT | MD | Sample size: 16  Effect measure: -5.86 (-15.76, 4.04)  p-value: 0.25 | Het.: I²: NA, p=NA  Pub.B. Test: NA, p=NA |
| Xiong 2021 | | Diagnosis: Bipolar Depression, Unipolar Depression  Resistant: Not reported in some of the RCTs  Suicidal: Not reported in some of the RCTs | Ketamine IV | Midazolam,Saline | Random Effect | Hedge's g | Sample size: 293  Effect measure: -1.035 (-1.277, 0.793)  p-value: 0.001 | Het.: I²: 60.56%, p=0.013  Pub.B. Test: Funnel Plots, p=NA |
| Xiong 2021 | | Diagnosis: Unipolar Depression  Resistant: Not reported in all of the RCTs  Suicidal: Yes | Esketamine IN | Saline | NA - Single RCT | Hedge's g | Sample size: 66  Effect measure: -1.309 (-1.761, -0.857)  p-value: 0.001 | Het.: I²: NA, p=NA  Pub.B. Test: Funnel Plots, p=NA |
| Xiong 2021 | | Diagnosis: Unipolar Depression, Bipolar Depression  Resistant: Not reported in some of the RCTs  Suicidal: Not reported in some of the RCTs | Esketamine IN,Ketamine IV | Saline,Midazolam | Random Effect | Hedge's g | Sample size: 359  Effect measure: -1.08 (-1.3, 0.86)  p-value: 0.001 | Het.: I²: 60.56%, p=0.013  Pub.B. Test: Funnel Plots, p=NA |
| **Efficacy – Suicide scales**  **(3-6 days)** | | | | | | | | |
| Xu 2016 | | Diagnosis: Bipolar Depression, Unipolar Depression  Resistant: Not reported in some of the RCTs  Suicidal: Not reported in some of the RCTs | Ketamine IV,Ketamine IN | Saline | Random Effect | SMD (Not Specified) | Sample size: 100  Effect measure: -0.4 (-0.7, -0.1)  p-value: Not reported | Het.: I²: 2%, p=NA  Pub.B. Test: NA, p=NA |
| **Efficacy – Suicide scales**  **(14-28 days)** | | | | | | | | |
| Witt 2020 | | Diagnosis: Bipolar Depression, Unipolar Depression  Resistant: Not reported in some of the RCTs  Suicidal: Not reported in some of the RCTs | Ketamine IV | Midazolam,Saline | Random Effect | SMD (Not Specified) | Sample size: 123  Effect measure: -0.24 (-0.53, 0.05)  p-value: 0.1 | Het.: I²: 0%, p=0.7  Pub.B. Test: NA, p=NA |
| **Efficacy – Suicide scales**  **(Endpoint)** | | | | | | | | |
| Caddy 2015 | | Diagnosis: Unipolar Depression  Resistant: Yes  Suicidal: No | Ketamine IV | Midazolam | NA - Single RCT | MD | Sample size: 72  Effect measure: -1.32 (-2.52, -0.12)  p-value: 0.03 | Het.: I²: NA, p=NA  Pub.B. Test: NA, p=NA |
| Xiong 2021 | | Diagnosis: Bipolar Depression, Unipolar Depression  Resistant: Not reported in some of the RCTs  Suicidal: Not reported in some of the RCTs | Ketamine IV,Esketamine IN | Midazolam,Saline,None | Random Effect | Hedge's g | Sample size: 346  Effect measure: -1.029 (-1.31, -0.75)  p-value: 0.001 | Het.: I²: NA, p=NA  Pub.B. Test: Funnel Plots, p=NA |
| **Tolerability – Adverse events (Total) (Endpoint)** | | | | | | | | |
| Nuñez 2020 | | Diagnosis: Unipolar Depression  Resistant: Not reported in some of the RCTs  Suicidal: Not reported in some of the RCTs | Ketamine Oral | Diclofenac,Saline | Random Effect | RR | Sample size: 161  Effect measure: 1.28 (0.89, 1.83)  p-value: 0.1895 | Het.: I²: 0%, p=0.423  Pub.B. Test: NA, p=NA |
| **Tolerability – Anxiety**  **(14-28 days)** | | | | | | | | |
| Jawad 2022 | | Diagnosis: Unipolar Depression  Resistant: No Information  Suicidal: No information | Esketamine IN | Saline | Pooled RR | RR | Sample size: NA  Effect measure: 1.406 (0.905, 2.183)  p-value: 0.129 | Het.: I²: NA, p=NA  Pub.B. Test: NA, p=NA |
| **Tolerability – Anxiety**  **(>28 days)** | | | | | | | | |
| Jawad 2022 | | Diagnosis: Unipolar Depression  Resistant: Yes  Suicidal: No | Esketamine IN | Saline | NA - Single RCT | RR | Sample size: NA  Effect measure: 2.289 (0.827, 6.337)  p-value: 0.111 | Het.: I²: NA, p=NA  Pub.B. Test: NA, p=NA |
| **Tolerability – Anxiety**  **(Endpoint)** | | | | | | | | |
| Bahji 2022 | | Diagnosis: No Information  Resistant: No Information  Suicidal: No information | No Information | Saline,Midazolam | Random Effect | OR | Sample size: NA  Effect measure: 1.67 (1, 2.77)  p-value: Not reported | Het.: I²: 1%, p=NA  Pub.B. Test: Egger, p=NA |
| Zheng 2020 | | Diagnosis: Unipolar Depression  Resistant: No Information  Suicidal: No information | Esketamine IN | Saline | Random Effect | RR | Sample size: 746  Effect measure: 1.7 (1, 2.91)  p-value: 0.05 | Het.: I²: 0%, p=NA  Pub.B. Test: NA, p=NA |
| **Tolerability – Anxiety/Agitation**  **(24-48h)** | | | | | | | | |
| Caddy 2015 | | Diagnosis: Unipolar Depression  Resistant: Yes  Suicidal: No | Ketamine IV | Midazolam | NA - Single RCT | OR | Sample size: 72  Effect measure: 1.99 (0.69, 5.75)  p-value: Not reported | Het.: I²: NA, p=NA  Pub.B. Test: NA, p=NA |
| **Tolerability – Anxiety/Agitation**  **(7-13 days)** | | | | | | | | |
| Caddy 2015 | | Diagnosis: Unipolar Depression  Resistant: Yes  Suicidal: No | Ketamine IV | Midazolam | NA - Single RCT | OR | Sample size: 72  Effect measure: 1.39 (0.66, 2.94)  p-value: Not reported | Het.: I²: NA, p=NA  Pub.B. Test: NA, p=NA |
| **Tolerability – Blood pressure increase**  **(Endpoint)** | | | | | | | | |
| Zheng 2020 | | Diagnosis: Unipolar Depression  Resistant: No Information  Suicidal: No information | Esketamine IN | Saline | Random Effect | RR | Sample size: 680  Effect measure: 2.46 (0.87, 6.97)  p-value: 0.09 | Het.: I²: 42%, p=NA  Pub.B. Test: NA, p=NA |
| **Tolerability – Blurred vision**  **(24-48h)** | | | | | | | | |
| Caddy 2015 | | Diagnosis: Unipolar Depression  Resistant: Yes  Suicidal: No | Ketamine IV | Midazolam | NA - Single RCT | OR | Sample size: 72  Effect measure: 8.52 (1.8, 40.39)  p-value: Not reported | Het.: I²: NA, p=NA  Pub.B. Test: NA, p=NA |
| **Tolerability – Blurred vision**  **(7-13 days)** | | | | | | | | |
| Caddy 2015 | | Diagnosis: Unipolar Depression  Resistant: Yes  Suicidal: No | Ketamine IV | Midazolam | NA - Single RCT | OR | Sample size: 72  Effect measure: 1.37 (0.25, 7.62)  p-value: | Het.: I²: NA, p=NA  Pub.B. Test: NA, p=NA |
| **Tolerability – Blurred vision**  **(14-28 days)** | | | | | | | | |
| Jawad 2022 | | Diagnosis: Unipolar Depression  Resistant: No Information  Suicidal: No information | Esketamine IN | Saline | Pooled RR | RR | Sample size: NA  Effect measure: 2.393 (1.456, 3.932)  p-value: 0.001 | Het.: I²: NA, p=NA  Pub.B. Test: NA, p=NA |
| **Tolerability – Blurred vision**  **(>28 days)** | | | | | | | | |
| Jawad 2022 | | Diagnosis: Unipolar Depression  Resistant: Yes  Suicidal: No | Esketamine IN | Saline | NA - Single RCT | RR | Sample size: NA  Effect measure: 22.895 (3.138, 167.052)  p-value: 0.002 | Het.: I²: NA, p=NA  Pub.B. Test: NA, p=NA |
| **Tolerability – Blurred vision**  **(Endpoint)** | | | | | | | | |
| Zheng 2020 | | Diagnosis: Unipolar Depression  Resistant: No Information  Suicidal: No information | Esketamine IN | Saline | Random Effect | RR | Sample size: 730  Effect measure: 6.73 (2.52, 18)  p-value: 0.0001 | Het.: I²: 0%, p=NA  Pub.B. Test: NA, p=NA |
| **Tolerability – BPRS**  **(<60 mins)** | | | | | | | | |
| Kishimoto 2016 | | Diagnosis: Unipolar Depression, Bipolar Depression  Resistant: Not reported in some of the RCTs  Suicidal: Not reported in some of the RCTs | Ketamine IV | Saline,Midazolam | Random Effect | Hedge's g | Sample size: 130  Effect measure: 0.898 (0.575, 1.221)  p-value: 0 | Het.: I²: 10.8%, p=0.35  Pub.B. Test: NA, p=NA |
| McGirr 2015 | | Diagnosis: Unipolar Depression, Bipolar Depression  Resistant: Not reported in some of the RCTs  Suicidal: Not reported in some of the RCTs | Ketamine IV,Ketamine IN | Saline,Midazolam | Random Effect | Rosenthal's r | Sample size: 175  Effect measure: -1.438 (-2.07, -0.805)  p-value: 0.001 | Het.: I²: NA, p=NA  Pub.B. Test: NA, p=NA |
| Romeo 2015 | | Diagnosis: Unipolar Depression, Bipolar Depression  Resistant: Not reported in some of the RCTs  Suicidal: Not reported in some of the RCTs | Ketamine IV,Ketamine IN | Saline | Random Effect | Cohen's d | Sample size: 103  Effect measure: 1.08 (0.62, 1.55)  p-value: 0.00001 | Het.: I²: 56%, p=0.05  Pub.B. Test: NA, p=NA |
| **Tolerability – BPRS**  **(60-90 mins)** | | | | | | | | |
| Kishimoto 2016 | | Diagnosis: Unipolar Depression, Bipolar Depression  Resistant: Not reported in some of the RCTs  Suicidal: Not reported in some of the RCTs | Ketamine IV | Saline | Random Effect | Hedge's g | Sample size: 58  Effect measure: -0.071 (-0.595, 0.454)  p-value: 0.792 | Het.: I²: NA, p=NA  Pub.B. Test: NA, p=NA |
| Romeo 2015 | | Diagnosis: No Information  Resistant: No Information  Suicidal: No Information | No Information | Placebo/Sham | Random Effect | Cohen's d | Sample size: NA  Effect measure: 0.06 (-0.37, 0.49)  p-value: 0.78 | Het.: I²: 25%, p=0.26  Pub.B. Test: NA, p=NA |
| **Tolerability – BPRS**  **(90-120 mins)** | | | | | | | | |
| Kishimoto 2016 | | Diagnosis: Unipolar Depression, Bipolar Depression  Resistant: Not reported in some of the RCTs  Suicidal: Not reported in some of the RCTs | Ketamine IV | Saline | Random Effect | Hedge's g | Sample size: 58  Effect measure: -0.1 (-0.456, 0.256)  p-value: 0.581 | Het.: I²: NA, p=NA  Pub.B. Test: NA, p=NA |
| **Tolerability – BPRS**  **(120-240 mins)** | | | | | | | | |
| Kishimoto 2016 | | Diagnosis: Unipolar Depression, Bipolar Depression  Resistant: Not reported in some of the RCTs  Suicidal: Not reported in some of the RCTs | Ketamine IV | Saline | Random Effect | Hedge's g | Sample size: 58  Effect measure: 0.007 (-0.347, 0.361)  p-value: 0.971 | Het.: I²: NA, p=NA  Pub.B. Test: NA, p=NA |
| **Tolerability – BPRS**  **(24-48h)** | | | | | | | | |
| Kishimoto 2016 | | Diagnosis: Bipolar Depression, Unipolar Depression  Resistant: Yes  Suicidal: Not reported in some of the RCTs | Ketamine IV | Saline | Random Effect | Hedge's g | Sample size: 51  Effect measure: -0.234 (-0.615, 0.146)  p-value: 0.228 | Het.: I²: NA, p=NA  Pub.B. Test: NA, p=NA |
| **Tolerability – BPRS**  **(3-6 days)** | | | | | | | | |
| Kishimoto 2016 | | Diagnosis: Bipolar Depression, Unipolar Depression  Resistant: Yes  Suicidal: Not reported in some of the RCTs | Ketamine IV | Saline | Random Effect | Hedge's g | Sample size: 51  Effect measure: -0.479 (-0.864, -0.094)  p-value: 0.015 | Het.: I²: 0%, p=0.89  Pub.B. Test: NA, p=NA |
| **Tolerability – BPRS**  **(7-13 days)** | | | | | | | | |
| Kishimoto 2016 | | Diagnosis: Bipolar Depression, Unipolar Depression  Resistant: Yes  Suicidal: Not reported in some of the RCTs | Ketamine IV | Saline | Random Effect | Hedge's g | Sample size: 51  Effect measure: -0.334 (-0.717, 0.05)  p-value: 0.088 | Het.: I²: NA, p=NA  Pub.B. Test: NA, p=NA |
| **Tolerability – BPRS**  **(14-28 days)** | | | | | | | | |
| Kishimoto 2016 | | Diagnosis: Bipolar Depression  Resistant: Yes  Suicidal: Not reported in some of the RCTs | Ketamine IV | Saline | Random Effect | Hedge's g | Sample size: 33  Effect measure: -0.206 (-1.054, 0.641)  p-value: 0.633 | Het.: I²: NA, p=NA  Pub.B. Test: NA, p=NA |
| **Tolerability – BPRS**  **(Endpoint)** | | | | | | | | |
| Newport 2015 | | Diagnosis: Bipolar Depression, Unipolar Depression  Resistant: Not reported in some of the RCTs  Suicidal: Not reported in some of the RCTs | Ketamine IV | Saline,Midazolam | NR | Hedge's g | Sample size: 130  Effect measure: 0.82 (NA, NA)  p-value: 0.001 | Het.: I²: NA, p=NA  Pub.B. Test: NA, p=NA |
| **Tolerability – CADSS**  **(<60 mins)** | | | | | | | | |
| Kishimoto 2016 | | Diagnosis: Bipolar Depression, Unipolar Depression  Resistant: Yes  Suicidal: Not reported in some of the RCTs | Ketamine IV | Saline,Midazolam | Random Effect | Hedge's g | Sample size: 105  Effect measure: 2.429 (1.125, 3.734)  p-value: 0.001 | Het.: I²: 92.3%, p=0.001  Pub.B. Test: NA, p=NA |
| McGirr 2015 | | Diagnosis: Bipolar Depression, Unipolar Depression  Resistant: Yes  Suicidal: Not reported in some of the RCTs | Ketamine IV,Ketamine IN | Saline,Midazolam | Random Effect | Rosenthal's r | Sample size: 123  Effect measure: -3.702 (-5.912, -1.492)  p-value: 0.001 | Het.: I²: NA, p=NA  Pub.B. Test: NA, p=NA |
| **Tolerability – CADSS**  **(60-90 mins)** | | | | | | | | |
| Kishimoto 2016 | | Diagnosis: Bipolar Depression  Resistant: Yes  Suicidal: Not reported in some of the RCTs | Ketamine IV | Saline | Random Effect | Hedge's g | Sample size: 33  Effect measure: 0.279 (-0.195, 0.753)  p-value: 0.248 | Het.: I²: NA, p=NA  Pub.B. Test: NA, p=NA |
| **Tolerability – CADSS**  **(90-120 mins)** | | | | | | | | |
| Kishimoto 2016 | | Diagnosis: Bipolar Depression  Resistant: Yes  Suicidal: Not reported in some of the RCTs | Ketamine IV | Saline | Random Effect | Hedge's g | Sample size: 33  Effect measure: -0.005 (-0.492, 0.481)  p-value: 0.982 | Het.: I²: NA, p=NA  Pub.B. Test: NA, p=NA |
| **Tolerability – CADSS**  **(120-240 mins)** | | | | | | | | |
| Kishimoto 2016 | | Diagnosis: Bipolar Depression  Resistant: Yes  Suicidal: Not reported in some of the RCTs | Ketamine IV | Saline | Random Effect | Hedge's g | Sample size: 33  Effect measure: -0.05 (-0.632, 0.532)  p-value: 0.867 | Het.: I²: NA, p=NA  Pub.B. Test: NA, p=NA |
| **Tolerability – CADSS**  **(24-48h)** | | | | | | | | |
| Kishimoto 2016 | | Diagnosis: Bipolar Depression  Resistant: Yes  Suicidal: Not reported in some of the RCTs | Ketamine IV | Saline | Random Effect | Hedge's g | Sample size: 33  Effect measure: -0.017 (-0.571, 0.536)  p-value: 0.951 | Het.: I²: NA, p=NA  Pub.B. Test: NA, p=NA |
| **Tolerability – CADSS**  **(3-6 days)** | | | | | | | | |
| Kishimoto 2016 | | Diagnosis: Bipolar Depression  Resistant: Yes  Suicidal: Not reported in some of the RCTs | Ketamine IV | Saline | Random Effect | Hedge's g | Sample size: 33  Effect measure: -0.055 (-0.688, 0.578)  p-value: 0.865 | Het.: I²: NA, p=NA  Pub.B. Test: NA, p=NA |
| **Tolerability – CADSS**  **(7-13 days)** | | | | | | | | |
| Kishimoto 2016 | | Diagnosis: Bipolar Depression  Resistant: Yes  Suicidal: Not reported in some of the RCTs | Ketamine IV | Saline | Random Effect | Hedge's g | Sample size: 33  Effect measure: -0.044 (-0.563, 0.474)  p-value: 0.867 | Het.: I²: NA, p=NA  Pub.B. Test: NA, p=NA |
| **Tolerability – CADSS**  **(14-28 days)** | | | | | | | | |
| Kishimoto 2016 | | Diagnosis: Bipolar Depression  Resistant: Yes  Suicidal: Not reported in some of the RCTs | Ketamine IV | Saline | Random Effect | Hedge's g | Sample size: 33  Effect measure: -0.1 (-0.631, 0.43)  p-value: 0.711 | Het.: I²: NA, p=NA  Pub.B. Test: NA, p=NA |
| **Tolerability – CADSS**  **(Endpoint)** | | | | | | | | |
| Newport 2015 | | Diagnosis: Bipolar Depression, Unipolar Depression  Resistant: Yes  Suicidal: Not reported in some of the RCTs | Ketamine IV | Saline,Midazolam | NR | Hedge's g | Sample size: 105  Effect measure: 1.78 (22.13, 25.37)  p-value: 0.001 | Het.: I²: NA, p=NA  Pub.B. Test: NA, p=NA |
| **Tolerability – Change in blood pressure**  **(Endpoint)** | | | | | | | | |
| Caddy 2015 | | Diagnosis: Unipolar Depression  Resistant: Not reported in all of the RCTs  Suicidal: No | Ketamine IV | Saline | NA - Single RCT | OR | Sample size: 27  Effect measure: 16.06 (0.74, 346.19)  p-value: Not reported | Het.: I²: NA, p=NA  Pub.B. Test: NA, p=NA |
| **Tolerability – Chest pain**  **(24-48h)** | | | | | | | | |
| Caddy 2015 | | Diagnosis: Unipolar Depression  Resistant: Yes  Suicidal: No | Ketamine IV | Midazolam | NA - Single RCT | OR | Sample size: 72  Effect measure: 2.8 (0.13, 60.66)  p-value: Not reported | Het.: I²: NA, p=NA  Pub.B. Test: NA, p=NA |
| **Tolerability – Chest pain**  **(7-13 days)** | | | | | | | | |
| Caddy 2015 | | Diagnosis: Unipolar Depression  Resistant: Yes  Suicidal: No | Ketamine IV | Midazolam | NA - Single RCT | OR | Sample size: 72  Effect measure: 0.51 (0.07, 3.87)  p-value: Not reported | Het.: I²: NA, p=NA  Pub.B. Test: NA, p=NA |
| **Tolerability – Confusion**  **(>28 days)** | | | | | | | | |
| Jawad 2022 | | Diagnosis: Unipolar Depression  Resistant: Yes  Suicidal: No | Esketamine IN | Saline | NA - Single RCT | RR | Sample size: NA  Effect measure: 18.131 (1.065, 308.699)  p-value: 0.045 | Het.: I²: NA, p=NA  Pub.B. Test: NA, p=NA |
| **Tolerability – Constipation**  **(24-48h)** | | | | | | | | |
| Caddy 2015 | | Diagnosis: Unipolar Depression  Resistant: Yes  Suicidal: No | Ketamine IV | Midazolam | NA - Single RCT | OR | Sample size: 72  Effect measure: 2.8 (0.13, 60.66)  p-value: Not reported | Het.: I²: NA, p=NA  Pub.B. Test: NA, p=NA |
| **Tolerability – Constipation**  **(7-13 days)** | | | | | | | | |
| Caddy 2015 | | Diagnosis: Unipolar Depression  Resistant: Yes  Suicidal: No | Ketamine IV | Midazolam | NA - Single RCT | OR | Sample size: 72  Effect measure: 2.23 (0.24, 21.13)  p-value: Not reported | Het.: I²: NA, p=NA  Pub.B. Test: NA, p=NA |
| **Tolerability – Constipation**  **(14-28 days)** | | | | | | | | |
| Jawad 2022 | | Diagnosis: Unipolar Depression  Resistant: No Information  Suicidal: No information | Esketamine IN | Saline | Pooled RR | RR | Sample size: NA  Effect measure: 1.488 (0.752, 2.944)  p-value: 0.254 | Het.: I²: NA, p=NA  Pub.B. Test: NA, p=NA |
| **Tolerability – Decreased energy**  **(24-48h)** | | | | | | | | |
| Caddy 2015 | | Diagnosis: Unipolar Depression  Resistant: Yes  Suicidal: No | Ketamine IV | Midazolam | NA - Single RCT | OR | Sample size: 72  Effect measure: 1.28 (0.3, 5.47)  p-value: Not reported | Het.: I²: NA, p=NA  Pub.B. Test: NA, p=NA |
| **Tolerability – Decreased energy**  **(7-13 days)** | | | | | | | | |
| Caddy 2015 | | Diagnosis: Unipolar Depression  Resistant: Yes  Suicidal: No | Ketamine IV | Midazolam | NA - Single RCT | OR | Sample size: 72  Effect measure: 0.92 (0.24, 3.5)  p-value: Not reported | Het.: I²: NA, p=NA  Pub.B. Test: NA, p=NA |
| **Tolerability – Diarrhea**  **(24-48h)** | | | | | | | | |
| Caddy 2015 | | Diagnosis: Unipolar Depression  Resistant: Yes  Suicidal: No | Ketamine IV | Midazolam | NA - Single RCT | OR | Sample size: 72  Effect measure: 1.07 (0.09, 12.37)  p-value: | Het.: I²: NA, p=NA  Pub.B. Test: NA, p=NA |
| **Tolerability – Diarrhea**  **(7-13 days)** | | | | | | | | |
| Caddy 2015 | | Diagnosis: Unipolar Depression  Resistant: Yes  Suicidal: No | Ketamine IV | Midazolam | NA - Single RCT | OR | Sample size: 72  Effect measure: 1.24 (0.34, 4.53)  p-value: | Het.: I²: NA, p=NA  Pub.B. Test: NA, p=NA |
| **Tolerability – Diarrhea**  **(14-28 days)** | | | | | | | | |
| Jawad 2022 | | Diagnosis: Unipolar Depression  Resistant: No Information  Suicidal: No information | Esketamine IN | Saline | Pooled RR | RR | Sample size: NA  Effect measure: 1.129 (0.581, 2.195)  p-value: 0.721 | Het.: I²: NA, p=NA  Pub.B. Test: NA, p=NA |
| **Tolerability – Diarrhea**  **(Endpoint)** | | | | | | | | |
| Zheng 2020 | | Diagnosis: Unipolar Depression  Resistant: No Information  Suicidal: No information | Esketamine IN | Saline | Random Effect | RR | Sample size: 680  Effect measure: 1.35 (0.72, 2.52)  p-value: 0.36 | Het.: I²: 0%, p=NA  Pub.B. Test: NA, p=NA |
| **Tolerability – Diplopia**  **(>28 days)** | | | | | | | | |
| Jawad 2022 | | Diagnosis: Unipolar Depression  Resistant: No Information  Suicidal: No information | Esketamine IN | Saline | NA - Single RCT | RR | Sample size: NA  Effect measure: 18.131 (1.065, 308.699)  p-value: 0.045 | Het.: I²: NA, p=NA  Pub.B. Test: NA, p=NA |
| **Tolerability – Discontinuation due to intolerability**  **(Endpoint)** | | | | | | | | |
| Zheng 2020 | | Diagnosis: Unipolar Depression  Resistant: No Information  Suicidal: No information | Esketamine IN | Saline | Random Effect | RR | Sample size: 798  Effect measure: 3.5 (1.38, 8.86)  p-value: 0.008 | Het.: I²: 0%, p=0.44  Pub.B. Test: NA, p=NA |
| **Tolerability – Dissociative symptoms**  **(14-28 days)** | | | | | | | | |
| Jawad 2022 | | Diagnosis: Unipolar Depression  Resistant: Not reported in some of the RCTs  Suicidal: Mixed | Esketamine IN | Saline | Pooled RR | RR | Sample size: 855  Effect measure: 5.694 (4.007, 8.093)  p-value: 0.001 | Het.: I²: NA, p=NA  Pub.B. Test: NA, p=NA |
| **Tolerability – Dissociative symptoms**  **(>28 days)** | | | | | | | | |
| Jawad 2022 | | Diagnosis: Unipolar Depression  Resistant: Yes  Suicidal: No | Esketamine IN | Saline | NA - Single RCT | RR | Sample size: NA  Effect measure: 67.752 (4.195, 1094.313)  p-value: 0.003 | Het.: I²: NA, p=NA  Pub.B. Test: NA, p=NA |
| **Tolerability – Dissociative symptoms**  **(Endpoint)** | | | | | | | | |
| Bahji 2022 | | Diagnosis: No Information  Resistant: No Information  Suicidal: No information | No Information | Saline,Midazolam | Random Effect | OR | Sample size: NA  Effect measure: 8.19 (5.62, 11.95)  p-value: Not reported | Het.: I²: 1%, p=NA  Pub.B. Test: Egger, p=NA |
| Caddy 2015 | | Diagnosis: Unipolar Depression  Resistant: Not reported in all of the RCTs  Suicidal: No | Ketamine IV | Saline | NA - Single RCT | OR | Sample size: 27  Effect measure: 16.06 (0.74, 346.19)  p-value: Not reported | Het.: I²: NA, p=NA  Pub.B. Test: NA, p=NA |
| Zheng 2020 | | Diagnosis: Unipolar Depression  Resistant: Not reported in some of the RCTs  Suicidal: Mixed | Esketamine IN | Saline | Random Effect | RR | Sample size: 267  Effect measure: 5.7 (3.53, 9.21)  p-value: 0.00001 | Het.: I²: 1%, p=NA  Pub.B. Test: NA, p=NA |
| Zheng 2020 | | Diagnosis: Unipolar Depression  Resistant: No Information  Suicidal: No information | Esketamine IN | Saline | Random Effect | RR | Sample size: 155  Effect measure: 9.25 (1.64, 52.11)  p-value: 0.01 | Het.: I²: 0%, p=NA  Pub.B. Test: NA, p=NA |
| **Tolerability - Dizziness**  **(24-48h)** | | | | | | | | |
| Caddy 2015 | | Diagnosis: Unipolar Depression  Resistant: Yes  Suicidal: No | Ketamine IV | Midazolam | NA - Single RCT | OR | Sample size: 72  Effect measure: 3.02 (1.22, 7.49)  p-value: Not reported | Het.: I²: NA, p=NA  Pub.B. Test: NA, p=NA |
| **Tolerability – Dizziness**  **(7-13 days)** | | | | | | | | |
| Caddy 2015 | | Diagnosis: Unipolar Depression  Resistant: Yes  Suicidal: No | Ketamine IV | Midazolam | NA - Single RCT | OR | Sample size: 72  Effect measure: 1.07 (0.38, 3.06)  p-value: Not reported | Het.: I²: NA, p=NA  Pub.B. Test: NA, p=NA |
| **Tolerability – Dizziness**  **(14-28 days)** | | | | | | | | |
| Jawad 2022 | | Diagnosis: Unipolar Depression  Resistant: Not reported in some of the RCTs  Suicidal: Mixed | Esketamine IN | Saline | Pooled RR | RR | Sample size: 855  Effect measure: 3.016 (2.304, 3.948)  p-value: 0.001 | Het.: I²: NA, p=NA  Pub.B. Test: NA, p=NA |
| **Tolerability – Dizziness**  **(>28 days)** | | | | | | | | |
| Jawad 2022 | | Diagnosis: Unipolar Depression  Resistant: Yes  Suicidal: No | Esketamine IN | Saline | NA - Single RCT | RR | Sample size: NA  Effect measure: 4.225 (1.921, 9.29)  p-value: 0.001 | Het.: I²: NA, p=NA  Pub.B. Test: NA, p=NA |
| **Tolerability – Dizziness**  **(Endpoint)** | | | | | | | | |
| An 2021 | | Diagnosis: Unipolar Depression  Resistant: Not reported in some of the RCTs  Suicidal: Mixed | Ketamine IN,Esketamine IN | Saline | Random Effect | RR | Sample size: 422  Effect measure: 3.3 (2.2, 4.95)  p-value: | Het.: I²: 0%, p=0.7  Pub.B. Test: NA, p=NA |
| Bahji 2022 | | Diagnosis: No Information  Resistant: No Information  Suicidal: No information | No Information | Saline,Midazolam | Random Effect | OR | Sample size: NA  Effect measure: 3.85 (2.98, 4.98)  p-value: Not reported | Het.: I²: 1%, p=NA  Pub.B. Test: Egger, p=NA |
| Zheng 2020 | | Diagnosis: Unipolar Depression  Resistant: Not reported in some of the RCTs  Suicidal: Mixed | Esketamine IN | Saline | Random Effect | RR | Sample size: 267  Effect measure: 3.49 (2.41, 5.05)  p-value: 0.00001 | Het.: I²: 0%, p=NA  Pub.B. Test: NA, p=NA |
| **Tolerability – Dry mouth**  **(24-48h)** | | | | | | | | |
| Caddy 2015 | | Diagnosis: Unipolar Depression  Resistant: Yes  Suicidal: No | Ketamine IV | Midazolam | NA - Single RCT | OR | Sample size: 72  Effect measure: 1.8 (0.51, 6.31)  p-value: Not reported | Het.: I²: NA, p=NA  Pub.B. Test: NA, p=NA |
|  | **Tolerability – Dry mouth**  **(7-13 days)** | | | | | | | |
| Caddy 2015 | | Diagnosis: Unipolar Depression  Resistant: Yes  Suicidal: No | Ketamine IV | Midazolam | NA - Single RCT | OR | Sample size: 72  Effect measure: 0.68 (0.14, 3.32)  p-value: Not reported | Het.: I²: NA, p=NA  Pub.B. Test: NA, p=NA |
| **Tolerability – Dry mouth**  **(14-28 days)** | | | | | | | | |
| Jawad 2022 | | Diagnosis: Unipolar Depression  Resistant: No Information  Suicidal: No information | Esketamine IN | Saline | Pooled RR | RR | Sample size: NA  Effect measure: 2.033 (0.888, 4.654)  p-value: 0.09 | Het.: I²: NA, p=NA  Pub.B. Test: NA, p=NA |
| **Tolerability – Dry skin**  **(24-48h)** | | | | | | | | |
| Caddy 2015 | | Diagnosis: Unipolar Depression  Resistant: Yes  Suicidal: No | Ketamine IV | Midazolam | NA - Single RCT | OR | Sample size: 72  Effect measure: 0.52 (0.03, 8.71)  p-value: Not reported | Het.: I²: NA, p=NA  Pub.B. Test: NA, p=NA |
| **Tolerability – Dry skin**  **(7-13 days)** | | | | | | | | |
| Caddy 2015 | | Diagnosis: Unipolar Depression  Resistant: Yes  Suicidal: No | Ketamine IV | Midazolam | NA - Single RCT | OR | Sample size: 72  Effect measure: 1.37 (0.42, 4.46)  p-value: Not reported | Het.: I²: NA, p=NA  Pub.B. Test: NA, p=NA |
| **Tolerability – Dysgeusia**  **(14-28 days)** | | | | | | | | |
| Jawad 2022 | | Diagnosis: Unipolar Depression  Resistant: Not reported in some of the RCTs  Suicidal: Mixed | Esketamine IN | Saline | Pooled RR | RR | Sample size: 855  Effect measure: 2.188 (1.664, 2.876)  p-value: 0.001 | Het.: I²: NA, p=NA  Pub.B. Test: NA, p=NA |
| **Tolerability - Dysgeusia**  **(>28 days)** | | | | | | | | |
| Jawad 2022 | | Diagnosis: Unipolar Depression  Resistant: Yes  Suicidal: No | Esketamine IN | Saline | NA - Single RCT | RR | Sample size: NA  Effect measure: 3.911 (2.036, 7.513)  p-value: 0.001 | Het.: I²: NA, p=NA  Pub.B. Test: NA, p=NA |
| **Tolerability – Dysgeusia**  **(Endpoint)** | | | | | | | | |
| An 2021 | | Diagnosis: Unipolar Depression  Resistant: Not reported in some of the RCTs  Suicidal: Mixed | Ketamine IN,Esketamine IN | Saline | Random Effect | RR | Sample size: 422  Effect measure: 1.37 (0.99, 1.89)  p-value: | Het.: I²: 0%, p=0.48  Pub.B. Test: NA, p=NA |
| Bahji 2022 | | Diagnosis: No Information  Resistant: No Information  Suicidal: No information | No Information | Saline,Midazolam | Random Effect | OR | Sample size: NA  Effect measure: 1.88 (1.28, 2.76)  p-value: Not reported | Het.: I²: 39%, p=NA  Pub.B. Test: Egger, p=NA |
| Zheng 2020 | | Diagnosis: Unipolar Depression  Resistant: Not reported in some of the RCTs  Suicidal: Mixed | Esketamine IN | Saline | Random Effect | RR | Sample size: 267  Effect measure: 1.28 (0.94, 1.75)  p-value: 0.12 | Het.: I²: 8%, p=NA  Pub.B. Test: NA, p=NA |
| **Tolerability – Emotional blunting**  **(Endpoint)** | | | | | | | | |
| Caddy 2015 | | Diagnosis: Unipolar Depression  Resistant: Not reported in all of the RCTs  Suicidal: No | Ketamine (Any Route) | Placebo/Sham | NA - Single RCT | OR | Sample size: 27  Effect measure: 23.4 (1.12, 489.52)  p-value: 0.04 | Het.: I²: NA, p=NA  Pub.B. Test: NA, p=NA |
| Caddy 2015 | | Diagnosis: Unipolar Depression  Resistant: Not reported in all of the RCTs  Suicidal: No | Ketamine IV | Saline | NA - Single RCT | OR | Sample size: 27  Effect measure: 23.4 (1.12, 489.52)  p-value: 0.04 | Het.: I²: NA, p=NA  Pub.B. Test: NA, p=NA |
| **Tolerability – Euphoria**  **(14-28 days)** | | | | | | | | |
| Jawad 2022 | | Diagnosis: Unipolar Depression  Resistant: No Information  Suicidal: No information | Esketamine IN | Saline | Pooled RR | RR | Sample size: NA  Effect measure: 3.408 (0.942, 12.332)  p-value: 0.062 | Het.: I²: NA, p=NA  Pub.B. Test: NA, p=NA |
| **Tolerability – Euphoria**  **(Endpoint)** | | | | | | | | |
| Caddy 2015 | | Diagnosis: Unipolar Depression  Resistant: Not reported in all of the RCTs  Suicidal: No | Ketamine IV | Saline | NA - Single RCT | OR | Sample size: 27  Effect measure: 10.26 (0.45, 235.66)  p-value: Not reported | Het.: I²: NA, p=NA  Pub.B. Test: NA, p=NA |
| Zheng 2020 | | Diagnosis: Unipolar Depression  Resistant: No Information  Suicidal: No information | Esketamine IN | Saline | Random Effect | RR | Sample size: 523  Effect measure: 2.1 (0.8, 5.52)  p-value: 0.13 | Het.: I²: 0%, p=NA  Pub.B. Test: NA, p=NA |
| **Tolerability – Fatigue**  **(24-48h)** | | | | | | | | |
| Caddy 2015 | | Diagnosis: Unipolar Depression  Resistant: Yes  Suicidal: No | Ketamine IV | Midazolam | NA - Single RCT | OR | Sample size: 72  Effect measure: 2.01 (0.39, 10.51)  p-value: Not reported | Het.: I²: NA, p=NA  Pub.B. Test: NA, p=NA |
| **Tolerability – Fatigue**  **(7-13 days)** | | | | | | | | |
| Caddy 2015 | | Diagnosis: Unipolar Depression  Resistant: Yes  Suicidal: No | Ketamine IV | Midazolam | NA - Single RCT | OR | Sample size: 72  Effect measure: 1.42 (0.4, 5.09)  p-value: Not reported | Het.: I²: NA, p=NA  Pub.B. Test: NA, p=NA |
| **Tolerability – Fatigue**  **(14-28 days)** | | | | | | | | |
| Jawad 2022 | | Diagnosis: Unipolar Depression  Resistant: No Information  Suicidal: No information | Esketamine IN | Saline | Pooled RR | RR | Sample size: NA  Effect measure: 1.382 (0.73, 2.618)  p-value: 0.321 | Het.: I²: NA, p=NA  Pub.B. Test: NA, p=NA |
| **Tolerability – Fatigue**  **(Endpoint)** | | | | | | | | |
| Zheng 2020 | | Diagnosis: Unipolar Depression  Resistant: No Information  Suicidal: No information | Esketamine IN | Saline | Random Effect | RR | Sample size: 680  Effect measure: 1.5 (0.81, 2.8)  p-value: 0.2 | Het.: I²: 0%, p=NA  Pub.B. Test: NA, p=NA |
| **Tolerability – Feeling abnormal**  **(Endpoint)** | | | | | | | | |
| Zheng 2020 | | Diagnosis: Unipolar Depression  Resistant: No Information  Suicidal: No information | Esketamine IN | Saline | Random Effect | RR | Sample size: 155  Effect measure: 6.24 (1.05, 37.19)  p-value: 0.04 | Het.: I²: 0%, p=NA  Pub.B. Test: NA, p=NA |
| **Tolerability – Feeling drunk**  **(14-28 days)** | | | | | | | | |
| Jawad 2022 | | Diagnosis: Unipolar Depression  Resistant: No Information  Suicidal: No information | Esketamine IN | Saline | Pooled RR | RR | Sample size: NA  Effect measure: 5.351 (1.336, 21.433)  p-value: 0.018 | Het.: I²: NA, p=NA  Pub.B. Test: NA, p=NA |
| **Tolerability – Feeling drunk**  **(Endpoint)** | | | | | | | | |
| Zheng 2020 | | Diagnosis: Unipolar Depression  Resistant: No Information  Suicidal: No information | Esketamine IN | Saline | Random Effect | RR | Sample size: 680  Effect measure: 9.35 (2.19, 39.85)  p-value: 0.003 | Het.: I²: 0%, p=NA  Pub.B. Test: NA, p=NA |
| **Tolerability – General malaise**  **(24-48h)** | | | | | | | | |
| Caddy 2015 | | Diagnosis: Unipolar Depression  Resistant: Yes  Suicidal: No | Ketamine IV | Midazolam | NA - Single RCT | OR | Sample size: 72  Effect measure: 0.18 (0.04, 0.75)  p-value: 0.02 | Het.: I²: NA, p=NA  Pub.B. Test: NA, p=NA |
| **Tolerability – General malaise**  **(7-13 days)** | | | | | | | | |
| Caddy 2015 | | Diagnosis: Unipolar Depression  Resistant: Yes  Suicidal: No | Ketamine IV | Midazolam | NA - Single RCT | OR | Sample size: 72  Effect measure: 0.69 (0.23, 2.13)  p-value: 0.52 | Het.: I²: NA, p=NA  Pub.B. Test: NA, p=NA |
| **Tolerability – Headache**  **(24-48h)** | | | | | | | | |
| Caddy 2015 | | Diagnosis: Unipolar Depression  Resistant: Yes  Suicidal: No | Ketamine IV | Midazolam | NA - Single RCT | OR | Sample size: 72  Effect measure: 1.88 (0.59, 5.96)  p-value: Not reported | Het.: I²: NA, p=NA  Pub.B. Test: NA, p=NA |
| **Tolerability – Headache**  **(7-13 days)** | | | | | | | | |
| Caddy 2015 | | Diagnosis: Unipolar Depression  Resistant: Yes  Suicidal: No | Ketamine IV | Midazolam | NA - Single RCT | OR | Sample size: 72  Effect measure: 1.21 (0.41, 3.5)  p-value: Not reported | Het.: I²: NA, p=NA  Pub.B. Test: NA, p=NA |
| **Tolerability – Headache**  **(14-28 days)** | | | | | | | | |
| Jawad 2022 | | Diagnosis: Unipolar Depression  Resistant: Not reported in some of the RCTs  Suicidal: Mixed | Esketamine IN | Saline | Pooled RR | RR | Sample size: 855  Effect measure: 1.163 (0.911, 1.483)  p-value: 0.225 | Het.: I²: NA, p=NA  Pub.B. Test: NA, p=NA |
| **Tolerability – Headache**  **(>28 days)** | | | | | | | | |
| Jawad 2022 | | Diagnosis: Unipolar Depression  Resistant: Yes  Suicidal: No | Esketamine IN | Saline | NA - Single RCT | RR | Sample size: NA  Effect measure: 1.84 (1.006, 3.366)  p-value: 0.048 | Het.: I²: NA, p=NA  Pub.B. Test: NA, p=NA |
| **Tolerability – Headache**  **(Endpoint)** | | | | | | | | |
| Bahji 2022 | | Diagnosis: No Information  Resistant: No Information  Suicidal: No information | No Information | Saline,Midazolam | Random Effect | OR | Sample size: NA  Effect measure: 1.38 (1.05, 1.82)  p-value: Not reported | Het.: I²: 16%, p=NA  Pub.B. Test: Egger, p=NA |
| Zheng 2020 | | Diagnosis: Unipolar Depression  Resistant: Not reported in some of the RCTs  Suicidal: Mixed | Esketamine IN | Saline | Random Effect | RR | Sample size: 267  Effect measure: 1.29 (0.98, 1.7)  p-value: 0.07 | Het.: I²: 0%, p=NA  Pub.B. Test: NA, p=NA |
| **Tolerability – Hypoestesia**  **(14-28 days)** | | | | | | | | |
| Jawad 2022 | | Diagnosis: Unipolar Depression  Resistant: No Information  Suicidal: No information | Esketamine IN | Saline | Pooled RR | RR | Sample size: NA  Effect measure: 4.818 (2.466, 9.412)  p-value: 0.001 | Het.: I²: NA, p=NA  Pub.B. Test: NA, p=NA |
| **Tolerability – Hypoestesia**  **(>28 days)** | | | | | | | | |
| Jawad 2022 | | Diagnosis: Unipolar Depression  Resistant: Yes  Suicidal: No | Esketamine IN | Saline | NA - Single RCT | RR | Sample size: NA  Effect measure: 18.131 (1.065, 308.699)  p-value: 0.045 | Het.: I²: NA, p=NA  Pub.B. Test: NA, p=NA |
| **Tolerability – Hypoestesia**  **(Endpoint)** | | | | | | | | |
| Bahji 2022 | | Diagnosis: No Information  Resistant: No Information  Suicidal: No information | No Information | Saline,Midazolam | Random Effect | OR | Sample size: NA  Effect measure: 8.57 (4.23, 17.37)  p-value: Not reported | Het.: I²: 1%, p=NA  Pub.B. Test: Egger, p=NA |
| Zheng 2020 | | Diagnosis: Unipolar Depression  Resistant: No Information  Suicidal: No information | Esketamine IN | Saline | Random Effect | RR | Sample size: 680  Effect measure: 7.39 (2.95, 18.53)  p-value: 0.0001 | Het.: I²: 0%, p=NA  Pub.B. Test: NA, p=NA |
| **Tolerability – Hypoesthesia oral**  **(14-28 days)** | | | | | | | | |
| Jawad 2022 | | Diagnosis: Unipolar Depression  Resistant: No Information  Suicidal: No information | Esketamine IN | Saline | Pooled RR | RR | Sample size: NA  Effect measure: 5.832 (2.463, 13.81)  p-value: 0.001 | Het.: I²: NA, p=NA  Pub.B. Test: NA, p=NA |
| **Tolerability – Hypoesthesia oral**  **(>28 days)** | | | | | | | | |
| Jawad 2022 | | Diagnosis: Unipolar Depression  Resistant: Yes  Suicidal: No | Esketamine IN | Saline | NA - Single RCT | RR | Sample size: NA  Effect measure: 39.124 (2.388, 640.961)  p-value: 0.01 | Het.: I²: NA, p=NA  Pub.B. Test: NA, p=NA |
| **Tolerability – Hypoesthesia oral**  **(Endpoint)** | | | | | | | | |
| Zheng 2020 | | Diagnosis: Unipolar Depression  Resistant: No Information  Suicidal: No information | Esketamine IN | Saline | Random Effect | RR | Sample size: 835  Effect measure: 7.54 (3.34, 17.01)  p-value: 0.00001 | Het.: I²: 0%, p=NA  Pub.B. Test: NA, p=NA |
| **Tolerability – Increased blood pressure**  **(14-28 days)** | | | | | | | | |
| Jawad 2022 | | Diagnosis: Unipolar Depression  Resistant: No Information  Suicidal: No information | Esketamine IN | Saline | Pooled RR | RR | Sample size: NA  Effect measure: 3.401 (2.215, 5.224)  p-value: 0.001 | Het.: I²: NA, p=NA  Pub.B. Test: NA, p=NA |
| **Tolerability – Increased blood pressure**  **(>28 days)** | | | | | | | | |
| Jawad 2022 | | Diagnosis: Unipolar Depression  Resistant: Yes  Suicidal: No | Esketamine IN | Saline | NA - Single RCT | RR | Sample size: NA  Effect measure: 1.908 (0.668, 5.447)  p-value: 0.227 | Het.: I²: NA, p=NA  Pub.B. Test: NA, p=NA |
| **Tolerability – Increased blood pressure**  **(Endpoint)** | | | | | | | | |
| Bahji 2022 | | Diagnosis: No Information  Resistant: No Information  Suicidal: No information | No Information | Saline,Midazolam | Random Effect | OR | Sample size: NA  Effect measure: 2.53 (1.56, 4.11)  p-value: Not reported | Het.: I²: 1%, p=NA  Pub.B. Test: Egger, p=NA |
| Zheng 2020 | | Diagnosis: Unipolar Depression  Resistant: No Information  Suicidal: No information | Esketamine IN | Saline | Random Effect | RR | Sample size: 155  Effect measure: 1.03 (0.28, 3.82)  p-value: 0.97 | Het.: I²: 0%, p=NA  Pub.B. Test: NA, p=NA |
| **Tolerability – Increased perspiration**  **(24-48h)** | | | | | | | | |
| Caddy 2015 | | Diagnosis: Unipolar Depression  Resistant: Yes  Suicidal: No | Ketamine IV | Midazolam | NA - Single RCT | OR | Sample size: 72  Effect measure: 2.86 (0.32, 25.91)  p-value: Not reported | Het.: I²: NA, p=NA  Pub.B. Test: NA, p=NA |
| **Tolerability – Increased perspiration**  **(7-13 days)** | | | | | | | | |
| Caddy 2015 | | Diagnosis: Unipolar Depression  Resistant: Yes  Suicidal: No | Ketamine IV | Midazolam | NA - Single RCT | OR | Sample size: 72  Effect measure: 2.86 (0.32, 25.91)  p-value: Not reported | Het.: I²: NA, p=NA  Pub.B. Test: NA, p=NA |
| **Tolerability – Insomnia**  **(14-28 days)** | | | | | | | | |
| Jawad 2022 | | Diagnosis: Unipolar Depression  Resistant: No Information  Suicidal: No information | Esketamine IN | Saline | Pooled RR | RR | Sample size: NA  Effect measure: 1.008 (0.648, 1.568)  p-value: 0.973 | Het.: I²: NA, p=NA  Pub.B. Test: NA, p=NA |
| **Tolerability – Insomnia**  **(Endpoint)** | | | | | | | | |
| Zheng 2020 | | Diagnosis: Unipolar Depression  Resistant: No Information  Suicidal: No information | Esketamine IN | Saline | Random Effect | RR | Sample size: 835  Effect measure: 1.05 (0.64, 1.72)  p-value: 0.85 | Het.: I²: 0%, p=NA  Pub.B. Test: NA, p=NA |
| **Tolerability - Itching**  **(24-48h)** | | | | | | | | |
| Caddy 2015 | | Diagnosis: Unipolar Depression  Resistant: Yes  Suicidal: No | Ketamine IV | Midazolam | NA - Single RCT | OR | Sample size: 72  Effect measure: 1.07 (0.09, 12.37)  p-value: Not reported | Het.: I²: NA, p=NA  Pub.B. Test: NA, p=NA |
| **Tolerability - Itching**  **(7-13 days)** | | | | | | | | |
| Caddy 2015 | | Diagnosis: Unipolar Depression  Resistant: Yes  Suicidal: No | Ketamine IV | Midazolam | NA - Single RCT | OR | Sample size: 72  Effect measure: 3.51 (0.4, 30.95)  p-value: Not reported | Het.: I²: NA, p=NA  Pub.B. Test: NA, p=NA |
| **Tolerability - Lethargy**  **(Endpoint)** | | | | | | | | |
| Zheng 2020 | | Diagnosis: Unipolar Depression  Resistant: No Information  Suicidal: No information | Esketamine IN | Saline | Random Effect | RR | Sample size: 457  Effect measure: 5.81 (1.31, 25.75)  p-value: 0.02 | Het.: I²: 0%, p=NA  Pub.B. Test: NA, p=NA |
| **Tolerability – Mental impairment**  **(Endpoint)** | | | | | | | | |
| Zheng 2020 | | Diagnosis: Unipolar Depression  Resistant: No Information  Suicidal: No information | Esketamine IN | Saline | Random Effect | RR | Sample size: 457  Effect measure: 4.25 (0.92, 19.73)  p-value: 0.06 | Het.: I²: 0%, p=NA  Pub.B. Test: NA, p=NA |
| **Tolerability – Nasal discomfort**  **(14-28 days)** | | | | | | | | |
| Jawad 2022 | | Diagnosis: Unipolar Depression  Resistant: No Information  Suicidal: No information | Esketamine IN | Saline | Pooled RR | RR | Sample size: NA  Effect measure: 1.743 (0.614, 4.944)  p-value: 0.297 | Het.: I²: NA, p=NA  Pub.B. Test: NA, p=NA |
| **Tolerability – Nasal discomfort**  **(>28 days)** | | | | | | | | |
| Jawad 2022 | | Diagnosis: Unipolar Depression  Resistant: Yes  Suicidal: No | Esketamine IN | Saline | NA - Single RCT | RR | Sample size: NA  Effect measure: 2.623 (0.855, 8.052)  p-value: 0.092 | Het.: I²: NA, p=NA  Pub.B. Test: NA, p=NA |
| **Tolerability – Nasal discomfort**  **(Endpoint)** | | | | | | | | |
| Zheng 2020 | | Diagnosis: Unipolar Depression  Resistant: No Information  Suicidal: No information | Esketamine IN | Saline | Random Effect | RR | Sample size: 835  Effect measure: 0.88 (0.47, 1.66)  p-value: 0.69 | Het.: I²: 3%, p=NA  Pub.B. Test: NA, p=NA |
| **Tolerability - Nausea**  **(14-28 days)** | | | | | | | | |
| Jawad 2022 | | Diagnosis: Unipolar Depression  Resistant: Not reported in some of the RCTs  Suicidal: Mixed | Esketamine IN | Saline | Pooled RR | RR | Sample size: 855  Effect measure: 2.559 (1.939, 3.377)  p-value: 0.001 | Het.: I²: NA, p=NA  Pub.B. Test: NA, p=NA |
| **Tolerability - Nausea**  **(>28 days)** | | | | | | | | |
| Jawad 2022 | | Diagnosis: Unipolar Depression  Resistant: Yes  Suicidal: No | Esketamine IN | Saline | NA - Single RCT | RR | Sample size: NA  Effect measure: 23.849 (3.274, 173.732)  p-value: 0.002 | Het.: I²: NA, p=NA  Pub.B. Test: NA, p=NA |
| **Tolerability - Nausea**  **(Endpoint)** | | | | | | | | |
| An 2021 | | Diagnosis: Unipolar Depression  Resistant: Not reported in some of the RCTs  Suicidal: Mixed | Ketamine IN,Esketamine IN | Saline | Random Effect | RR | Sample size: 422  Effect measure: 3.25 (2.19, 4.84)  p-value: | Het.: I²: 0%, p=0.56  Pub.B. Test: NA, p=NA |
| Bahji 2022 | | Diagnosis: No Information  Resistant: No Information  Suicidal: No information | No Information | Saline,Midazolam | Random Effect | OR | Sample size: NA  Effect measure: 3.09 (2.23, 4.27)  p-value: Not reported | Het.: I²: 15%, p=NA  Pub.B. Test: NA, p=NA |
| Zheng 2020 | | Diagnosis: Unipolar Depression  Resistant: Not reported in some of the RCTs  Suicidal: Mixed | Esketamine IN | Saline | Random Effect | RR | Sample size: 267  Effect measure: 2.95 (2.1, 4.13)  p-value: 0.00001 | Het.: I²: 0%, p=NA  Pub.B. Test: NA, p=NA |
| **Tolerability – Nausea/Vomiting**  **(24-48h)** | | | | | | | | |
| Caddy 2015 | | Diagnosis: Unipolar Depression  Resistant: Yes  Suicidal: No | Ketamine IV | Midazolam | NA - Single RCT | OR | Sample size: 72  Effect measure: 3.78 (0.98, 14.58)  p-value: 0.05 | Het.: I²: NA, p=NA  Pub.B. Test: NA, p=NA |
| **Tolerability – Nausea/Vomiting**  **(7-13 days)** | | | | | | | | |
| Caddy 2015 | | Diagnosis: Unipolar Depression  Resistant: Yes  Suicidal: No | Ketamine IV | Midazolam | NA - Single RCT | OR | Sample size: 72  Effect measure: 2.01 (0.39, 10.51)  p-value: 0.41 | Het.: I²: NA, p=NA  Pub.B. Test: NA, p=NA |
| **Tolerability – Neurological symptoms**  **(Endpoint)** | | | | | | | | |
| Nuñez 2020 | | Diagnosis: Unipolar Depression  Resistant: Not reported in some of the RCTs  Suicidal: Not reported in some of the RCTs | Ketamine Oral | Diclofenac,Saline | Random Effect | RR | Sample size: 161  Effect measure: 1.51 (0.77, 2.94)  p-value: 0.228 | Het.: I²: 0%, p=0.561  Pub.B. Test: NA, p=NA |
| **Tolerability – Oral paresthesia**  **(14-28 days)** | | | | | | | | |
| Jawad 2022 | | Diagnosis: Unipolar Depression  Resistant: No Information  Suicidal: No information | Esketamine IN | Saline | Pooled RR | RR | Sample size: NA  Effect measure: 3.683 (1.411, 9.615)  p-value: 0.008 | Het.: I²: NA, p=NA  Pub.B. Test: NA, p=NA |
| **Tolerability – Oral paresthesia**  **(>28 days)** | | | | | | | | |
| Jawad 2022 | | Diagnosis: Unipolar Depression  Resistant: Yes  Suicidal: No | Esketamine IN | Saline | NA - Single RCT | RR | Sample size: NA  Effect measure: 7.632 (0.967, 60.26)  p-value: 0.054 | Het.: I²: NA, p=NA  Pub.B. Test: NA, p=NA |
| **Tolerability – Oral paresthesia**  **(Endpoint)** | | | | | | | | |
| Zheng 2020 | | Diagnosis: Unipolar Depression  Resistant: No Information  Suicidal: No information | Esketamine IN | Saline | Random Effect | RR | Sample size: 680  Effect measure: 3.08 (0.7, 13.56)  p-value: 0.14 | Het.: I²: 42%, p=NA  Pub.B. Test: NA, p=NA |
| **Tolerability – Oropharyngeal pain**  **(14-28 days)** | | | | | | | | |
| Jawad 2022 | | Diagnosis: Unipolar Depression  Resistant: No Information  Suicidal: No information | Esketamine IN | Saline | Pooled RR | RR | Sample size: NA  Effect measure: 1.988 (0.655, 6.093)  p-value: 0.225 | Het.: I²: NA, p=NA  Pub.B. Test: NA, p=NA |
| **Tolerability – Oropharyngeal pain**  **(Endpoint)** | | | | | | | | |
| Zheng 2020 | | Diagnosis: Unipolar Depression  Resistant: No Information  Suicidal: No information | Esketamine IN | Saline | Random Effect | RR | Sample size: 155  Effect measure: 1.06 (0.29, 3.93)  p-value: 0.93 | Het.: I²: 0%, p=NA  Pub.B. Test: NA, p=NA |
| **Tolerability - Palpitations**  **(24-48h)** | | | | | | | | |
| Caddy 2015 | | Diagnosis: Unipolar Depression  Resistant: Yes  Suicidal: No | Ketamine IV | Midazolam | NA - Single RCT | OR | Sample size: 72  Effect measure: 6.6 (0.35, 124.39)  p-value: Not reported | Het.: I²: NA, p=NA  Pub.B. Test: NA, p=NA |
| **Tolerability - Palpitations**  **(7-13 days)** | | | | | | | | |
| Caddy 2015 | | Diagnosis: Unipolar Depression  Resistant: Yes  Suicidal: No | Ketamine IV | Midazolam | NA - Single RCT | OR | Sample size: 72  Effect measure: 1.68 (0.31, 9.03)  p-value: Not reported | Het.: I²: NA, p=NA  Pub.B. Test: NA, p=NA |
| **Tolerability - Paresthesia**  **(14-28 days)** | | | | | | | | |
| Jawad 2022 | | Diagnosis: Unipolar Depression  Resistant: No Information  Suicidal: No information | Esketamine IN | Saline | Pooled RR | RR | Sample size: NA  Effect measure: 3.585 (2.018, 6.371)  p-value: 0.001 | Het.: I²: NA, p=NA  Pub.B. Test: NA, p=NA |
| **Tolerability - Paresthesia**  **(>28 days)** | | | | | | | | |
| Jawad 2022 | | Diagnosis: Unipolar Depression  Resistant: Yes  Suicidal: No | Esketamine IN | Saline | NA - Single RCT | RR | Sample size: NA  Effect measure: 21.948 (1.305, 369.072)  p-value: 0.032 | Het.: I²: NA, p=NA  Pub.B. Test: NA, p=NA |
| **Tolerability - Paresthesia**  **(Endpoint)** | | | | | | | | |
| Bahji 2022 | | Diagnosis: No Information  Resistant: No Information  Suicidal: No information | No Information | Saline,Midazolam | Random Effect | OR | Sample size: NA  Effect measure: 4.8 (2.89, 7.96)  p-value: Not reported | Het.: I²: 1%, p=NA  Pub.B. Test: Egger, p=NA |
| Zheng 2020 | | Diagnosis: Unipolar Depression  Resistant: No Information  Suicidal: No information | Esketamine IN | Saline | Random Effect | RR | Sample size: 746  Effect measure: 5.51 (2.63, 11.55)  p-value: 0.00001 | Het.: I²: 0%, p=NA  Pub.B. Test: NA, p=NA |
| **Tolerability - Pollakiuria**  **(Endpoint)** | | | | | | | | |
| Zheng 2020 | | Diagnosis: Unipolar Depression  Resistant: No Information  Suicidal: No information | Esketamine IN | Saline | Random Effect | RR | Sample size: 457  Effect measure: 3.64 (0.75, 17.6)  p-value: 0.11 | Het.: I²: 0%, p=NA  Pub.B. Test: NA, p=NA |
| **Tolerability – Poor co-ordination**  **(7-13 days)** | | | | | | | | |
| Caddy 2015 | | Diagnosis: Unipolar Depression  Resistant: Yes  Suicidal: No | Ketamine IV | Midazolam | NA - Single RCT | OR | Sample size: 72  Effect measure: 1.07 (0.09, 12.37)  p-value: Not reported | Het.: I²: NA, p=NA  Pub.B. Test: NA, p=NA |
| **Tolerability – Poor concentration**  **(24-48h)** | | | | | | | | |
| Caddy 2015 | | Diagnosis: Unipolar Depression  Resistant: Yes  Suicidal: No | Ketamine IV | Midazolam | NA - Single RCT | OR | Sample size: 72  Effect measure: 3.94 (0.81, 19.27)  p-value: Not reported | Het.: I²: NA, p=NA  Pub.B. Test: NA, p=NA |
| **Tolerability – Poor concentration**  **(7-13 days)** | | | | | | | | |
| Caddy 2015 | | Diagnosis: Unipolar Depression  Resistant: Yes  Suicidal: No | Ketamine IV | Midazolam | NA - Single RCT | OR | Sample size: 72  Effect measure: 2.51 (0.64, 9.92)  p-value: Not reported | Het.: I²: NA, p=NA  Pub.B. Test: NA, p=NA |
| **Tolerability – Postural dizziness**  **(14-28 days)** | | | | | | | | |
| Jawad 2022 | | Diagnosis: Unipolar Depression  Resistant: No Information  Suicidal: No information | Esketamine IN | Saline | Pooled RR | RR | Sample size: NA  Effect measure: 6.189 (2.393, 16.006)  p-value: 0.001 | Het.: I²: NA, p=NA  Pub.B. Test: NA, p=NA |
| **Tolerability – Postural dizziness**  **(>28 days)** | | | | | | | | |
| Jawad 2022 | | Diagnosis: Unipolar Depression  Resistant: Yes  Suicidal: No | Esketamine IN | Saline | NA - Single RCT | RR | Sample size: NA  Effect measure: 3.18 (0.892, 11.323)  p-value: 0.074 | Het.: I²: NA, p=NA  Pub.B. Test: NA, p=NA |
| **Tolerability – Postural dizziness**  **(Endpoint)** | | | | | | | | |
| Zheng 2020 | | Diagnosis: Unipolar Depression  Resistant: No Information  Suicidal: No information | Esketamine IN | Saline | Random Effect | RR | Sample size: 680  Effect measure: 10.67 (2.53, 45.13)  p-value: 0.001 | Het.: I²: 0%, p=NA  Pub.B. Test: NA, p=NA |
| **Tolerability – Psychiatric symptoms**  **(Endpoint)** | | | | | | | | |
| Nuñez 2020 | | Diagnosis: Unipolar Depression  Resistant: Not reported in some of the RCTs  Suicidal: Not reported in some of the RCTs | Ketamine Oral | Diclofenac,Saline | Random Effect | RR | Sample size: 161  Effect measure: 1.44 (0.42, 4.95)  p-value: 0.56 | Het.: I²: 17.49%, p=0.298  Pub.B. Test: NA, p=NA |
| **Tolerability – Rash**  **(24-48h)** | | | | | | | | |
| Caddy 2015 | | Diagnosis: Unipolar Depression  Resistant: Yes  Suicidal: No | Ketamine IV | Midazolam | NA - Single RCT | OR | Sample size: 72  Effect measure: 1.65 (0.06, 41.87)  p-value: Not reported | Het.: I²: NA, p=NA  Pub.B. Test: NA, p=NA |
| **Tolerability – Rash**  **(7-13 days)** | | | | | | | | |
| Caddy 2015 | | Diagnosis: Unipolar Depression  Resistant: Yes  Suicidal: No | Ketamine IV | Midazolam | NA - Single RCT | OR | Sample size: 72  Effect measure: 2.86 (0.32, 25.91)  p-value: Not reported | Het.: I²: NA, p=NA  Pub.B. Test: NA, p=NA |
| **Tolerability – Sedation**  **(14-28 days)** | | | | | | | | |
| Jawad 2022 | | Diagnosis: Unipolar Depression  Resistant: No Information  Suicidal: No information | Esketamine IN | Saline | Pooled RR | RR | Sample size: NA  Effect measure: 4.735 (2.288, 9.799)  p-value: 0.001 | Het.: I²: NA, p=NA  Pub.B. Test: NA, p=NA |
| **Tolerability - Sedation**  **(>28 days)** | | | | | | | | |
| Jawad 2022 | | Diagnosis: Unipolar Depression  Resistant: Yes  Suicidal: No | Esketamine IN | Saline | NA - Single RCT | RR | Sample size: NA  Effect measure: 9.539 (1.237, 73.585)  p-value: 0.03 | Het.: I²: NA, p=NA  Pub.B. Test: NA, p=NA |
| **Tolerability – Sedation**  **(Endpoint)** | | | | | | | | |
| Zheng 2020 | | Diagnosis: Unipolar Depression  Resistant: No Information  Suicidal: No information | Esketamine IN | Saline | Random Effect | RR | Sample size: 678  Effect measure: 4.75 (1.91, 11.82)  p-value: 0.0008 | Het.: I²: 0%, p=NA  Pub.B. Test: NA, p=NA |
| **Tolerability – Somnolence**  **(14-28 days)** | | | | | | | | |
| Jawad 2022 | | Diagnosis: Unipolar Depression  Resistant: No Information  Suicidal: No information | Esketamine IN | Saline | Pooled RR | RR | Sample size: NA  Effect measure: 1.818 (1.343, 2.461)  p-value: 0.001 | Het.: I²: NA, p=NA  Pub.B. Test: NA, p=NA |
| **Tolerability – Somnolence**  **(>28 days)** | | | | | | | | |
| Jawad 2022 | | Diagnosis: Unipolar Depression  Resistant: Yes  Suicidal: No | Esketamine IN | Saline | NA - Single RCT | RR | Sample size: NA  Effect measure: 10.175 (3.186, 35.503)  p-value: 0.001 | Het.: I²: NA, p=NA  Pub.B. Test: NA, p=NA |
| **Tolerability – Somnolence**  **(Endpoint)** | | | | | | | | |
| Bahji 2022 | | Diagnosis: No Information  Resistant: No Information  Suicidal: No information | No Information | Saline,Midazolam | Random Effect | OR | Sample size: NA  Effect measure: 3.06 (1.9, 4.95)  p-value: Not reported | Het.: I²: 34%, p=NA  Pub.B. Test: Egger, p=NA |
| Zheng 2020 | | Diagnosis: Unipolar Depression  Resistant: No Information  Suicidal: No information | Esketamine IN | Saline | Random Effect | RR | Sample size: 746  Effect measure: 1.76 (1.2, 2.59)  p-value: 0.004 | Het.: I²: 0%, p=NA  Pub.B. Test: NA, p=NA |
| **Tolerability – Suicidal ideation**  **(14-28 days)** | | | | | | | | |
| Jawad 2022 | | Diagnosis: Unipolar Depression  Resistant: No Information  Suicidal: No information | Esketamine IN | Saline | Pooled RR | RR | Sample size: NA  Effect measure: 1.045 (0.388, 2.818)  p-value: 0.93 | Het.: I²: NA, p=NA  Pub.B. Test: NA, p=NA |
| **Tolerability – Throat irritation**  **(14-28 days)** | | | | | | | | |
| Jawad 2022 | | Diagnosis: Unipolar Depression  Resistant: No Information  Suicidal: No information | Esketamine IN | Saline | Pooled RR | RR | Sample size: NA  Effect measure: 1.933 (1.105, 3.683)  p-value: 0.045 | Het.: I²: NA, p=NA  Pub.B. Test: NA, p=NA |
| **Tolerability – Throat irritation**  **(>28 days)** | | | | | | | | |
| Jawad 2022 | | Diagnosis: Unipolar Depression  Resistant: Yes  Suicidal: No | Esketamine IN | Saline | NA - Single RCT | RR | Sample size: NA  Effect measure: 7.632 (0.967, 60.26)  p-value: 0.054 | Het.: I²: NA, p=NA  Pub.B. Test: NA, p=NA |
| **Tolerability – Throat irritation**  **(Endpoint)** | | | | | | | | |
| Zheng 2020 | | Diagnosis: Unipolar Depression  Resistant: No Information  Suicidal: No information | Esketamine IN | Saline | Random Effect | RR | Sample size: 782  Effect measure: 1.93 (1.02, 3.65)  p-value: 0.04 | Het.: I²: 0%, p=NA  Pub.B. Test: NA, p=NA |
| **Tolerability – Tinnitus**  **(24-48h)** | | | | | | | | |
| Caddy 2015 | | Diagnosis: Unipolar Depression  Resistant: Yes  Suicidal: No | Ketamine IV | Midazolam | NA - Single RCT | OR | Sample size: 72  Effect measure: 1.07 (0.09, 12.37)  p-value: Not reported | Het.: I²: NA, p=NA  Pub.B. Test: NA, p=NA |
| **Tolerability – Tinnitus**  **(7-13 days)** | | | | | | | | |
| Caddy 2015 | | Diagnosis: Unipolar Depression  Resistant: Yes  Suicidal: No | Ketamine IV | Midazolam | NA - Single RCT | OR | Sample size: 72  Effect measure: 0.52 (0.03, 8.71)  p-value: | Het.: I²: NA, p=NA  Pub.B. Test: NA, p=NA |
| **Tolerability – Tremor**  **(24-48h)** | | | | | | | | |
| Caddy 2015 | | Diagnosis: Unipolar Depression  Resistant: Yes  Suicidal: No | Ketamine IV | Midazolam | NA - Single RCT | OR | Sample size: 72  Effect measure: 7.99 (0.43, 147.87)  p-value: | Het.: I²: NA, p=NA  Pub.B. Test: NA, p=NA |
| **Tolerability – Tremor**  **(7-13 days)** | | | | | | | | |
| Caddy 2015 | | Diagnosis: Unipolar Depression  Resistant: Yes  Suicidal: No | Ketamine IV | Midazolam | NA - Single RCT | OR | Sample size: 72  Effect measure: 0.78 (0.12, 5.03)  p-value: | Het.: I²: NA, p=NA  Pub.B. Test: NA, p=NA |
| **Tolerability – Tremor**  **(Endpoint)** | | | | | | | | |
| Zheng 2020 | | Diagnosis: Unipolar Depression  Resistant: No Information  Suicidal: No information | Esketamine IN | Saline | Random Effect | RR | Sample size: 457  Effect measure: 2.43 (0.77, 7.66)  p-value: 0.13 | Het.: I²: 0%, p=NA  Pub.B. Test: NA, p=NA |
| **Tolerability – Urination issues**  **(24-48h)** | | | | | | | | |
| Caddy 2015 | | Diagnosis: Unipolar Depression  Resistant: Yes  Suicidal: No | Ketamine IV | Midazolam | NA - Single RCT | OR | Sample size: 72  Effect measure: 88888 (88888, 88888)  p-value: Not reported | Het.: I²: NA, p=NA  Pub.B. Test: NA, p=NA |
| **Tolerability – Urination issues**  **(7-13 days)** | | | | | | | | |
| Caddy 2015 | | Diagnosis: Unipolar Depression  Resistant: Yes  Suicidal: No | Ketamine IV | Midazolam | NA - Single RCT | OR | Sample size: 72  Effect measure: 10.97 (0.61, 198.52)  p-value: Not reported | Het.: I²: NA, p=NA  Pub.B. Test: NA, p=NA |
| **Tolerability – Vertigo**  **(14-28 days)** | | | | | | | | |
| Jawad 2022 | | Diagnosis: Unipolar Depression  Resistant: Not reported in some of the RCTs  Suicidal: Mixed | Esketamine IN | Saline | Pooled RR | RR | Sample size: 855  Effect measure: 8.636 (4.525, 16.481)  p-value: 0.001 | Het.: I²: NA, p=NA  Pub.B. Test: NA, p=NA |
| **Tolerability – Vertigo**  **(>28 days)** | | | | | | | | |
| Jawad 2022 | | Diagnosis: Unipolar Depression  Resistant: Yes  Suicidal: No | Esketamine IN | Saline | NA - Single RCT | RR | Sample size: NA  Effect measure: 4.531 (2.189, 9.381)  p-value: 0.001 | Het.: I²: NA, p=NA  Pub.B. Test: NA, p=NA |
| **Tolerability – Vertigo**  **(Endpoint)** | | | | | | | | |
| An 2021 | | Diagnosis: Unipolar Depression  Resistant: Not reported in some of the RCTs  Suicidal: Mixed | Ketamine IN,Esketamine IN | Saline | Random Effect | RR | Sample size: 422  Effect measure: 7.04 (3.56, 13.93)  p-value: | Het.: I²: 0%, p=0.81  Pub.B. Test: NA, p=NA |
| Bahji 2022 | | Diagnosis: No Information  Resistant: No Information  Suicidal: No information | No Information | Saline,Midazolam | Random Effect | OR | Sample size: NA  Effect measure: 5.98 (3.36, 10.66)  p-value: Not reported | Het.: I²: 27%, p=NA  Pub.B. Test: Egger, p=NA |
| Zheng 2020 | | Diagnosis: Unipolar Depression  Resistant: Not reported in some of the RCTs  Suicidal: Mixed | Esketamine IN | Saline | Random Effect | RR | Sample size: 267  Effect measure: 9.78 (4.96, 19.27)  p-value: 0.00001 | Het.: I²: 0%, p=NA  Pub.B. Test: NA, p=NA |
| **Tolerability – Viral upper respiratory infection**  **(>28 days)** | | | | | | | | |
| Jawad 2022 | | Diagnosis: Unipolar Depression  Resistant: Yes  Suicidal: No | Esketamine IN | Saline | NA - Single RCT | RR | Sample size: NA  Effect measure: 0.874 (0.399, 1.919)  p-value: 0.738 | Het.: I²: NA, p=NA  Pub.B. Test: NA, p=NA |
| **Tolerability – Vomiting**  **(14-28 days)** | | | | | | | | |
| Jawad 2022 | | Diagnosis: Unipolar Depression  Resistant: Not reported in some of the RCTs  Suicidal: Mixed | Esketamine IN | Saline | Pooled RR | RR | Sample size: 855  Effect measure: 3.144 (1.794, 5.508)  p-value: 0.001 | Het.: I²: NA, p=NA  Pub.B. Test: NA, p=NA |
| **Tolerability – Vomiting**  **(>28 days)** | | | | | | | | |
| Jawad 2022 | | Diagnosis: Unipolar Depression  Resistant: Yes  Suicidal: No | Esketamine IN | Saline | NA - Single RCT | RR | Sample size: NA  Effect measure: 9.539 (1.237, 73.585)  p-value: 0.03 | Het.: I²: NA, p=NA  Pub.B. Test: NA, p=NA |
| **Tolerability – Vomiting**  **(24-48h)** | | | | | | | | |
| Bahji 2022 | | Diagnosis: Unipolar Depression  Resistant: No Information  Suicidal: No information | No Information | Saline | Random Effect | OR | Sample size: NA  Effect measure: 3.18 (1.8, 5.6)  p-value: Not reported | Het.: I²: %, p=NA  Pub.B. Test: Egger, p=NA |
| Zheng 2020 | | Diagnosis: Unipolar Depression  Resistant: No Information  Suicidal: No information | Esketamine IN | Saline | Random Effect | RR | Sample size: 746  Effect measure: 5.49 (2.4, 12.53)  p-value: 0.0001 | Het.: I²: 0%, p=NA  Pub.B. Test: NA, p=NA |

**Supplementary Table 5.** Extended outcomes table

IN: Intranasal; IV: Intravenous; MD: Mean Difference; NA: Not Available; OR: Odds Ratio; Pub.B.: Publication bias; RCT: Randomized Controlled Trial; RR: Relative Risk; SMD: Standardized Mean Difference; WMD: Weighted Mean Difference

## Supplementary Table 6. Risk of bias of the original studies

| **Study** | **Review** | **RoB Tool** | **RoB 1.0 - D1** | **RoB 1.0 - D2** | **RoB 1.0 - D3** | **RoB 1.0 - D4** | **RoB 1.0 - D5** | **RoB 1.0 - D6** | **RoB 1.0/2.0 - OB** |
| --- | --- | --- | --- | --- | --- | --- | --- | --- | --- |
| ***Arabzadeh 2018*** | Bahji 2022 | Cochrane RoB 1.0 (6D-1B) | Low | High | Low | Low | Low | Low | Low |
|  | Kryst 2020 | Cochrane RoB 1.0 (7D) | Low | Low | Low | Low | Low | Unclear | Unclear |
|  | Nuñez 2020 | Cochrane RoB 1.0 (7D) | Low | Low | Low | Low | High | Low | Unclear |
|  | **Review** | **RoB Tool** | **RoB 2.0 - D1** | | **RoB 2.0 - D2** | **RoB 2.0 - D4** | **RoB 2.0 - D3** | **RoB 2.0 - D5** | **RoB 1.0/2.0 - OB** |
|  | McIntyre 2020 | Cochrane RoB 2.0 | Low | | Low | Low | Low | Low | Low |
| ***Basso 2020*** | **Review** | **RoB Tool** | **RoB 2.0 - D1** | | **RoB 2.0 - D2** | **RoB 2.0 - D4** | **RoB 2.0 - D3** | **RoB 2.0 - D5** | **RoB 1.0/2.0 - OB** |
|  | Rhee 2022 | Cochrane RoB 2.0 | High | | Low | Low | Low | Low | NA |
| ***Berman 2000*** | **Review** | **RoB Tool** | **RoB 1.0 - D1** | **RoB 1.0 - D2** | **RoB 1.0 - D3** | **RoB 1.0 - D4** | **RoB 1.0 - D5** | **RoB 1.0 - D6** | **RoB 1.0/2.0 - OB** |
|  | Fond 2014 | Cochrane RoB 1.0 (4D-1B-?SR-?Oth) | Low | Low | Low | Low | Low | NA | NA |
|  | Bahji 2022 | Cochrane RoB 1.0 (6D-1B) | Low | Low | Low | Low | High | High | Low |
|  | Lee 2015 | Cochrane RoB 1.0 (6D-1B) | Low | Unclear | Low | Low | Unclear | Low | Low |
|  | Xu 2016 | Cochrane RoB 1.0 (6D-1B) | Unclear | Unclear | Unclear | Unclear | Low | Unclear | High |
|  | McGirr 2015 | Cochrane RoB 1.0 (6D-1R) | Unclear | Unclear | Low | High | Low | Low | Unclear |
|  | Caddy 2015 | Cochrane RoB 1.0 (7D) | Unclear | Unclear | Unclear | Unclear | High | High | Unclear |
|  | Kishimoto 2016 | Cochrane RoB 1.0 (7D) | Unclear | Unclear | Unclear | Unclear | Low | High | Low |
|  | Kryst 2020 | Cochrane RoB 1.0 (7D) | Unclear | Unclear | Unclear | Unclear | High | High | High |
|  | **Review** | **RoB Tool** | **RoB 2.0 - D1** | | **RoB 2.0 - D2** | **RoB 2.0 - D4** | **RoB 2.0 - D3** | **RoB 2.0 - D5** | **RoB 1.0/2.0 - OB** |
|  | McIntyre 2020 | Cochrane RoB 2.0 | Low | | Low | Low | High | Low | Low |
|  | **Review** | **RoB Tool** | **Other quality assesment tool** | |  |  |  |  |  |
|  | Marcantoni 2020 | Downs and Black checklist | 20 (good) | |  |  |  |  |  |
| ***Canuso 2018*** | **Review** | **RoB Tool** | **RoB 1.0 - D1** | **RoB 1.0 - D2** | **RoB 1.0 - D3** | **RoB 1.0 - D4** | **RoB 1.0 - D5** | **RoB 1.0 - D6** | **RoB 1.0/2.0 - OB** |
|  | Bahji 2022 | Cochrane RoB 1.0 (6D-1B) | Low | High | Low | Low | High | Low | Low |
|  | Dongjiao 2021 | Cochrane RoB 1.0 (7D) | Low | Low | Unclear | Low | High | Unclear | Unclear |
|  | Witt 2020 | Cochrane RoB 1.0 (7D) | Low | Low | Unclear | Unclear | Unclear | Low | Low |
|  | Zheng_2020 | Cochrane RoB 1.0 (7D) | Low | Low | Low | Low | Low | Low | High |
|  | **Review** | **RoB Tool** | **RoB 2.0 - D1** | | **RoB 2.0 - D2** | **RoB 2.0 - D4** | **RoB 2.0 - D3** | **RoB 2.0 - D5** | **RoB 1.0/2.0 - OB** |
|  | Jawad 2022 | Cochrane RoB 2.0 | Low | | Low | Low | Low | Low | NA |
|  | McIntyre 2020 | Cochrane RoB 2.0 | Low | | Low | Low | SC | Low | Unclear |
|  | Xiong 2021 | Cochrane RoB 2.0 | Low | | Low | SC | SC | Low | NA |
|  | **Review** | **RoB Tool** | **Other quality assesment tool** | |  |  |  |  |  |
|  | Papakostas 2020 | Jadad Score | 5/5 |  |  |  |  |  |  |
| ***Cao 2018*** | **Review** | **RoB Tool** | **RoB 1.0 - D1** | **RoB 1.0 - D2** | **RoB 1.0 - D3** | **RoB 1.0 - D4** | **RoB 1.0 - D5** | **RoB 1.0 - D6** | **RoB 1.0/2.0 - OB** |
|  | Bahji 2022 | Cochrane RoB 1.0 (6D-1B) | Low | Low | Low | Low | High | Low | Low |
|  | Kryst 2020 | Cochrane RoB 1.0 (7D) | Unclear | Unclear | Unclear | Unclear | High | High | High |
|  | **Review** | **RoB Tool** | **Other quality assesment tool** | |  |  |  |  |  |
|  | Marcantoni 2020 | Downs and Black checklist | 22 (good) | |  |  |  |  |  |
| ***Chen 2018*** | **Review** | **RoB Tool** | **RoB 1.0 - D1** | **RoB 1.0 - D2** | **RoB 1.0 - D3** | **RoB 1.0 - D4** | **RoB 1.0 - D5** | **RoB 1.0 - D6** | **RoB 1.0/2.0 - OB** |
|  | Bahji 2022 | Cochrane RoB 1.0 (6D-1B) | Low | Low | Low | Low | High | High | Low |
|  | Kryst 2020 | Cochrane RoB 1.0 (7D) | Unclear | Unclear | Unclear | Unclear | Low | High | Unclear |
|  | **Review** | **RoB Tool** | **RoB 2.0 - D1** | | **RoB 2.0 - D2** | **RoB 2.0 - D4** | **RoB 2.0 - D3** | **RoB 2.0 - D5** | **RoB 1.0/2.0 - OB** |
|  | McIntyre 2020 | Cochrane RoB 2.0 | Low | | Low | Low | SC | Low | Low |
|  | Xiong 2021 | Cochrane RoB 2.0 | Low | | Low | Low | SC | Low | NA |
|  | **Review** | **RoB Tool** | **Other quality assesment tool** | |  |  |  |  |  |
|  | Marcantoni 2020 | Downs and Black checklist | 29 (excellent) | |  |  |  |  |  |
| ***Daly 2018*** | **Review** | **RoB Tool** | **RoB 1.0 - D1** | **RoB 1.0 - D2** | **RoB 1.0 - D3** | **RoB 1.0 - D4** | **RoB 1.0 - D5** | **RoB 1.0 - D6** | **RoB 1.0/2.0 - OB** |
|  | Bahji 2022 | Cochrane RoB 1.0 (6D-1B) | Low | Low | Low | Low | High | Low | Low |
|  | Dongjiao 2021 | Cochrane RoB 1.0 (7D) | Low | Low | Unclear | Low | High | Unclear | Unclear |
|  | Zheng_2020 | Cochrane RoB 1.0 (7D) | Low | Low | Low | Unclear | Low | Low | High |
|  | **Review** | **RoB Tool** | **RoB 2.0 - D1** | | **RoB 2.0 - D2** | **RoB 2.0 - D4** | **RoB 2.0 - D3** | **RoB 2.0 - D5** | **RoB 1.0/2.0 - OB** |
|  | Jawad 2022 | Cochrane RoB 2.0 | Low | | Low | Low | Low | Low | NA |
|  | McIntyre 2020 | Cochrane RoB 2.0 | Low | | Low | Low | SC | Low | Unclear |
|  | **Review** | **RoB Tool** | **Other quality assesment tool** | |  |  |  |  |  |
|  | Papakostas 2020 | Jadad Score | 5/5 | |  |  |  |  |  |
| ***Diazgranados 2010*** | **Review** | **RoB Tool** | **RoB 1.0 - D1** | **RoB 1.0 - D2** | **RoB 1.0 - D3** | **RoB 1.0 - D4** | **RoB 1.0 - D5** | **RoB 1.0 - D6** | **RoB 1.0/2.0 - OB** |
|  | Fond 2014 | Cochrane RoB 1.0 (4D-1B-?SR-?Oth) | Low | Low | Low | Low | Low | NA | NA |
|  | Bahji 2022 | Cochrane RoB 1.0 (6D-1B) | Low | High | Low | Low | High | Low | Low |
|  | Lee 2015 | Cochrane RoB 1.0 (6D-1B) | Low | Unclear | Low | Low | Unclear | Low | Low |
|  | Xu 2016 | Cochrane RoB 1.0 (6D-1B) | Low | Unclear | Unclear | Unclear | High | Unclear | High |
|  | McGirr 2015 | Cochrane RoB 1.0 (6D-1R) | Low | Low | Low | High | Low | Low | Unclear |
|  | Bahji 2020 | Cochrane RoB 1.0 (7D) | Low | Low | Low | Low | Low | Low | High |
|  | Dean 2021 | Cochrane RoB 1.0 (7D) | Low | Unclear | Low | Unclear | Low | Unclear | Unclear |
|  | Fornaro 2020 | Cochrane RoB 1.0 (7D) | Low | Low | Low | Low | Unclear | Low | Low |
|  | Kishimoto 2016 | Cochrane RoB 1.0 (7D) | Low | Unclear | Unclear | Unclear | Low | High | Low |
|  | Kryst 2020 | Cochrane RoB 1.0 (7D) | Low | Unclear | Low | Unclear | Low | Unclear | High |
|  | **Review** | **RoB Tool** | **RoB 2.0 - D1** | | **RoB 2.0 - D2** | **RoB 2.0 - D4** | **RoB 2.0 - D3** | **RoB 2.0 - D5** | **RoB 1.0/2.0 - OB** |
|  | McIntyre 2020 | Cochrane RoB 2.0 | Low | | Low | Low | SC | Low | Low |
|  | **Review** | **RoB Tool** | **Other quality assesment tool** | |  |  |  |  |  |
|  | Marcantoni 2020 | Downs and Black checklist | 28 (excellent) | |  |  |  |  |  |
| ***Domany 2018*** | **Review** | **RoB Tool** | **RoB 1.0 - D1** | **RoB 1.0 - D2** | **RoB 1.0 - D3** | **RoB 1.0 - D4** | **RoB 1.0 - D5** | **RoB 1.0 - D6** | **RoB 1.0/2.0 - OB** |
|  | Bahji 2022 | Cochrane RoB 1.0 (6D-1B) | Low | Low | Low | Low | Low | Low | Low |
|  | Kryst 2020 | Cochrane RoB 1.0 (7D) | Unclear | High | Low | Low | Low | Unclear | Unclear |
|  | Nuñez 2020 | Cochrane RoB 1.0 (7D) | Low | Low | Low | Low | Low | Low | Unclear |
|  | **Review** | **RoB Tool** | **RoB 2.0 - D1** | | **RoB 2.0 - D2** | **RoB 2.0 - D4** | **RoB 2.0 - D3** | **RoB 2.0 - D5** | **RoB 1.0/2.0 - OB** |
|  | McIntyre 2020 | Cochrane RoB 2.0 | Low | | Low | Low | Low | Low | Low |
| ***Downey 2015*** | **Review** | **RoB Tool** | **RoB 1.0 - D1** | **RoB 1.0 - D2** | **RoB 1.0 - D3** | **RoB 1.0 - D4** | **RoB 1.0 - D5** | **RoB 1.0 - D6** | **RoB 1.0/2.0 - OB** |
|  | Bahji 2022 | Cochrane RoB 1.0 (6D-1B) | Low | Low | Low | Low | Low | Low | Low |
|  | Kryst 2020 | Cochrane RoB 1.0 (7D) | Unclear | Low | Low | Unclear | Low | Unclear | Unclear |
| ***Ekstrand 2022*** | **Review** | **RoB Tool** | **RoB 2.0 - D1** | | **RoB 2.0 - D2** | **RoB 2.0 - D4** | **RoB 2.0 - D3** | **RoB 2.0 - D5** | **RoB 1.0/2.0 - OB** |
|  | Rhee 2022 | Cochrane RoB 2.0 | Low | | SC | Low | SC | Low | NA |
| ***Fava 2018*** | **Review** | **RoB Tool** | **RoB 1.0 - D1** | **RoB 1.0 - D2** | **RoB 1.0 - D3** | **RoB 1.0 - D4** | **RoB 1.0 - D5** | **RoB 1.0 - D6** | **RoB 1.0/2.0 - OB** |
|  | Bahji 2022 | Cochrane RoB 1.0 (6D-1B) | Low | Low | Low | Low | High | Low | Low |
|  | Kryst 2020 | Cochrane RoB 1.0 (7D) | Low | Unclear | Low | Low | Low | Unclear | Unclear |
|  | **Review** | **RoB Tool** | **RoB 2.0 - D1** | | **RoB 2.0 - D2** | **RoB 2.0 - D4** | **RoB 2.0 - D3** | **RoB 2.0 - D5** | **RoB 1.0/2.0 - OB** |
|  | McIntyre 2020 | Cochrane RoB 2.0 | Low | | Low | Low | Low | Low | Low |
|  | **Review** | **RoB Tool** | **Other quality assesment tool** | |  |  |  |  |  |
|  | Marcantoni 2020 | Downs and Black checklist | 23 (good) | |  |  |  |  |  |
| ***Fedgchin 2019*** | **Review** | **RoB Tool** | **RoB 1.0 - D1** | **RoB 1.0 - D2** | **RoB 1.0 - D3** | **RoB 1.0 - D4** | **RoB 1.0 - D5** | **RoB 1.0 - D6** | **RoB 1.0/2.0 - OB** |
|  | Bahji 2022 | Cochrane RoB 1.0 (6D-1B) | Low | Low | Low | Low | Low | Low | Low |
|  | Dongjiao 2021 | Cochrane RoB 1.0 (7D) | Low | Low | Unclear | Unclear | Low | Unclear | High |
|  | Zheng_2020 | Cochrane RoB 1.0 (7D) | Low | Low | Low | Low | Low | Low | High |
|  | **Review** | **RoB Tool** | **RoB 2.0 - D1** | | **RoB 2.0 - D2** | **RoB 2.0 - D4** | **RoB 2.0 - D3** | **RoB 2.0 - D5** | **RoB 1.0/2.0 - OB** |
|  | Jawad 2022 | Cochrane RoB 2.0 | Low | | Low | Low | Low | Low | NA |
|  | McIntyre 2020 | Cochrane RoB 2.0 | Low | | Low | Low | Low | Low | Low |
|  | **Review** | **RoB Tool** | **Other quality assesment tool** | |  |  |  |  |  |
|  | Papakostas 2020 | Jadad Score | 5/5 | |  |  |  |  |  |
| ***Fu 2020*** | **Review** | **RoB Tool** | **RoB 2.0 - D1** | | **RoB 2.0 - D2** | **RoB 2.0 - D4** | **RoB 2.0 - D3** | **RoB 2.0 - D5** | **RoB 1.0/2.0 - OB** |
|  | Jawad 2022 | Cochrane RoB 2.0 | Low | | Low | Low | SC | Low | NA |
|  | Xiong 2021 | Cochrane RoB 2.0 | Low | | Low | SC | SC | Low | NA |
| ***Ghasemi 2014*** | **Review** | **RoB Tool** | **RoB 1.0 - D1** | **RoB 1.0 - D2** | **RoB 1.0 - D3** | **RoB 1.0 - D4** | **RoB 1.0 - D5** | **RoB 1.0 - D6** | **RoB 1.0/2.0 - OB** |
|  | Fond 2014 | Cochrane RoB 1.0 (4D-1B-?SR-?Oth) | Low | Low | Low | Low | Low | NA | NA |
|  | Caddy 2015 | Cochrane RoB 1.0 (7D) | Unclear | Unclear | Low | Low | Low | Low | Unclear |
|  | **Review** | **RoB Tool** | **RoB 2.0 - D1** | | **RoB 2.0 - D2** | **RoB 2.0 - D4** | **RoB 2.0 - D3** | **RoB 2.0 - D5** | **RoB 1.0/2.0 - OB** |
|  | Rhee 2022 | Cochrane RoB 2.0 | Low | | SC | Low | Low | Low | NA |
| ***Grunebaum 2017*** | **Review** | **RoB Tool** | **RoB 1.0 - D1** | **RoB 1.0 - D2** | **RoB 1.0 - D3** | **RoB 1.0 - D4** | **RoB 1.0 - D5** | **RoB 1.0 - D6** | **RoB 1.0/2.0 - OB** |
|  | Bahji 2022 | Cochrane RoB 1.0 (6D-1B) | Low | Low | Low | Low | High | High | Low |
|  | Dean 2021 | Cochrane RoB 1.0 (7D) | Low | Unclear | High | High | Low | High | Unclear |
|  | Witt 2020 | Cochrane RoB 1.0 (7D) | Low | Unclear | Unclear | Unclear | Low | Low | Low |
|  | **Review** | **RoB Tool** | **RoB 2.0 - D1** | | **RoB 2.0 - D2** | **RoB 2.0 - D4** | **RoB 2.0 - D3** | **RoB 2.0 - D5** | **RoB 1.0/2.0 - OB** |
|  | Xiong 2021 | Cochrane RoB 2.0 | Low | | SC | Low | Low | Low | NA |
| ***Grunebaum 2018*** | **Review** | **RoB Tool** | **RoB 1.0 - D1** | **RoB 1.0 - D2** | **RoB 1.0 - D3** | **RoB 1.0 - D4** | **RoB 1.0 - D5** | **RoB 1.0 - D6** | **RoB 1.0/2.0 - OB** |
|  | Bahji 2022 | Cochrane RoB 1.0 (6D-1B) | Low | Low | Low | Low | High | Low | Low |
|  | Kryst 2020 | Cochrane RoB 1.0 (7D) | Low | Unclear | Low | Low | Low | Unclear | Unclear |
|  | Witt 2020 | Cochrane RoB 1.0 (7D) | Low | Unclear | Low | Unclear | Unclear | Low | Low |
|  | **Review** | **RoB Tool** | **RoB 2.0 - D1** | | **RoB 2.0 - D2** | **RoB 2.0 - D4** | **RoB 2.0 - D3** | **RoB 2.0 - D5** | **RoB 1.0/2.0 - OB** |
|  | Xiong 2021 | Cochrane RoB 2.0 | Low | | Low | SC | SC | Low | NA |
|  | **Review** | **RoB Tool** | **Other quality assesment tool** |  |  |  |  |  |  |
|  | Marcantoni 2020 | Downs and Black checklist | 26 (excellent) |  |  |  |  |  |  |
| ***Hu 2016*** | **Review** | **RoB Tool** | **RoB 1.0 - D1** | **RoB 1.0 - D2** | **RoB 1.0 - D3** | **RoB 1.0 - D4** | **RoB 1.0 - D5** | **RoB 1.0 - D6** | **RoB 1.0/2.0 - OB** |
|  | Bahji 2022 | Cochrane RoB 1.0 (6D-1B) | Low | Low | Low | Low | Low | Low | Low |
|  | Kryst 2020 | Cochrane RoB 1.0 (7D) | Low | Unclear | Low | Low | Low | Unclear | Unclear |
|  | Witt 2020 | Cochrane RoB 1.0 (7D) | Unclear | Unclear | Low | Low | High | Low | Low |
|  | **Review** | **RoB Tool** | **RoB 2.0 - D1** | | **RoB 2.0 - D2** | **RoB 2.0 - D4** | **RoB 2.0 - D3** | **RoB 2.0 - D5** | **RoB 1.0/2.0 - OB** |
|  | McIntyre 2020 | Cochrane RoB 2.0 | Low | | Low | Low | Low | Low | Low |
|  | Xiong 2021 | Cochrane RoB 2.0 | Low | | Low | Low | SC | Low | NA |
| ***Ibrahim 2012*** | **Review** | **RoB Tool** | **RoB 2.0 - D1** | | **RoB 2.0 - D2** | **RoB 2.0 - D4** | **RoB 2.0 - D3** | **RoB 2.0 - D5** | **RoB 1.0/2.0 - OB** |
|  | McIntyre 2020 | Cochrane RoB 2.0 | Low | | Low | Low | SC | Low | Unclear |
| ***Ionescu 2019*** | **Review** | **RoB Tool** | **RoB 1.0 - D1** | **RoB 1.0 - D2** | **RoB 1.0 - D3** | **RoB 1.0 - D4** | **RoB 1.0 - D5** | **RoB 1.0 - D6** | **RoB 1.0/2.0 - OB** |
|  | Bahji 2022 | Cochrane RoB 1.0 (6D-1B) | Low | Low | Low | Low | Low | Low | Low |
|  | Kryst 2020 | Cochrane RoB 1.0 (7D) | Low | Low | Low | Low | Low | Unclear | Unclear |
|  | Witt 2020 | Cochrane RoB 1.0 (7D) | Low | Low | Unclear | Unclear | Low | Low | Low |
|  | **Review** | **RoB Tool** | **RoB 2.0 - D1** | | **RoB 2.0 - D2** | **RoB 2.0 - D4** | **RoB 2.0 - D3** | **RoB 2.0 - D5** | **RoB 1.0/2.0 - OB** |
|  | Xiong 2021 | Cochrane RoB 2.0 | Low | | Low | SC | Low | Low | NA |
| ***Ionescu 2021*** | **Review** | **RoB Tool** | **RoB 1.0 - D1** | **RoB 1.0 - D2** | **RoB 1.0 - D3** | **RoB 1.0 - D4** | **RoB 1.0 - D5** | **RoB 1.0 - D6** | **RoB 1.0/2.0 - OB** |
|  | Bahji 2022 | Cochrane RoB 1.0 (6D-1B) | Low | Low | Low | Low | High | Low | Low |
|  | **Review** | **RoB Tool** | **RoB 2.0 - D1** | | **RoB 2.0 - D2** | **RoB 2.0 - D4** | **RoB 2.0 - D3** | **RoB 2.0 - D5** | **RoB 1.0/2.0 - OB** |
|  | Jawad 2022 | Cochrane RoB 2.0 | Low | | Low | Low | SC | Low | NA |
| ***Jafairinia 2016*** | **Review** | **RoB Tool** | **RoB 1.0 - D1** | **RoB 1.0 - D2** | **RoB 1.0 - D3** | **RoB 1.0 - D4** | **RoB 1.0 - D5** | **RoB 1.0 - D6** | **RoB 1.0/2.0 - OB** |
|  | Nuñez 2020 | Cochrane RoB 1.0 (7D) | Low | Low | Low | Low | High | Low | Unclear |
|  | **Review** | **RoB Tool** | **RoB 2.0 - D1** | | **RoB 2.0 - D2** | **RoB 2.0 - D4** | **RoB 2.0 - D3** | **RoB 2.0 - D5** | **RoB 1.0/2.0 - OB** |
|  | McIntyre 2020 | Cochrane RoB 2.0 | Low | | Low | Low | Low | Low | Low |
| ***Kheirabadi 2019*** | **Review** | **RoB Tool** | **RoB 2.0 - D1** | | **RoB 2.0 - D2** | **RoB 2.0 - D4** | **RoB 2.0 - D3** | **RoB 2.0 - D5** | **RoB 1.0/2.0 - OB** |
|  | Rhee 2022 | Cochrane RoB 2.0 | Low | | SC | Low | Low | Low | NA |
| ***Kherabadi 2020*** | **Review** | **RoB Tool** | **RoB 2.0 - D1** | | **RoB 2.0 - D2** | **RoB 2.0 - D4** | **RoB 2.0 - D3** | **RoB 2.0 - D5** | **RoB 1.0/2.0 - OB** |
|  | Rhee 2022 | Cochrane RoB 2.0 | Low |  | SC | Low | Low | Low | NA |
| ***Kudoh 2002*** | **Review** | **RoB Tool** | **RoB 1.0 - D1** | **RoB 1.0 - D2** | **RoB 1.0 - D3** | **RoB 1.0 - D4** | **RoB 1.0 - D5** | **RoB 1.0 - D6** | **RoB 1.0/2.0 - OB** |
|  | Fond 2014 | Cochrane RoB 1.0 (4D-1B-?SR-?Oth) | Low | High | High | High | High | NA | NA |
|  | Witt 2020 | Cochrane RoB 1.0 (7D) | Low | Unclear | Unclear | Unclear | Unclear | Low | Low |
| ***Lai 2014*** | **Review** | **RoB Tool** | **RoB 1.0 - D1** | **RoB 1.0 - D2** | **RoB 1.0 - D3** | **RoB 1.0 - D4** | **RoB 1.0 - D5** | **RoB 1.0 - D6** | **RoB 1.0/2.0 - OB** |
|  | Bahji 2022 | Cochrane RoB 1.0 (6D-1B) | Low | Low | Low | Low | Low | High | Low |
|  | Xu 2016 | Cochrane RoB 1.0 (6D-1B) | Unclear | Unclear | Low | Low | Low | Unclear | High |
|  | Kishimoto 2016 | Cochrane RoB 1.0 (7D) | Unclear | Unclear | High | High | High | High | High |
| ***Lapidus 2014*** | **Review** | **RoB Tool** | **RoB 1.0 - D1** | **RoB 1.0 - D2** | **RoB 1.0 - D3** | **RoB 1.0 - D4** | **RoB 1.0 - D5** | **RoB 1.0 - D6** | **RoB 1.0/2.0 - OB** |
|  | Bahji 2022 | Cochrane RoB 1.0 (6D-1B) | Low | Low | Low | Low | Low | Low | Low |
|  | McGirr 2015 | Cochrane RoB 1.0 (6D-1R) | Low | Low | Low | Unclear | Low | Low | Unclear |
|  | Caddy 2015 | Cochrane RoB 1.0 (7D) | Low | Low | Low | Low | Low | Unclear | Unclear |
|  | Dongjiao 2021 | Cochrane RoB 1.0 (7D) | Low | Low | Low | High | Low | Unclear | Unclear |
|  | Kryst 2020 | Cochrane RoB 1.0 (7D) | Low | Low | Low | Low | Low | Unclear | Unclear |
| ***Lapidus 2014*** | **Review** | **RoB Tool** | **RoB 2.0 - D1** | | **RoB 2.0 - D2** | **RoB 2.0 - D4** | **RoB 2.0 - D3** | **RoB 2.0 - D5** | **RoB 1.0/2.0 - OB** |
|  | McIntyre 2020 | Cochrane RoB 2.0 | Low | | Low | Low | Low | Low | Low |
| ***Li 2016*** | **Review** | **RoB Tool** | **RoB 1.0 - D1** | **RoB 1.0 - D2** | **RoB 1.0 - D3** | **RoB 1.0 - D4** | **RoB 1.0 - D5** | **RoB 1.0 - D6** | **RoB 1.0/2.0 - OB** |
|  | Bahji 2022 | Cochrane RoB 1.0 (6D-1B) | Low | Low | Low | Low | High | Low | Low |
| ***Murrough 2013*** | **Review** | **RoB Tool** | **RoB 1.0 - D1** | **RoB 1.0 - D2** | **RoB 1.0 - D3** | **RoB 1.0 - D4** | **RoB 1.0 - D5** | **RoB 1.0 - D6** | **RoB 1.0/2.0 - OB** |
|  | Fond 2014 | Cochrane RoB 1.0 (4D-1B-?SR-?Oth) | Low | Low | Low | Low | Low | NA | NA |
|  | Bahji 2022 | Cochrane RoB 1.0 (6D-1B) | Low | Low | Low | Low | Low | Low | Low |
|  | Lee 2015 | Cochrane RoB 1.0 (6D-1B) | Low | Unclear | Low | Low | Unclear | Low | Low |
|  | Xu 2016 | Cochrane RoB 1.0 (6D-1B) | Unclear | Low | Low | Low | Low | Low | Low |
|  | McGirr 2015 | Cochrane RoB 1.0 (6D-1R) | Unclear | Low | Low | Unclear | Low | Low | Unclear |
|  | Caddy 2015 | Cochrane RoB 1.0 (7D) | Low | Low | Low | Low | Low | Unclear | Unclear |
|  | Kishimoto 2016 | Cochrane RoB 1.0 (7D) | Unclear | Unclear | Low | Low | Low | High | Low |
|  | Kryst 2020 | Cochrane RoB 1.0 (7D) | Unclear | Low | Low | Low | Low | Unclear | Unclear |
|  | Witt 2020 | Cochrane RoB 1.0 (7D) | Unclear | Low | Low | Low | Unclear | Low | Low |
|  | **Review** | **RoB Tool** | **RoB 2.0 - D1** | | **RoB 2.0 - D2** | **RoB 2.0 - D4** | **RoB 2.0 - D3** | **RoB 2.0 - D5** | **RoB 1.0/2.0 - OB** |
|  | McIntyre 2020 | Cochrane RoB 2.0 | Low | | Low | Low | Low | High | Unclear |
|  | Xiong 2021 | Cochrane RoB 2.0 | Low | | Low | Low | Low | Low | NA |
|  | **Review** | **RoB Tool** | **Other quality assesment tool** | |  |  |  |  |  |
|  | Marcantoni 2020 | Downs and Black checklist | 26 (excellent) | |  |  |  |  |  |
| ***Murrough 2015*** | **Review** | **RoB Tool** | **RoB 1.0 - D1** | **RoB 1.0 - D2** | **RoB 1.0 - D3** | **RoB 1.0 - D4** | **RoB 1.0 - D5** | **RoB 1.0 - D6** | **RoB 1.0/2.0 - OB** |
|  | Bahji 2022 | Cochrane RoB 1.0 (6D-1B) | Low | Low | Low | Low | Low | Low | Low |
|  | Kryst 2020 | Cochrane RoB 1.0 (7D) | Low | Unclear | Low | Low | Low | Unclear | Unclear |
|  | Witt 2020 | Cochrane RoB 1.0 (7D) | Low | Unclear | Unclear | Low | High | Low | Low |
|  | **Review** | **RoB Tool** | **RoB 2.0 - D1** | | **RoB 2.0 - D2** | **RoB 2.0 - D4** | **RoB 2.0 - D3** | **RoB 2.0 - D5** | **RoB 1.0/2.0 - OB** |
|  | McIntyre 2020 | Cochrane RoB 2.0 | Low | | Low | Low | Low | Low | Low |
|  | Xiong 2021 | Cochrane RoB 2.0 | Low | | SC | Low | SC | Low | NA |
|  | **Review** | **RoB Tool** | **Other quality assesment tool** | |  |  |  |  |  |
|  | Marcantoni 2020 | Downs and Black checklist | 14 (poor) | |  |  |  |  |  |
| ***Och-Ross 2019*** | **Review** | **RoB Tool** | **RoB 1.0 - D1** | **RoB 1.0 - D2** | **RoB 1.0 - D3** | **RoB 1.0 - D4** | **RoB 1.0 - D5** | **RoB 1.0 - D6** | **RoB 1.0/2.0 - OB** |
|  | Bahji 2022 | Cochrane RoB 1.0 (6D-1B) | Low | Low | Low | Low | Low | Low | Low |
|  | Dongjiao 2021 | Cochrane RoB 1.0 (7D) | Low | Low | Unclear | Unclear | High | High | Low |
|  | **Review** | **RoB Tool** | **RoB 2.0 - D1** | | **RoB 2.0 - D2** | **RoB 2.0 - D4** | **RoB 2.0 - D3** | **RoB 2.0 - D5** |  |
|  | Jawad 2022 | Cochrane RoB 2.0 | Low | | Low | Low | Low | Low | NA |
|  | **Review** | **RoB Tool** | **Other quality assesment tool** | |  |  |  |  |  |
|  | Papakostas 2020 | Jadad Score |  | |  |  |  |  |  |
| ***Phillips 2019*** | **Review** | **RoB Tool** | **RoB 1.0 - D1** | **RoB 1.0 - D2** | **RoB 1.0 - D3** | **RoB 1.0 - D4** | **RoB 1.0 - D5** | **RoB 1.0 - D6** | **RoB 1.0/2.0 - OB** |
|  | Bahji 2022 | Cochrane RoB 1.0 (6D-1B) | Low | Low | Low | Low | Low | Low | Low |
|  | **Review** | **RoB Tool** | **RoB 2.0 - D1** | | **RoB 2.0 - D2** | **RoB 2.0 - D4** | **RoB 2.0 - D3** | **RoB 2.0 - D5** | **RoB 1.0/2.0 - OB** |
|  | McIntyre 2020 | Cochrane RoB 2.0 | Low | | Low | Low | Low | Low | Low |
|  | Xiong 2021 | Cochrane RoB 2.0 | Low | | Low | Low | Low | Low | NA |
| ***Popova 2019*** | **Review** | **RoB Tool** | **RoB 1.0 - D1** | **RoB 1.0 - D2** | **RoB 1.0 - D3** | **RoB 1.0 - D4** | **RoB 1.0 - D5** | **RoB 1.0 - D6** | **RoB 1.0/2.0 - OB** |
|  | Bahji 2022 | Cochrane RoB 1.0 (6D-1B) | Low | Low | Low | Low | Low | Low | Low |
|  | Dongjiao 2021 | Cochrane RoB 1.0 (7D) | Low | Low | Unclear | High | Low | Unclear | Unclear |
|  | Zheng_2020 | Cochrane RoB 1.0 (7D) | Low | Low | Low | Low | Low | Low | High |
|  | **Review** | **RoB Tool** | **RoB 2.0 - D1** | | **RoB 2.0 - D2** | **RoB 2.0 - D4** | **RoB 2.0 - D3** | **RoB 2.0 - D5** | **RoB 1.0/2.0 - OB** |
|  | Jawad 2022 | Cochrane RoB 2.0 | Low | Low | Low | Low | Low | Low | NA |
|  | **Review** | **RoB Tool** | **Other quality assesment tool** | |  |  |  |  |  |
|  | Papakostas 2020 | Jadad Score | 5/5 | |  |  |  |  |  |
| ***Price 2014*** | **Review** | **RoB Tool** | **RoB 2.0 - D1** | | **RoB 2.0 - D2** | **RoB 2.0 - D4** | **RoB 2.0 - D3** | **RoB 2.0 - D5** | **RoB 1.0/2.0 - OB** |
|  | Xiong 2021 | Cochrane RoB 2.0 | SC | | Low | Low | Low | Low | NA |
| ***Sharma 2020*** | **Review** | **RoB Tool** | **RoB 2.0 - D1** | | **RoB 2.0 - D2** | **RoB 2.0 - D4** | **RoB 2.0 - D3** | **RoB 2.0 - D5** | **RoB 1.0/2.0 - OB** |
|  | Rhee 2022 | Cochrane RoB 2.0 | Low | | Low | Low | Low | Low | NA |
| ***Singh 2016*** | **Review** | **RoB Tool** | **RoB 1.0 - D1** | **RoB 1.0 - D2** | **RoB 1.0 - D3** | **RoB 1.0 - D4** | **RoB 1.0 - D5** | **RoB 1.0 - D6** | **RoB 1.0/2.0 - OB** |
|  | Bahji 2022 | Cochrane RoB 1.0 (6D-1B) | Low | Low | Low | Low | Low | Low | Low |
|  | Kishimoto 2016 | Cochrane RoB 1.0 (7D) | Unclear | Unclear | Unclear | Unclear | Low | High | High |
|  | Kryst 2020 | Cochrane RoB 1.0 (7D) | Low | Unclear | Low | Low | Low | Unclear | Unclear |
|  | Papadimitropoulou 2017 | Cochrane RoB 1.0 (7D) | NA | NA | NA | NA | NA | NA | NA |
|  | **Review** | **RoB Tool** | **RoB 2.0 - D1** | | **RoB 2.0 - D2** | **RoB 2.0 - D4** | **RoB 2.0 - D3** | **RoB 2.0 - D5** | **RoB 1.0/2.0 - OB** |
|  | McIntyre 2020 | Cochrane RoB 2.0 | Low | | Low | Low | SC | Low | Low |
| ***Sinyor 2018*** | **Review** | **RoB Tool** | **RoB 2.0 - D1** | | **RoB 2.0 - D2** | **RoB 2.0 - D4** | **RoB 2.0 - D3** | **RoB 2.0 - D5** | **RoB 1.0/2.0 - OB** |
|  | Xiong 2021 | Cochrane RoB 2.0 | SC | | Low | Low | Low | High | NA |
| ***Sos 2013*** | **Review** | **RoB Tool** | **RoB 1.0 - D1** | **RoB 1.0 - D2** | **RoB 1.0 - D3** | **RoB 1.0 - D4** | **RoB 1.0 - D5** | **RoB 1.0 - D6** | **RoB 1.0/2.0 - OB** |
|  | Fond 2014 | Cochrane RoB 1.0 (4D-1B-?SR-?Oth) | Low | Low | Low | Low | Low | NA | NA |
|  | Bahji 2022 | Cochrane RoB 1.0 (6D-1B) | Low | Low | Low | Low | Low | Low | Low |
|  | Lee 2015 | Cochrane RoB 1.0 (6D-1B) | Low | Unclear | Low | Low | Unclear | Low | Low |
|  | Xu 2016 | Cochrane RoB 1.0 (6D-1B) | Low | Unclear | Unclear | Unclear | High | High | High |
|  | McGirr 2015 | Cochrane RoB 1.0 (6D-1R) | Unclear | Unclear | Unclear | High | Low | Low | Unclear |
|  | Caddy 2015 | Cochrane RoB 1.0 (7D) | Low | Unclear | Unclear | Unclear | Low | Unclear | Unclear |
|  | Kishimoto 2016 | Cochrane RoB 1.0 (7D) | Unclear | Unclear | Unclear | Unclear | Low | Unclear | Low |
|  | Kryst 2020 | Cochrane RoB 1.0 (7D) | Low | Unclear | Unclear | Unclear | Low | Unclear | High |
|  | Witt 2020 | Cochrane RoB 1.0 (7D) | Low | Unclear | Unclear | Unclear | Low | Low | Low |
|  | **Review** | **RoB Tool** | **RoB 2.0 - D1** | | **RoB 2.0 - D2** | **RoB 2.0 - D4** | **RoB 2.0 - D3** | **RoB 2.0 - D5** | **RoB 1.0/2.0 - OB** |
|  | McIntyre 2020 | Cochrane RoB 2.0 | Low | | Low | Low | SC | Low | Low |
|  | **Review** | **RoB Tool** | **Other quality assesment tool** | |  |  |  |  |  |
|  | Marcantoni 2020 | Downs and Black checklist | 25 (good) | |  |  |  |  |  |
| ***Su 2017*** | **Review** | **RoB Tool** | **RoB 1.0 - D1** | **RoB 1.0 - D2** | **RoB 1.0 - D3** | **RoB 1.0 - D4** | **RoB 1.0 - D5** | **RoB 1.0 - D6** | **RoB 1.0/2.0 - OB** |
|  | Bahji 2022 | Cochrane RoB 1.0 (6D-1B) | Low | Low | Low | Low | Low | Low | Low |
|  | Kryst 2020 | Cochrane RoB 1.0 (7D) | Unclear | Unclear | Unclear | Unclear | High | Unclear | Unclear |
|  | Witt 2020 | Cochrane RoB 1.0 (7D) | Unclear | Unclear | Unclear | Unclear | High | Low | Low |
|  | **Review** | **RoB Tool** | **RoB 2.0 - D1** | | **RoB 2.0 - D2** | **RoB 2.0 - D4** | **RoB 2.0 - D3** | **RoB 2.0 - D5** | **RoB 1.0/2.0 - OB** |
|  | McIntyre 2020 | Cochrane RoB 2.0 | Low | | Low | Low | Low | Low | Low |
|  | **Review** | **RoB Tool** | **Other quality assesment tool** | |  |  |  |  |  |
|  | Marcantoni 2020 | Downs and Black checklist | 17 (fair) | |  |  |  |  |  |
| ***Takahashi 2021*** | **Review** | **RoB Tool** | **RoB 2.0 - D1** | | **RoB 2.0 - D2** | **RoB 2.0 - D4** | **RoB 2.0 - D3** | **RoB 2.0 - D5** | **RoB 1.0/2.0 - OB** |
|  | Jawad 2022 | Cochrane RoB 2.0 | Low | | Low | Low | Low | Low | NA |
| ***Zarate 2006*** | **Review** | **RoB Tool** | **RoB 1.0 - D1** | **RoB 1.0 - D2** | **RoB 1.0 - D3** | **RoB 1.0 - D4** | **RoB 1.0 - D5** | **RoB 1.0 - D6** | **RoB 1.0/2.0 - OB** |
|  | Fond 2014 | Cochrane RoB 1.0 (4D-1B-?SR-?Oth) | Low | Low | Low | Low | Low | NA | NA |
|  | Bahji 2022 | Cochrane RoB 1.0 (6D-1B) | Low | Low | Low | Low | Low | Low | Low |
|  | Lee 2015 | Cochrane RoB 1.0 (6D-1B) | Low | Unclear | Low | Low | Unclear | Low | Low |
|  | Xu 2016 | Cochrane RoB 1.0 (6D-1B) | Low | Unclear | Unclear | Unclear | High | Unclear | High |
|  | McGirr 2015 | Cochrane RoB 1.0 (6D-1R) | Low | Low | Low | High | Low | Low | Unclear |
|  | Caddy 2015 | Cochrane RoB 1.0 (7D) | Low | Unclear | Low | Unclear | Unclear | Unclear | Unclear |
|  | Kishimoto 2016 | Cochrane RoB 1.0 (7D) | Low | Unclear | Unclear | Unclear | Low | High | Low |
|  | Kryst 2020 | Cochrane RoB 1.0 (7D) | Low | Unclear | Low | Unclear | Low | Unclear | High |
|  | **Review** | **RoB Tool** | **RoB 2.0 - D1** | | **RoB 2.0 - D2** | **RoB 2.0 - D4** | **RoB 2.0 - D3** | **RoB 2.0 - D5** | **RoB 1.0/2.0 - OB** |
|  | McIntyre 2020 | Cochrane RoB 2.0 | Low | | Low | Low | SC | Low | Low |
|  | **Review** | **RoB Tool** | **Other quality assesment tool** | |  |  |  |  |  |
|  | Marcantoni 2020 | Downs and Black checklist | 25 (good) | |  |  |  |  |  |
| ***Zarate 2012*** | **Review** | **RoB Tool** | **RoB 1.0 - D1** | **RoB 1.0 - D2** | **RoB 1.0 - D3** | **RoB 1.0 - D4** | **RoB 1.0 - D5** | **RoB 1.0 - D6** | **RoB 1.0/2.0 - OB** |
|  | Fond 2014 | Cochrane RoB 1.0 (4D-1B-?SR-?Oth) | Low | Low | Low | Low | Low | NA | NA |
|  | Bahji 2022 | Cochrane RoB 1.0 (6D-1B) | Low | Low | Low | Low | Low | Low | Low |
|  | Lee 2015 | Cochrane RoB 1.0 (6D-1B) | Low | Unclear | Low | Low | Unclear | Low | Low |
|  | Xu 2016 | Cochrane RoB 1.0 (6D-1B) | Low | Unclear | Unclear | Unclear | High | Unclear | High |
|  | McGirr 2015 | Cochrane RoB 1.0 (6D-1R) | Low | Low | Low | High | Low | Low | Unclear |
|  | Bahji 2020 | Cochrane RoB 1.0 (7D) | Low | Low | Low | Low | Low | Low | High |
|  | Caddy 2015 | Cochrane RoB 1.0 (7D) | Low | Unclear | Low | Low | Low | Unclear | Unclear |
|  | Dean 2021 | Cochrane RoB 1.0 (7D) | Low | Unclear | Low | Unclear | Low | Unclear | Unclear |
|  | Fornaro 2020 | Cochrane RoB 1.0 (7D) | Low | Low | Low | Low | Unclear | Low | Low |
|  | Kishimoto 2016 | Cochrane RoB 1.0 (7D) | Low | Unclear | Unclear | Unclear | Low | High | Low |
|  | Kryst 2020 | Cochrane RoB 1.0 (7D) | Low | Unclear | Low | Unclear | Low | Unclear | High |
|  | Witt 2020 | Cochrane RoB 1.0 (7D) | Low | Unclear | Low | Unclear | Low | Low | Low |
|  | **Review** | **RoB Tool** | **RoB 2.0 - D1** | | **RoB 2.0 - D2** | **RoB 2.0 - D4** | **RoB 2.0 - D3** | **RoB 2.0 - D5** | **RoB 1.0/2.0 - OB** |
|  | McIntyre 2020 | Cochrane RoB 2.0 | Low | | Low | Low | Low | Low | Low |
|  | Xiong 2021 | Cochrane RoB 2.0 | Low | | Low | Low | Low | Low | NA |

## Supplementary Table 7. Systematic reviews’ quality assessment (AMSTAR 2)

|  | Q1 | Q2 | Q3 | Q4 | Q5 | Q6 | Q7 | Q8 | Q9 | Q10 | Q11 | Q12 | Q13 | Q14 | Q15 | Q16 | Overall score |
| --- | --- | --- | --- | --- | --- | --- | --- | --- | --- | --- | --- | --- | --- | --- | --- | --- | --- |
| Bahji 2020 (1) | 1 | 0 | 1 | 1 | 1 | 1 | 0 | 1 | 1 | 0 | 1 | 1 | 1 | 1 | 1 | 1 | Critically low |
| Bahji 2022 (2) | 1 | 0 | 1 | 0 | 1 | 1 | 0 | 0 | 1 | 0 | 0 | 0 | 1 | 0 | 1 | 1 | Critically low |
| Caddy 2015 (3) | 1 | 1 | 1 | 1 | 1 | 1 | 1 | 1 | 1 | 0 | 1 | 1 | 1 | 1 | 0 | 1 | Low |
| Dean 2021 (4) | 1 | 1 | 1 | 1 | 1 | 1 | 1 | 1 | 1 | 0 | 1 | 1 | 1 | 1 | 0 | 1 | Low |
| Dongjiao 2021 (5) | 1 | 0 | 1 | 0 | 0 | 1 | 1 | 1 | 1 | 0 | 0 | 0 | 0 | 0 | 0 | 1 | Critically low |
| Fond 2014 (6) | 0 | 0 | 1 | 1 | 1 | 1 | 0 | 0 | 1 | 0 | 0 | 1 | 1 | 1 | 1 | 0 | Critically low |
| Fornaro 2020 (7) | 1 | 1 | 1 | 1 | 1 | 1 | 1 | 1 | 1 | 0 | 1 | 0 | 0 | 1 | 0 | 1 | Critically low |
| Han 2016 (8) | 1 | 0 | 1 | 1 | 1 | 1 | 0 | 1 | 0 | 0 | 0 | 0 | 0 | 1 | 1 | 1 | Critically low |
| Hock 2022 (9) | 1 | 0 | 0 | 0 | 0 | 1 | 0 | 1 | 0 | 0 | 0 | 0 | 0 | 0 | 0 | 1 | Critically low |
| Jawad 2022 (10) | 1 | 0 | 0 | 1 | 1 | 1 | 0 | 0 | 1 | 0 | 0 | 1 | 1 | 0 | 1 | 0 | Critically low |
| Kishimoto 2016 (11) | 1 | 0 | 1 | 1 | 1 | 1 | 0 | 1 | 1 | 1 | 0 | 0 | 0 | 0 | 1 | 1 | Critically low |
| Kryst 2020 (12) | 1 | 0 | 1 | 0 | 1 | 0 | 0 | 1 | 1 | 0 | 1 | 1 | 1 | 1 | 0 | 1 | Critically low |
| Lee 2015 (13) | 1 | 0 | 1 | 0 | 1 | 1 | 0 | 1 | 0 | 0 | 0 | 1 | 1 | 1 | 1 | 1 | Critically low |
| Marcantoni 2020 (14) | 1 | 0 | 0 | 1 | 1 | 1 | 1 | 1 | 1 | 0 | 1 | 1 | 1 | 1 | 1 | 1 | Low |
| McGirr 2015 (15) | 1 | 0 | 1 | 0 | 0 | 1 | 1 | 1 | 0 | 0 | 1 | 1 | 1 | 1 | 1 | 1 | Critically low |
| McIntyre 2020 (16) | 1 | 0 | 1 | 0 | 0 | 0 | 0 | 0 | 1 | 0 | 1 | 0 | 0 | 1 | 1 | 1 | Critically low |
| Newport 2015 (17) | 1 | 0 | 1 | 0 | 0 | 0 | 0 | 1 | 0 | 0 | 0 | 0 | 0 | 0 | 0 | 0 | Critically low |
| Nuñez 2020 (18) | 1 | 1 | 0 | 1 | 1 | 1 | 0 | 1 | 1 | 0 | 0 | 0 | 0 | 0 | 0 | 0 | Critically low |
| Papadimitropoulou 2017 (19) | 1 | 0 | 1 | 0 | 0 | 0 | 0 | 0 | 1 | 0 | 1 | 0 | 0 | 0 | 0 | 1 | Critically low |
| Papakostas 2020 (20) | 1 | 0 | 1 | 0 | 0 | 1 | 0 | 1 | 0 | 0 | 0 | 0 | 0 | 0 | 1 | 1 | Critically low |
| Rhee 2022 (21) | 1 | 0 | 0 | 0 | 1 | 1 | 0 | 1 | 1 | 0 | 1 | 0 | 0 | 0 | 1 | 1 | Critically low |
| Romeo 2015 (22) | 1 | 0 | 1 | 0 | 0 | 0 | 0 | 1 | 0 | 0 | 1 | 0 | 0 | 0 | 1 | 0 | Critically low |
| Witt 2020 (23) | 1 | 1 | 1 | 1 | 1 | 1 | 0 | 1 | 1 | 0 | 1 | 1 | 0 | 0 | 0 | 1 | Critically low |
| Xiong 2021 (24) | 1 | 0 | 1 | 1 | 1 | 0 | 0 | 1 | 1 | 0 | 1 | 0 | 1 | 1 | 1 | 1 | Critically low |
| Xu 2016 (25) | 1 | 0 | 1 | 0 | 1 | 1 | 0 | 1 | 1 | 0 | 0 | 0 | 0 | 0 | 0 | 1 | Critically low |
| Zheng 2020 (26) | 1 | 0 | 1 | 1 | 1 | 1 | 0 | 0 | 1 | 0 | 0 | 0 | 0 | 0 | 1 | 1 | Critically low |
| % of positive answers | 96% | 19% | 80% | 50% | 69% | 77% | 23% | 77% | 73% | 4% | 50% | 38% | 42% | 42% | 58% | 81% |  |
| N. of positive answers | 25 | 5 | 21 | 13 | 18 | 20 | 6 | 20 | 19 | 1 | 13 | 11 | 11 | 11 | 15 | 21 |  |

Item 1: Did the research questions and inclusion criteria for the review include the components of PICO? Item 2: Did the report of the review contain an explicit statement that the review methods were established prior to the conduct of the review and did the report justify any significant deviations from the protocol? Item 3: Did the review authors explain their selection of the study designs for inclusion in the review? Item 4: Did the review authors use a comprehensive literature search strategy? Item 5: Did the review authors perform study selection in duplicate? Item 6: Did the review authors perform data extraction in duplicate? Item 7: Did the review authors provide a list of excluded studies and justify the exclusions? Item 8: Did the review authors describe the included studies in adequate detail? Item 9: Did the review authors use a satisfactory technique for assessing the risk of bias (RoB) in individual studies that were included in the review? Item 10: Did the review authors report on the sources of funding for the studies included in the review? Item 11: If meta-analysis was performed did the review authors use appropriate methods for statistical combination of results? Item 12: If meta-analysis was performed, did the review authors assess the potential impact of RoB in individual studies on the results of the meta-analysis or other evidence synthesis? Item 13: Did the review authors account for RoB in individual studies when interpreting/ discussing the results of the review? Item 14: Did the review authors provide a satisfactory explanation for, and discussion of, any heterogeneity observed in the results of the review? Item 15: If they performed quantitative synthesis did the review authors carry out an adequate investigation of publication bias (small study bias) and discuss its likely impact on the results of the review? Item 16: Did the review authors report any potential sources of conflict of interest, including any funding they received for conducting the review?

1. A. B, Ermacora D, Stephenson C, Hawken ER, Vazquez. Comparative Efficacy and Tolerability of Adjunctive Pharmacotherapies for Acute Bipolar Depression: A Systematic Review and Network Meta-analysis. Canadian journal of psychiatry Revue canadienne de psychiatrie. 2021;66(3).

2. Gh, A. B, Zarate CA, Vazquez. Efficacy and safety of racemic ketamine and esketamine for depression: a systematic review and meta-analysis. Expert opinion on drug safety. 2022;21(6).

3. C. C, Amit BH, McCloud TL, Rendell JM, Furukawa TA, McShane R, et al. Ketamine and other glutamate receptor modulators for depression in adults. The Cochrane database of systematic reviews. 2015(9).

4. L. DR, Marquardt T, Hurducas C, Spyridi S, Barnes A, Smith R, et al. Ketamine and other glutamate receptor modulators for depression in adults with bipolar disorder. The Cochrane database of systematic reviews. 2021;10(10).

5. D. A, Wei C, Wang J, Wu. Intranasal Ketamine for Depression in Adults: A Systematic Review and Meta-Analysis of Randomized, Double-Blind, Placebo-Controlled Trials. Frontiers in psychology. 2021;12.

6. Fond G, Loundou A, Rabu C, Macgregor A, Lançon C, Brittner M, et al. Ketamine administration in depressive disorders: a systematic review and meta-analysis. Psychopharmacology. 2014;231(18):3663-76.

7. M. F, Carvalho AF, Fusco A, Anastasia A, Solmi M, Berk M, et al. The concept and management of acute episodes of treatment-resistant bipolar disorder: a systematic review and exploratory meta-analysis of randomized controlled trials. Journal of affective disorders. 2020;276.

8. Y. H, Chen J, Zou D, Zheng P, Li Q, Wang H, et al. Efficacy of ketamine in the rapid treatment of major depressive disorder: a meta-analysis of randomized, double-blind, placebo-controlled studies. Neuropsychiatric disease and treatment. 2016;12.

9. Gi, S. HR, Feeney A, Iovieno N, Murrough JW, Mathew SJ, et al. Rapidity of Symptom Improvement With Intranasal Esketamine for Major Depressive Disorder: A Systematic Review and Meta-Analysis. The Journal of clinical psychiatry. 2022;84(1).

10. McIntyre MYJ, Joshua DDV, Felicia C, Saja J, Leanna MWL, Emily SG, et al. The efficacy and safety of adjunctive intranasal esketamine treatment in major depressive disorder: a systematic review and meta-analysis. <https://doiorg/101080/1474033820222058488>. 2022.

11. Cu, T. K, Chawla JM, Hagi K, Zarate CA, Kane JM, et al. Single-dose infusion ketamine and non-ketamine N-methyl-d-aspartate receptor antagonists for unipolar and bipolar depression: a meta-analysis of efficacy, safety and time trajectories. Psychological medicine. 2016;46(7).

12. J. K, Kawalec P, Mitoraj AM, Pilc A, Lasoń W, Brzostek. Efficacy of single and repeated administration of ketamine in unipolar and bipolar depression: a meta-analysis of randomized clinical trials. Pharmacological reports : PR. 2020;72(3).

13. E. LE, Della Selva MP, Liu A, Himelhoch. Ketamine as a novel treatment for major depressive disorder and bipolar depression: a systematic review and quantitative meta-analysis. General hospital psychiatry. 2015;37(2).

14. S. MW, Akoumba BS, Wassef M, Mayrand J, Lai H, Richard-Devantoy S, et al. A systematic review and meta-analysis of the efficacy of intravenous ketamine infusion for treatment resistant depression: January 2009 - January 2019. Journal of affective disorders. 2020;277.

15. Rw, A. M, Berlim MT, Bond DJ, Fleck MP, Yatham LN, et al. A systematic review and meta-analysis of randomized, double-blind, placebo-controlled trials of ketamine in the rapid treatment of major depressive episodes. Psychological medicine. 2015;45(4).

16. Er, Subramaniapillai M, Mansur R, Ho R, Lam RW, Rosenblat JD, et al. The effect of intravenous, intranasal, and oral ketamine in mood disorders: A meta-analysis. Journal of affective disorders. 2020;276.

17. J. ND, Carpenter LL, McDonald WM, Potash JB, Tohen M, Nemeroff CB. Ketamine and Other NMDA Antagonists: Early Clinical Trials and Possible Mechanisms in Depression. The American journal of psychiatry. 2015;172(10).

18. A. NN, Joseph B, Pahwa M, Seshadri A, Prokop LJ, Kung S, et al. An Update on the Efficacy and Tolerability of Oral Ketamine for Major Depression: A Systematic Review and Meta-Analysis. Psychopharmacology bulletin. 2020;50(4).

19. Kubitz KP, Carla V, Andreas K, Christina D, Nicole. Comparative efficacy and tolerability of pharmacological and somatic interventions in adult patients with treatment-resistant depression: a systematic review and network meta-analysis. <https://doiorg/101080/0300799520161277201>. 2017.

20. I. PG, Salloum NC, Hock RS, Jha MK, Murrough JW, Mathew SJ, et al. Efficacy of Esketamine Augmentation in Major Depressive Disorder: A Meta-Analysis. The Journal of clinical psychiatry. 2020;81(4).

21. St, G. RT, Shim SR, Forester BP, Nierenberg AA, McIntyre RS, et al. Efficacy and Safety of Ketamine vs Electroconvulsive Therapy Among Patients With Major Depressive Episode: A Systematic Review and Meta-analysis. JAMA psychiatry. 2022;79(12).

22. Jy, B. R, Choucha W, Fossati P, Rotge. Meta-analysis of short- and mid-term efficacy of ketamine in unipolar and bipolar depression. Psychiatry research. 2015;230(2).

23. K. W, Potts J, Hubers A, Grunebaum MF, Murrough JW, Loo C, et al. Ketamine for suicidal ideation in adults with psychiatric disorders: A systematic review and meta-analysis of treatment trials. The Australian and New Zealand journal of psychiatry. 2020;54(1).

24. Rs, J. X, Lipsitz O, Chen-Li D, Rosenblat JD, Rodrigues NB, et al. The acute antisuicidal effects of single-dose intravenous ketamine and intranasal esketamine in individuals with major depression and bipolar disorders: A systematic review and meta-analysis. Journal of psychiatric research. 2021;134.

25. Y. X, Hackett M, Carter G, Loo C, Gálvez V, Glozier N, et al. Effects of Low-Dose and Very Low-Dose Ketamine among Patients with Major Depression: a Systematic Review and Meta-Analysis. The international journal of neuropsychopharmacology. 2016;19(4).

26. Yt, W. Z, Cai DB, Xiang YQ, Zheng W, Jiang WL, et al. Adjunctive intranasal esketamine for major depressive disorder: A systematic review of randomized double-blind controlled-placebo studies. Journal of affective disorders. 2020;265.

## Supplementary Table 8. Main analysis

| **Outcome** | **<60 mins** | **60-90 mins** | **90-120 mins** | **120-240 mins** | **24-48 hours** | **3-6 days** | **7-13 days** | **14-28 days** | **>28 days** | **Endpoint** |
| --- | --- | --- | --- | --- | --- | --- | --- | --- | --- | --- |
| **Lack of efficacy** | **U., B., M.:** No information | **U., B., M.:** No information | **U., B., M.:** No information | **U., B., M.:** No information | **U., B., M.:** No information | **U., B., M.:** No information | **U., B., M.:** No information | **U., B., M.:** No information | **U., B., M.:** No information | **U:** 1 SR, 1 /1 MA K ≅ C, G-H  **B:** 1 SR, 1/1 MA K ≅ C, G-NA  **M:** No information |
| **Total dropouts** | **U., B., M.:** No information | **U., B., M.:** No information | **U., B., M.:** No information | **U., B., M.:** No information | **U., B., M.:** No information | **U., B., M.:** No information | **U., B., M.:** No information | **U., B., M.:** No information | B:  1 SR, 1/1 MA K ≅ C, G-NA  U., M.: No information | **U:** 2 SR, 3/3 MA K ≅ C, 1/3 G-H, 1 G-NA  **B:** 2 SR, 2/2 MA K ≅ C, 2 G-L  **M:** 2 SR, 2/2 MA K ≅ C, 2/2 G-NA |
| **Depressive symptoms** | **U., B.:** No information  **M:** 1 SR, 1/1 MA K ≅ C, G-NA | **U., B.:** No information  **M:** 1 SR, 1/1 MA K > C, G-NA | **U., B.:** No information  **M:** 1 SR, 1/1 MA K > C, G-NA | **U**: 1 SR, 1/1 MA K > C, G-NA  **B**: No information  **M**: 1 SR, 1/1 MA K > C, H-NA | **U:** 7 SR, 11/11 MA K > C, 1/11 G-L, 10/11 G-NA  **B:** 4 SR, 4/5 MA K > C, 1/5 MA K ≅ C, 1/5 G-VL, 4/5  G-NA  **M:** 5 SR, 11/11 MA K > C, 11 G-NA | **U**: 3 SR, 4/5 MA K > C, 1/5 MA K ≅ C, 5 G-NA  **B**: 2 SR, 2 MA K > C, G-NA  **M**: 3 SR, 6/6 MA K > C, 6 G-NA | **U**: 5 SR,MA 6/8 K > C, 2/6 K ≅ C, 8/8 G-NA  **B**: 4 SR, 4/4 MA K ≅ C, 1/4 G-VL, 3/4 G-NA  **M**: 2 SR, 2/2 MA K > C, G-NA | **U**: 6 SR, 9/10 K > C, 1/10 K ≅ C, 10/10 G-NA  **B**: 4 SR, 4 MA K ≅ C, 4/4 G-NA  **M**: No information | **U., B., M.:** No information | **U**: 6 SR, 9 MA K > C, 1/9 G-H, 8/9 G-NA  **B**: 1 SR, 1/1 MA K > C, 1/1 G-NA  **M**: 4 SR, 5 MA K > C, 5/5 G-NA |
| **Remission** | **U**: No information  **B**: 1 SR, 1/1 MA K ≅ C, G-NA  **M**: 1 SR, 1/1 MA K ≅ C, G-NA | **U., B.:** No information  **M**: 2 SR, 2/2 MA K > C, G-NA | **U**: 1 SR, 1 MA K > C, G-H  **B**: No information  **M**: 2 SR, 2/2 K > C, 2/2 G-NA | **U., B.:** No information  **M**: 2 SR, 2 MA K > C, 2/2 H-NA | **U**: 5 SR, 4/8 MA K > C, 4/8 MA K ≅ C, 1/8 G-H, 7/8 NA  **B**: 1 SR, 2/2 MA K ≅ C, 1 G-VL, 1 G-NA  **M**: 6 SR, 8/8 K > C, G-NA | **U**: 2 SR, 2/4 K > C, 2/4 K ≅ C, 4/4 H-NA  **B**: 1 SR, 1/1 K ≅ C, 1/1 G-NA  **M**: 4 SR, 4/4 K > C, 4/4 G-NA | **U**: 3 SR, 2/4 MA K > C, 2/4 MA K ≅ C, 1/4 G-H, 3/4 G-NA  **B**: 2 SR, 2/2 MA K ≅ C, 1/2 G-VL, 1/2 G-NA  **M**: 5 SR, MA 2/5 K > C, MA 3/5 K ≅ C, G-NA | **U**: 3 SR, 2/3 MA K > C, 1/3 MA K ≅ C, 1/3 G-H, 2/3 G-NA  **B**: 1 SR, 1/1 MA K ≅ C, G-NA  **M**: 1 SR, 1/1 K ≅ C, 1/1 G-NA | **U**: 1 SR, 1/1 MA K > C, 1/1 G-NA  **B., M.**: No information | **U**: 5 SR, 4/5 MA K > C, 1/5 MA K ≅ C, 1/5 G-H, 4/5 G-NA  **B**: 1 SR, 1/1 K > C, G-NA  **M**: 1 SR, 1/1 K > C, G-NA |
| **Response** | **U., B.:** No information  **M**: 2 SR, 2/2 MA K > C, G-NA | **U., B.:** No information  **M**: 2 SR, 2/2 MA K > C, G-NA | **U**: 1 SR, 1/1 MA K > C, G-H  **B**: No information  **M**: 2 SR, 2/2 MA K > C, G-NA | **U., B.:** No information  **M**: 1 SR, 1/1 MA K > C, G-NA | **U**: 6 SR, 8/8 MA K > C, 1/8 G-H, 2/8 G-L, 5/8 G-NA  **B**: 3 SR, 4/4K > , 1/4 G-L, 1/4 G-VL, 2/4 G-NA  **M**: 5 SR, 11/11 K > C, G-NA | **U**: 3 SR, 5/5 MA K > C, 2/5 G-L, 3/5 G-NA  **B**: 1 SR, 1/1 MA K ≅ C, G-NA  **M**: 4 SR, 5/5 MA K > C, 5/5 G-NA | **U**: 5 SR, 6/6 K > C, 1/6 G-H, 1/6 G-L, 4/6 G-NA  **B**: 2 SR, 2/2 MA K ≅ C, G-NA  **M**: 5 SR, 7/7 MA K > C, G-NA | **U**: 5 SR, 7/8 MA K > C, 1/8 MA K ≅ C, 2/8 G-H, 6/8 G-NA  **B**: 1 SR, 1/1 MA K ≅ C, 1/1 G-NA  **M**: 1 SR, 1/1 MA K ≅ C, G-NA | **U., B., M.:** No information | **U**: 4 SR, 3/4 MA K > C, 1/4 MA K ≅ C, 1/4 G-H, 3/4 G-NA  **B**: 1 SR, 1/1 K > C, G-NA  **M**: 1 SR, 1/1 K > C, G-NA |
| **Suicidal ideation** | **U., B., M.:** No information | **U., B., M.:** No information | **U., B., M.:** No information | **U., B., M.:** No information | **U., B., M.:** No information | **U., B., M.:** No information | **U., B., M.:** No information | **U:** 1 SR, 1 MA K ≅ C, G-NA  **B., M.:** No information | **U., B., M.:** No information | **U., B., M.:** No information |
| **Suicide scales** | **U., B.:** No information  **M**: 1 SR, 1 MA K ≅ C, G-M | **U., B., M.:** No information | **U., B., M.:** No information | **U**: 1 SR, 2 MA K > C, G-NA  **B**: No information  **M**: 1 SR, 2 MA K > C, G-NA | **U**: 1 SR, 1 MA K > C, G-NA  **B**: 1 SR, 1 MA K ≅ C, G-NA  **M**: 1 SR, 2 MA K > C, H-H | **U., B.:** No information  **M**: 1 SR, 1 MA K > C, G-L | **U., B., M.:** No information | **U., B.:** No information  **M:** 1 SR, 1 MA K ≅ C, G-M | **U., B., M.:** No information | ***U:*** 1 SR, 1 MA K > C, G-NA  **B:** No information  **M**: 1 SR, 1 MA K > C, g-NA |
| **Adverse events (Total)** | **U., B., M.:** No information | **U., B., M.:** No information | **U., B., M.:** No information | **U., B., M.:** No information | **U., B., M.:** No information | **U., B., M.:** No information | **U., B., M.:** No information | **U., B., M.:** No information | **U., B., M.:** No information | **U**: 1 SR, 1 MA K ≅ C, G-NA  **B., M.**: No information |
| **Anxiety** | **U., B., M.:** No information | **U., B., M.:** No information | **U., B., M.:** No information | **U., B., M.:** No information | **U., B., M.:** No information | **U., B., M.:** No information | **U., B., M.:** No information | **U**: 1 SR, 1 MA K ≅ C, G-NA  **B., M.**: No information | **U**: 1 SR, 1 MA K ≅ C, G-NA  **B., M.**: No information | **U**: 1 SR, 1 MA K ≅ C, G-H  **B:** No information  **M**: 1 SR, 1 MA K ≅ C, G-NA |
| **Anxiety/Agitation** | **U., B., M.:** No information | **U., B., M.:** No information | **U., B., M.:** No information | **U., B., M.:** No information | **U**: 1 SR, 1 MA K ≅ C, G-NA  **B., M.**: No information | **U., B., M.:** No information | **U:** 1 SR, 1MA  K ≅ C, G-NA, H-1RCT  **B., M.**: No information | **U., B., M.:** No information | **U., B., M.:** No information | **U., B., M.:** No information |
| **Blood pressure increase** | **U., B., M.:** No information | **U., B., M.:** No information | **U., B., M.:** No information | **U., B., M.:** No information | **U., B., M.:** No information | **U., B., M.:** No information | **U., B., M.:** No information | **U., B., M.:** No information | **U., B., M.:** No information | **U**: 1 SR, 1 MA, K ≅ C, G-H ,H-M  **B., M.:** No information |
| **Blurred vision** | **U., B., M.:** No information | **U., B., M.:** No information | **U., B., M.:** No information | **U., B., M.:** No information | **U**: 1 SR, 1 MA K > C, G-NA, H-1RCT  **B., M.**: No information | **U., B., M.:** No information | **U**: 1 SR, 1 MA K ≅ C, G-NA, H-1RCT  **B., M.**: No information | **U**: 1 SR, 1 MA K > C, G-NA, H-NA  **B., M.**: No information | **U**: 1 SR, 1MA K > C, G-NA,  H-1RCT  **B., M.**: No information | **U**: 1 SR, 1 MA K > C, G-NA, H-L  **B., M.**: No information |
| **BPRS** | **U., B.**: No information  **M**: 3 SR, 3MA, 3/3 K > C, 3/3 G-NA,  H-L (in 1 SR), H-M (in 1 SR) | **U., B.**: No information  **M**: 2 SR, 2MA, 2/2 K ≅ C, 2/2 G-NA H-L (in 1 SR) | **U., B.**: No information  **M**: 1 SR, 1 MA K ≅ C, G-NA, H-NA | **U., B.**: No information  **M**: 1 SR, 1 MA, G-NA, K ≅ C, H-NA | **U., B.**: No information  **M**: 1 SR, 1MA K ≅ C, G-NA, H-NA | **U., B.**: No information  **M**: 1 SR, 1MA  K < C, G-NA, H-NA | **U., B.:** No information  **M**: 1 SR, 1 MA, G-NA K ≅ C, H-NA | **B**: 1 SR, 1MA K ≅ C, G-NA, H-NA  **U., M.**: No information | **U., B., M.:** No information | **U., B.:** No information  **M:** 1SR, 1MA K>C, G-NA, H-NA |
| **CADSS** | **U., B.**: No information  **M**:2 SR, 2MA K>C, G-NA, 1/2 H-H, 1/2 H-NA | **B:** 1 SR, 1 MA K ≅ C, G-NA, H-NA  **U., M.**: No information | **B**: 1 SR, 1MA K ≅ C, G-NA, H-NA  **U., M.**: No information | **B**: 1 SR, 1MA K ≅ C, G-NA H-NA  **U., M.**: No information | **B**: 1 SR, 1MA K ≅ C, G-NA, H-NA  **U., M.**: No information | **B**: 1 SR, 1MA K ≅ C, G-NA, H-NA  **U., M.**: No information | **B**: 1 SR, 1MA K ≅ C, G-NA, H-NA  **U., M.**: No information | **B**: 1 SR, 1MA K ≅ C, G-NA, H-NA  **U., M.**: No information | **U., B., M.:** No information | **U., B.**: No information  **M**: 1 SR, 1MA K > C, H-NA, G-NA |
| **Change in blood pressure** | **U., B., M.:** No information | **U., B., M.:** No information | **U., B., M.:** No information | **U., B., M.:** No information | **U., B., M.:** No information | **U., B., M.:** No information | **U., B., M.:** No information | **U., B., M.:** No information | **U., B., M.:** No information | **U**: 1 SR, 1MA K ≅ C,G-NA, H-1RCT  **B., M.**: No information |
| **Chest pain** | **U., B., M.:** No information | **U., B., M.:** No information | **U., B., M.:** No information | **U., B., M.:** No information | **U**: 1 SR, 1MA K ≅ C, G-NA, H-1RCT  **B., M.**: No information | **U., B., M.:** No information | **U**: 1 SR, 1MA K ≅ C, G-NA, H-1RCT  **B., M.**: No information | **U., B., M.:** No information | **U., B., M.:** No information | **U., B., M.:** No information |
| **Confusion** | **U., B., M.:** No information | **U., B., M.:** No information | **U., B., M.:** No information | **U., B., M.:** No information | **U., B., M.:** No information | **U., B., M.:** No information | **U., B., M.:** No information | **U., B., M.:** No information | **U**: 1 SR, 1MA K > C, G-NA, H-1RCT  **B., M.**: No information | **U., B., M.:** No information |
| **Constipation** | **U., B., M.:** No information | **U., B., M.:** No information | **U., B., M.:** No information | **U., B., M.:** No information | **U**: 1 SR, 1MA K ≅ C, G-NA, H-1RCT  **B., M.**: No information | **U., B., M.:** No information | **U**: 1 SR, 1MA K ≅ C, G-NA, H-1RCT  **B., M.**: No information | **U**: 1 SR, 1MA K ≅ C, G-NA, H-NA  **B., M.**: No information | **U., B., M.**: No information | **U., B., M.:** No information |
| **Decreased energy** | **U., B., M.:** No information | **U., B., M.:** No information | **U., B., M.:** No information | **U., B., M.:** No information | **U**: 1 SR, 1MA K ≅ C, G-NA, H-1RCT  **B., M.**: No information | **U., B., M.:** No information | U: 1 SR, 1MA K ≅ C, G-NA, H-1RCT  B., M.: No information | **U., B., M.:** No information | **U., B., M.:** No information | **U., B., M.:** No information |
| **Diarrhea** | **U., B., M.:** No information | **U., B., M.:** No information | **U., B., M.:** No information | **U., B., M.:** No information | **U**: 1 SR, 1MA K ≅ C, G-NA, H-1RCT  **B., M.**: No information | **U., B., M.:** No information | **U:** 1 SR, 1MA K ≅ C, G-NA, H-1RCT  **B., M.**: No information | **U:** 1 SR, 1MA K ≅ C, G-NA, H-NA  **B., M.**: No information | **U., B., M.:** No information | **U**: 1 SR, 1MA K ≅ C, G-H, H-L  **B., M.**: No information |
| **Diplopia** | **U., B., M.:** No information | **U., B., M.:** No information | **U., B., M.:** No information | **U., B., M.:** No information | **U., B., M.:** No information | **U., B., M.:** No information | **U., B., M.:** No information | **U., B., M.:** No information | **U:** 1 SR, 1 MA K > C, G-NA, H-1RCT  **B., M.**: No information | **U., B., M.:** No information |
| **Discontinuation due to intolerability** | **U., B., M.:** No information | **U., B., M.:** No information | **U., B., M.:** No information | **U., B., M.:** No information | **U., B., M.:** No information | **U., B., M.:** No information | **U., B., M.:** No information | **U., B., M.:** No information | **U., B., M.:** No information | **U:** 1 SR, 1MA K > C, G-H, H-L  **B., M.**: No information |
| **Dissociative Symptoms** | **U., B., M.:** No information | **U., B., M.:** No information | **U., B., M.:** No information | **U., B., M.:** No information | **U., B., M.:** No information | **U., B., M.:** No information | **U., B., M.:** No information | **U**: 1 SR, 1 MA K > C G-NA, H-NA  **B., M.**: No information | **U**: 1 SR, 1MA K > C, G-NA, H-1RCT  **B., M.**: No information | **U**: 3 SR, 2/3 K > C, 1/3 K ≅ C, 2/3 G-H, 1/3 G-NA, H-L (in 2 SR),  **B**: No information  **M**: 1 SR, 1MA, K > C, G-NA H-L |
| **Dizziness** | **U., B., M.:** No information | **U., B., M.:** No information | **U., B., M.:** No information | **U., B., M.:** No information | **U**: 1 SR, 1MA K > C,G-NA, H-1RCT  **B., M.**: No information | **U., B., M.:** No information | **U**: 1 SR,1 MA K ≅ C,G-NA, H-1RCT  **B., M.**: No information | **U**: 1 SR, 1 MAK > C, G-NA, H-NA  **B., M.**: No information | **U**: 1 SR, 1MA K > C, G-NA, H-1RCT  **B., M.**: No information | **U**: 2 SR,  2 MAK > C, 1/2 G-H, 1/2 G-NA, H-L  **B**: No information  **M**: 1 SR, 1MA K > C, G-NA, H-L |
| **Dry mouth** | **U., B., M.:** No information | **U., B., M.:** No information | **U., B., M.:** No information | **U., B., M.:** No information | **U**: 1 SR, 1MA K ≅ C, G-NA, H-1RCT  **B., M.**: No information | **U., B., M.:** No information | **U**: 1 SR, 1MA K ≅ C, G-NA, H-1RCT  **B., M.**: No information | **U**: 1 SR, 1MA K ≅ C, G-NA, H-NA  **B., M.**: No information | **U., B., M.:** No information | **U., B., M.**: No information |
| **Dry skin** | **U., B., M.:** No information | **U., B., M.:** No information | **U., B., M.:** No information | **U., B., M.:** No information | **U**: 1 SR, 1MA K ≅ C, G-NA, H-1RCT  **B., M.**: No information | **U., B., M.:** No information | **U:** 1 SR, 1MA K ≅ C, G-NA, H-1RCT  **B., M.**: No information | **U., B., M.:** No information | **U., B., M.:** No information | **U., B., M.:** No information |
| **Dysgeusia** | **U., B., M.:** No information | **U., B., M.:** No information | **U., B., M.:** No information | **U., B., M.:** No information | **U., B., M.**: No information | **U., B., M.:** No information | **U., B., M.:** No information | **U**: 1 SR, 1MA K > C, G-NA, H-NA  **B., M.**: No information | **U**: 1 SR, 1 MA K > C, G-NA, H-1RCT  **B., M.**: No information | **U**: 2 SR, 2 MA K ≅ C, 1/2 G-H, 1/2 G-NA. H-L  **B**: No information  **M**: 1 SR, 1 MA K > C, G-NA, H-M |
| **Emotional blunting** | **U., B., M.:** No information | **U., B., M.:** No information | **U., B., M.:** No information | **U., B., M.:** No information | **U., B., M.**: No information | **U., B., M.:** No information | **U., B., M.:** No information | **U., B., M.**: No information | **U., B., M.:** No information | **U**: 1 SR, 2MA K > C, 1/2 G-L, 1/2 G-NA, H-1RCT  **B., M.**: No information |
| **Euphoria** | **U., B., M.:** No information | **U., B., M.:** No information | **U., B., M.:** No information | **U., B., M.:** No information | **U., B., M.:** No information | **U., B., M.:** No information | **U., B., M.:** No information | **U**: 1 SR, 1MA K ≅ C, G-NA, H-NA  **B., M.**: No information | **U., B., M.:** No information | **U**: 2 SR, 2MA K ≅ C, 1/2 G-H, 1/2 G-NA, H-L (in 1SR)  **B., M.**: No information |
| **Fatigue** | **U., B., M.:** No information | **U., B., M.:** No information | **U., B., M.:** No information | **U., B., M.:** No information | **U**: 1 SR, 1MA K ≅ C, G-NA, H-1RCT  **B., M.**: No information | **U., B., M.:** No information | **U**: 1 SR, 1MA K ≅ C, G-NA, H-1RCT  **B., M.**: No information | **U**: 1 SR, 1MA K ≅ C, G-NA, H-NA  **B., M.**: No information | **U., B., M.:** No information | **U**: 1 SR, 1MA K ≅ C, G-H, H-L  **B., M.**: No information |
| **Feeling abnormal** | **U., B., M.:** No information | **U., B., M.:** No information | **U., B., M.:** No information | **U., B., M.:** No information | **U., B., M.**: No information | **U., B., M.:** No information | **U., B., M.:** No information | **U., B., M.:** No information | **U., B., M.:** No information | **U**: 1 SR, 1 MA K > C, G-H, H-L  **B., M.:** No information |
| **Feeling drunk** | **U., B., M.:** No information | **U., B., M.:** No information | **U., B., M.:** No information | **U., B., M.:** No information | **U., B., M.**: No information | **U., B., M.**: No information | **U., B., M.**: No information | **U**: 1 SR, 1MA K > C, G-NA, H-NA  **B., M.**: No information | **U., B., M.:** No information | **U**: 1 SR, 1MA K > C, G-H, H-L  : No information |
| **General malaise** | **U., B., M.:** No information | **U., B., M.:** No information | **U., B., M.:** No information | **U., B., M.:** No information | **U**: 1 SR, 1MA K < C, G-NA, H-1RCT  **B., M.**: No information | **U., B., M.**: No information | **U**: 1 SR, 1MA K ≅ C, G-NA, H-1RCT  **B., M.**: No information | **U., B., M.:** No information | **U., B., M.:** No information | **U., B., M.:** No information |
| **Headache** | **U., B., M.:** No information | **U., B., M.:** No information | **U., B., M.:** No information | **U., B., M.:** No information | **U:** 1 SR, 1MA K ≅ C,G-NA, H-1RCT  **B., M.:** No information | **U., B., M.:** No information | **U**: 1 SR, 1 MA K ≅ C, G-NA, H-1RCT  **B., M.**: No information | **U**: 1 SR, 1 MA K ≅ C, G-NA, H-NA  **B., M.**: No information | **U**: 1 SR, 1 MA K > C, G-NA, H-1RCT  **B., M.**: No information | **U**: 1 SR, 1 MA K ≅ C,G-NA, H-L  **B**: No information  **M**: 1 SR, 1 MA K > C,G-NA, H-L |
| **Hypoestesia** | **U., B., M.:** No information | **U., B., M.:** No information | **U., B., M.:** No information | **U., B., M.:** No information | **U., B., M.:** No information | **U., B., M.:** No information | **U., B., M.:** No information | **U**: 1 SR, 1MA K > C,G-NA, H-NA  **B., M.**: No information | **U**: 1 SR,1MA K > C,G-NA, H-1RCT  **B., M.**: No information | **U**: 1 SR, 1MA K > C, G-NA,H-L  **B**: No information  **M**: 1 SR,1 MA K > C,G-NA, H-L |
| **Hypoestesia oral** | **U., B., M.:** No information | **U., B., M.:** No information | **U., B., M.:** No information | **U., B., M.:** No information | **U., B., M.:** No information | **U., B., M.:** No information | **U., B., M.:** No information | **U**: 1 SR,1 MA K > C,G-NA, H-NA  **B., M.**: No information | **U**: 1 SR,1MA K > C,G-NA, H-1RCT  **B., M.**: No information | **U**: 1 SR,1 MA K > C,G-NA, H-L  **B., M.**: No information |
| **Increased blood pressure** | **U., B., M.:** No information | **U., B., M.:** No information | **U., B., M.:** No information | **U., B., M.:** No information | **U., B., M.:** No information | **U., B., M.:** No information | **U., B., M.:** No information | **U**: 1 SR,1 MA K > C,G-NA, H-NA  **B., M.**: No information | **U**: 1 SR,1 MA K ≅ C,G-NA, H-1RCT  **B., M**.: No information | **U**: 1 SR, 1MA K ≅ C,G-H, H-L  **B**: No information  **M**: 1 SR,1MA K > C,G-NA, H-L |
| **Increased perspiration** | **U., B., M.:** No information | **U., B., M.:** No information | **U., B., M.:** No information | **U., B., M.:** No information | **U**: 1 SR,1MA K ≅ C,G-NA, H-1RCT  **B., M.**: No information | **U., B., M.:** No information | **U**: 1 SR,1MA K ≅ C,G-NA, H-1RCT  **B., M.**: No information | **U., B., M.:** No information | **U., B., M.:** No information | **U., B., M.:** No information |
| **Insomnia** | **U., B., M.:** No information | **U., B., M.:** No information | **U., B., M.:** No information | **U., B., M.:** No information | **U., B., M.**: No information | **U., B., M.:** No information | **U., B., M.:** No information | **U**: 1 SR,1MA K ≅ C,G-NA, H-NA  **B., M.**: No information | **U., B., M.:** No information | **U**: 1 SR,1 MA K ≅ C,G-H, H-L  **B., M.**: No information |
| **Itching** | **U., B., M.:** No information | **U., B., M.:** No information | **U., B., M.:** No information | **U., B., M.:** No information | **U**: 1 SR,1MA K ≅ C,G-NA, H-1RCT  **B., M.**: No information | **U., B., M.:** No information | **U**: 1 SR,1 MA K ≅ C,G-NA, H-1RCT  **B., M.**: No information | **U., B., M.:** No information | **U., B., M.:** No information | **U., B., M.:** No information |
| **Lethargy** | **U., B., M.:** No information | **U., B., M.:** No information | **U., B., M.:** No information | **U., B., M.:** No information | **U., B., M.:** No information | **U., B., M.:** No information | **U., B., M.:** No information | **U., B., M.:** No information | **U., B., M.:** No information | **U**: 1 SR,1 MA K > C,G-H, H-L  **B., M.**: No information |
| **Mental impairment** | **U., B., M.:** No information | **U., B., M.:** No information | **U., B., M.:** No information | **U., B., M.:** No information | **U., B., M.:** No information | **U., B., M.:** No information | **U., B., M.:** No information | **U., B., M.:** No information | **U., B., M.:** No information | **U**: 1 SR,1MA K ≅ C,G-H, H-L  **B**: No information  **M**: No information |
| **Nasal discomfort** | **U., B., M.:** No information | **U., B., M.:** No information | **U., B., M.:** No information | **U., B., M.:** No information | **U., B., M.:** No information | **U., B., M.:** No information | **U., B., M.:** No information | **U**: 1 SR,1 MA K ≅ C,G-NA, H-NA  **B., M.:** No information | **U**: 1 SR,1 MA K ≅ C,G-NA, H-1RCT  **B., M.**: No information | **U:** 1 SR,1 MA K ≅ C,G-H, H-L  **B., M.**: No information |
| **Nausea/Vomiting** | **U., B., M.:** No information | **U., B., M.:** No information | **U., B., M.:** No information | **U., B., M.:** No information | **U**:1 SR, 1 MA K=C, G-NA  **B**: No information  **M**: No information | **U., B., M.:** No information | **U**: 1 SR, 1 MA K=C, G-NA  **B**: No information  **M**: No information | **U**: No information  **B**: No information  **M**: No information | **U**: No information  **B**: No information  **M**: No information | **U**: No information  **B**: No information  **M**: No information |
| **Neurological symptoms** | **U., B., M.:** No information | **U., B., M.:** No information | **U., B., M.:** No information | **U., B., M.:** No information | **U., B., M.:** No information | **U., B., M.:** No information | **U., B., M.:** No information | **U., B., M.:** No information | **U., B., M.:** No information | **U**: 1 SR,1 MA K ≅ C,G-NA, H-L  **B., M.**: No information |
| **Oral paresthesia** | **U., B., M.:** No information | **U., B., M.:** No information | **U., B., M.:** No information | **U., B., M.:** No information | **U., B., M.:** No information | **U., B., M.:** No information | **U., B., M.:** No information | **U**: 1 SR,1 MA K > C,G-NA, H-NA  **B., M.**: No information | **U**: 1 SR, 1 MA K ≅ C,G-NA, H-1RCT  **B., M.**: No information | **U**: 1 SR,1 MA K ≅ C,G-H, H-M  **B., M.**: No information |
| **Oropharyngeal pain** | **U., B., M.:** No information | **U., B., M.:** No information | **U., B., M.:** No information | **U., B., M.:** No information | **U., B., M.:** No information | **U., B., M.:** No information | **U., B., M.:** No information | **U**: 1 SR,1 MA K ≅ C,G-NA, H-NA  **B., M.**: No information | **U., B., M.:** No information | **U**: 1 SR,1 MA K ≅ C,G-H H-L  **B., M.**: No information |
| **Palpitations** | **U., B., M.:** No information | **U., B., M.:** No information | **U., B., M.:** No information | **U., B., M.:** No information | **U**: 1 SR,1 MA K ≅ C,G-NA, H-1RCT  **B., M.**: No information | **U., B., M.:** No information | **U**: 1 SR,1 MA K ≅ C, G-NA, H-1RCT  **B., M.**: No information | **U., B., M.:** No information | **U., B., M.:** No information | **U., B., M.**: No information |
| **Paresthesia** | **U., B., M.:** No information | **U., B., M.:** No information | **U., B., M.:** No information | **U., B., M.:** No information | **U., B., M.:** No information | **U., B., M.:** No information | **U., B., M.:** No information | **U**: 1 SR, 1 MA K > C,G-NA, H-NA  **B., M.**: No information | **U**: 1 SR,1 MA K > C,G-NA, H-1RCT  **B., M.**: No information | **U:** 1 SR,1 MA K > C,G-H, H-L  **B:** No information  **M**: 1 SR,1 MA K > C,G-NA, H-L |
| **Pollakiuria** | **U., B., M.:** No information | **U., B., M.:** No information | **U., B., M.:** No information | **U., B., M.:** No information | **U., B., M.:** No information | **U., B., M.:** No information | **U., B., M.**: No information | **U., B., M.:** No information | **U., B., M.:** No information | **U**: 1 SR,1 MA K ≅ C,G-H, H-L  **B., M.**: No information |
| **Poor co-ordination** | **U., B., M.:** No information | **U., B., M.:** No information | **U., B., M.:** No information | **U., B., M.:** No information | **U., B., M.:** No information | **U., B., M.:** No information | **U**: 1 SR,1 MA K ≅ C,G-NA, H-1RCT  **B., M.**: No information | **U., B., M.:** No information | **U., B., M.:** No information | **U., B., M.**: No information |
| **Poor con-centration** | **U., B., M.:** No information | **U., B., M.:** No information | **U., B., M.:** No information | **U., B., M.:** No information | **U**: 1 SR,1 MA K ≅ C,G-NA, H-1RCT  **B., M.**: No information | **U., B., M**.: No information | ***U***: 1 SR,1 MA K ≅ C,G-NA, H-1RCT  **B., M.**: No information | **U., B., M.:** No information | **U., B., M.:** No information | **U., B., M.:** No information |
| **Postural dizziness** | **U., B., M.:** No information | **U., B., M.:** No information | **U., B., M.:** No information | **U., B., M.:** No information | **U., B., M.:** No information | **U., B., M.:** No information | **U., B., M.:** No information | **U**: 1 SR, 1 MA K > C,G-NA, H-1RCT  **B., M.**: No information | **U**: 1 SR, 1 MA K ≅ C,G-NA, H-1RCT  **B., M.:** No information | **U**: 1 SR,1 MA K > C,G-H, H-L  **B., M.**: No information |
| **Psychiatric symptoms** | **U., B., M.:** No information | **U., B., M.:** No information | **U., B., M.:** No information | **U., B., M.:** No information | **U., B., M.:** No information | **U., B., M.:** No information | **U., B., M.:** No information | **U., B., M.:** No information | **U., B., M.:** No information | **U**: 1 SR,1 MA K ≅ C,G-NA, H-L  **B., M.**: No information |
| **Rash** | **U., B., M.:** No information | **U., B., M.:** No information | **U., B., M.:** No information | **U., B., M.:** No information | **U**: 1 SR,1 MA K ≅ C,G-NA, H-1RCT  **B., M.**: No information | **U., B., M.:** No information | **U**: 1 SR,1 MA K ≅ C,G-NA, H-1RCT  **B., M.**: No information | **U., B., M.:** No information | **U., B., M.:** No information | **U., B., M.:** No information |
| **Sedation** | **U., B., M.:** No information | **U., B., M.:** No information | **U., B., M.:** No information | **U., B., M.:** No information | **U., B., M.:** No information | **U., B., M.:** No information | **U., B., M.:** No information | **U**: 1 SR,1 MA K > C,G-NA, H-NA  **B., M.**: No information | **U**: 1 SR,1 MA K > C,G-NA, H-1RCT  **B., M.**: No information | **U**: 1 SR,1 MA K > C,G-H, H-L  **B., M.**: No information |
| **Somnolence** | **U., B., M.:** No information | **U., B., M.:** No information | **U., B., M.:** No information | **U., B., M.:** No information | **U., B., M.:** No information | **U., B., M.:** No information | **U., B., M.:** No information | **U**: 1 SR,1 MA K > C,G-NA, H-NA  **B., M.**: No information | **U**: 1 SR,1 MA K > C,G-NA, H-1RCT  **B., M.**: No information | **U**: 1 SR,1 MA K > C,G-H H-L  **B**: No information  **M**: 1 SR,1 MA K > C,G-NA, H-M |
| **Throat irritation** | **U., B., M.:** No information | **U., B., M.:** No information | **U., B., M.:** No information | **U., B., M.:** No information | **U., B., M.:** No information | **U., B., M.:** No information | **U., B., M.:** No information | **U**: 1 SR,1 MA K > C,G-NA, H-NA  **B., M.**: No information | **U**: 1 SR,1 MA K ≅ C,G-NA, H-1RCT  **B., M.:** No information | **U**: 1 SR,1 MA K > C,G-H, H-L  **B., M.**: No information |
| **Tinnitus** | **U., B., M.:** No information | **U., B., M.:** No information | **U., B., M.:** No information | **U., B., M.:** No information | **U**: 1 SR,1 MA K ≅ C,G-NA, H-1RCT  **B., M.**: No information | **U., B., M.:** No information | **U**: 1 SR,1 MA K ≅ C,G-NA, H-1RCT  **B., M**.: No information | **U., B., M.:** No information | **U., B., M.:** No information | **U., B., M.:** No information |
| **Tremor** | **U., B., M.:** No information | **U., B., M.:** No information | **U., B., M.:** No information | **U., B., M.:** No information | **U**: 1 SR,1 MA K ≅ C,G-NA, H-1RCT  **B., M.**: No information | **U., B., M.:** No information | **U**: 1 SR,1 MA K ≅ C,G-NA, H-1RCT  **B., M.**: No information | **U., B., M.:** No information | **U., B., M.:** No information | **U**: 1 SR,1 MA K ≅ C,G-H, H-L  **B., M.**: No information |
| **Urination issues** | **U., B., M.:** No information | **U., B., M.:** No information | **U., B., M.:** No information | **U., B., M.:** No information | **U**: 1 SR, 1 MA K ≅ C, H-1RCT  **B., M.**: No information | **U., B., M.:** No information | **U**: 1 SR,1 MA K ≅ C,G-NA, H-1RCT  **B., M.**: No information | **U., B., M.:** No information | **U., B., M.:** No information | **U., B., M.:** No information |
| **Vertigo** | **U., B., M.:** No information | **U., B., M.:** No information | **U., B., M.:** No information | **U., B., M.:** No information | **U., B., M.:** No information | **U., B., M.:** No information | **U., B., M.:** No information | **U**: 1 SR, 1 MA K > C, G-NA, H-NA  **B., M.**: No information | **U**: 1 SR, 1 MA K > C, G-NA, H-1RCT  **B., M.**: No information | **U**: 2 SR, 2 MA K > C, 1/2 G-H, 1/2 G-NA, H-L  **B**: No information  **M**: 1 SR,1 MA K > C,G-NA, H-L |
| **Viral upper respiratory infection** | **U., B., M.:** No information | **U., B., M.:** No information | **U., B., M.:** No information | **U., B., M.:** No information | **U., B., M.:** No information | **U., B., M.:** No information | **U., B., M.:** No information | **U., B., M.:** No information | U: 1 SR, 1 MA K ≅ C,G-NA, H-NA  B., M.: No information | **U., B., M.:** No information |
| **Nausea** | **U., B., M.:** No information | **U., B., M.:** No information | **U., B., M.:** No information | **U., B., M.:** No information | **U., B., M.:** No information | **U., B., M.:** No information | **U., B., M.:** No information | **U**: 1 SR, 1 MA K>C, G-NA;  **B**: No information  **M**: No information | **U**: 1 SR, 1 MA K>C, G-NA  **B**: No information  **M**: No information | **U**: 2 SR, 2 MA K>C, 1/2 G-H; 1/2 G-NA  **B**: No information  **M**: 1 SR, 1 MA K>C, G-NA |
| **Vomiting** | **U., B., M.:** No information | **U., B., M.:** No information | **U., B., M.:** No information | **U., B., M.:** No information | **U., B., M.:** No information | **U., B., M.:** No information | **U., B., M.:** No information | **U**: 1 SR, 1 MA K>C, G-NA  **B**: No information  **M**: No information | **U**: 1 SR, 1 MA K>C, G-NA  **B**: No information  **M**: No information | **U**: 2 SR, 2 MA K>C, 1/2 G-H, 1/2 G-NA  **B**: No information  **M**: No information |

## Supplementary Tables 9. Citation matrices

AE-CONF = Adverse Avent - Confusion

AE-DISS = Adverse Avent – Dissociative Symptoms

AE-GASTRO =Adverse Avent - Gastrointestinal

AE-HALL = Adverse Avent - Hallucinations

AE-NEURO = Adverse Avent - Neurological

AE-OVER = Adverse Avent - Overall

AE-PSY = Adverse Avent - Psychiatric

BPRS = Brief Psychiatric Rating Scale

CADSS = Clinician-Administered Dissociative States Scale

DR = Dropout Rate

DS = Depression Score

LOE = Lack of Efficacy

REM = Remission

RES = Response

SI = Suicide Ideation

TD = Total Dropouts

*Citation matrix 1*

Population (by eligibility):

- Major Depressive Disorder or Bipolar Disorder
- Resistant: Not specified
- Suicidality: Not specified

Intervention (by eligibility): Ketamine

| **ID** | **Kishimoto 2016** | **Nuñez 2020*** | **Rhee 2022**** |
| --- | --- | --- | --- |
| **Arabzadeh 2018** |  | X,REM, DS, RES, AE-OVER, AE-NEURO, AE-PSY |  |
| **Basso 2020** |  |  | X |
| **Berman 2000** | X,C, DS, TD, RES, BPRS |  |  |
| **Diazgranados 2010** | X,REM, DS, TD, RES, BPRS, CADSS |  |  |
| **Domany 2018** |  | X,REM, DS, RES |  |
| **Ekstrand 2022** |  |  | X |
| **Ghasemi 2014** |  |  | X,DS |
| **Jafarinia 2016** |  | X,REM, DS, RES, AE-OVER, AE-NEURO, AE-PSY |  |
| **Kheirabadi 2019** |  |  | X,DS |
| **Kheirabadi 2020** |  |  | X,DS |
| **Lai 2014** | X,REM, DS, RES, |  |  |
| **Murrough 2013** | X,DS, TD, RES, BPRS, CADSS |  |  |
| **Sharma 2020** |  |  | X,DS |
| **Singh 2016** | X,DS |  |  |
| **Sos 2013** | X,DS, TD, RES |  |  |
| **Zarate 2006** | X,REM, DS, TD, RES, BPRS |  |  |
| **Zarate 2012** | X,REM, DS, TD, RES, BPRS, CADSS |  |  |

* Only on oral ketamine as intervention

** Only ketamine vs electroconvulsive therapy

*Citation matrix 2*

Population (by eligibility):

- Major Depressive Disorder or Bipolar Disorder
- Resistant: Not specified
- Suicidality: Not specified

Intervention (by eligibility): Ketamine and Esketamine

| **ID** | **Bahji 2022** | **Caddy 2015** | **Fond 2014** | **Han 2016** | **Hock 2022** | **McGirr 2015** | **McIntyre 2020** | **Newport 2015** | **Romeo 2015** |
| --- | --- | --- | --- | --- | --- | --- | --- | --- | --- |
| **Arabzadeh 2018** | X |  |  |  |  |  | X,DS |  |  |
| **Berman 2000** | X | X,REM, DS, RES | X,DS | X,REM, RES |  | X,DS, TD, RES, BPRS | X | X,REM, RES, BPRS | X,DS, BPRS |
| **Canuso 2018** | X |  |  |  | X,DS |  | X,DS |  |  |
| **Cao 2019** | X |  |  |  |  |  |  |  |  |
| **Chen 2019** | X |  |  |  |  |  | X |  |  |
| **Correia-Melo 2020** | X |  |  |  |  |  |  |  |  |
| **Daly 2018** | X |  |  |  | X,DS |  | X,DS |  |  |
| **Diazgranados 2010** | X |  | X,DS |  |  | X,REM, DS, TD, RES, BPRS, CADSS | X | X,REM, RES, BPRS, CADSS | X,DS, BPRS |
| **Domany 2018** | X |  |  |  |  |  | X,DS |  |  |
| **Downey 2016** | X |  |  |  |  |  |  |  |  |
| **Fan 2017** |  |  |  |  |  |  | X |  |  |
| **Fava 2018** | X |  |  |  |  |  | X |  |  |
| **Fedgchin 2019** | X |  |  |  | X,DS |  | X,DS |  |  |
| **Fu 2020** | X |  |  |  | X,DS |  |  |  |  |
| **Galvez 2018** | X |  |  |  |  |  |  |  |  |
| **George 2020** | X |  |  |  |  |  |  |  |  |
| **Grunebaum 2017** | X |  |  |  |  |  |  |  |  |
| **Grunebaum 2018** | X |  |  |  |  |  |  |  |  |
| **Ghasemi 2014** |  | X,REM, DS, RES | X,DS | X,REM, RES |  |  |  |  |  |
| **Jarventausta 2013** |  | X |  |  |  |  |  |  |  |
| **Kudoh 2002** |  |  | X |  |  |  | X |  |  |
| **Hu 2016** | X |  |  | X,REM, RES |  |  |  |  |  |
| **Ibrahim 2012** |  |  |  |  |  |  | X |  |  |
| **Ionescu 2019** | X |  |  |  | X,DS |  |  |  |  |
| **Lai 2014** | X |  |  |  |  |  |  |  |  |
| **Jafarinia 2016** |  |  |  |  |  |  | X,DS |  |  |
| **Lapidus 2014** | X | X |  | X,RES |  | X,DS, TD, RES, BPRS, CADSS | X,DS | X,REM, RES | X,DS, BPRS |
| **Li 2016** | X |  |  |  |  |  |  |  |  |
| **Loo 2016** | X | X |  |  |  |  |  |  |  |
| **Murrough 2013** | X | X,SS, REM, DS, TD, RES | X,DS | X,REM, RES |  | X,REM, DS, TD, RES, BPRS, CADSS | X | X,REM, RES, BPRS, CADSS |  |
| **Murrough 2015** | X |  |  |  |  |  | X |  |  |
| **NCT2918318** |  |  |  |  | X,DS |  |  |  |  |
| **Nugent 2019** | X |  |  |  |  |  |  |  |  |
| **Och-Ross 2019** | X |  |  |  | X,DS |  |  |  |  |
| **Phillips 2019** | X |  |  |  |  |  | X |  |  |
| **Popova 2019** | X |  |  |  | X,DS |  |  |  |  |
| **Singh 2016** | X |  |  | X |  |  | X |  |  |
| **Sos 2013** | X | X,REM, DS, RES, AE-DISS | X,DS | X,REM, RES |  | X,REM, DS, TD, RES, BPRS | X | X,REM, RES | X,DS, BPRS |
| **Su 2017** | X |  |  |  |  |  | X |  |  |
| **Yoosefi 2014** |  | X |  |  |  |  |  |  |  |
| **Zarate 2006** | X | X,REM, DS, RES | X,DS | X,REM, RES |  | X,REM, DS, TD, RES, BPRS | X | X,REM, RES, BPRS | X,DS, BPRS |
| **Zarate 2012** | X |  | X,DS |  |  | X,REM, DS, TD, RES, BPRS, CADSS |  | X,REM, RES, BPRS, CADSS | X,DS, BPRS |

*Citation matrix 3*

Population (by eligibility):

- Major Depressive Disorder or Bipolar Disorder
- Resistant: Yes
- Suicidality: Not specified

Intervention (by eligibility): Ketamine

| **ID** | **Lee 2015** | **Marcantoni 2020** |
| --- | --- | --- |
| **Berman 2000** | X | X |
| **Cao 2019** |  | X |
| **Chen 2018** |  | X,DS, RES |
| **Diazgranados 2010** | X,DS | X,REM, DS, RES |
| **Fava 2018** |  | X,DS, RES |
| **Ghasemi 2014** |  | X |
| **Grunebaum 2018** |  | X,DS, RES |
| **Murrough 2013** | X,DS | X,REM, DS, RES |
| **Murrough 2015** |  | X |
| **Singh 2016** |  | X |
| **Sos 2013** | X,DS | X,REM, DS, RES |
| **Zarate 2006** | X,DS | X,REM, DS, RES |
| **Zarate 2012** | X,DS |  |

*Citation matrix 4*

Population (by eligibility):

- Bipolar Disorder
- Resistant: Not specified
- Suicidality: Not specified

Intervention (by eligibility): Ketamine

| **ID** | **Bahji 2020** | **Dean 2021 (McCloud 2015 Update Handsearched)** |
| --- | --- | --- |
| **Diazgranados 2010** | X,REM, DS, RES | X,REM, DS, LOE, TD, RES |
| **Grunebaum 2017** |  | X,SS, REM, DS, TD, RES |
| **Zarate 2012** | X,REM, DS, RES | X,REM, DS, LOE, TD, RES |

*Citation matrix 5*

Population (by eligibility):

- Bipolar Disorder
- Resistant: Yes
- Suicidality: Not specified

Intervention (by eligibility): Ketamine, Esketamine

| **ID** | **Fornaro 2020** |
| --- | --- |
| **Diazgranados 2010** | X,DS, TD, RES |
| **Zarate 2012** | X,DS, TD, RES |

*Citation matrix 6*

Population (by eligibility):

- Major Depressive Disorder
- Resistant: Not specified
- Suicidality: Not specified

Intervention (by eligibility): Esketamine

| **ID** | **Jawad  2022** | **Papakostas 2020** | **Zheng_2020** |
| --- | --- | --- | --- |
| **Canuso 2018** | X,REM, DS, RES, AE-DISS | X,REM, DS, RES | X,REM, TD, AE-DISS |
| **Daly 2018** | X,AE-DISS, AE-CONF, AE – HYP | X,REM, DS, RES | X,REM, TD, RES, AE-DISS |
| **Fedgchin 2019** | X,REM, DS, RES, AE-DISS | X,REM, DS, RES | X,REM, TD, RES, AE-DISS |
| **Fu 2020** | X,REM, DS, AE-DISS |  |  |
| **Ionescu 2019** |  |  |  |
| **Ionescu 2021** | X,REM, DS, RES |  |  |
| **Ochs-Ross 2019** | X,REM, DS, RES, AE-DISS | X,REM, DS, RES |  |
| **Popova 2019** | X,REM, DS, RES, AE-DISS | X,REM, DS, RES | X,REM, TD, RES, AE-DISS |
| **Takahashi 2021** | X,REM, DS, RES, AE-DISS |  |  |

*Citation matrix 7*

Population (by eligibility):

- Major Depressive Disorder
- Resistant: Not specified
- Suicidality: Not specified

Intervention (by eligibility): Ketamine, Esketamine

| **ID** | **An 2021** | **Kryst 2020** | **Xu 2016** |
| --- | --- | --- | --- |
| **Arabzadeh 2018** |  | X |  |
| **Berman 2000** |  | X,REM, RES | X |
| **Burger 2016** |  |  |  |
| **Canuso 2018** | X,REM, DS |  |  |
| **Cao 2018** |  | X,RES |  |
| **Chen 2018** |  | X,RES |  |
| **Daly 2018** | X,REM, DS, RES |  |  |
| **Diazgranados 2010** |  | X,REM, RES | X,SS, REM |
| **Domany 2019** |  | X |  |
| **Downey 2016** |  | X |  |
| **Fava 2018** |  | X,RES |  |
| **Fu 2020** |  |  |  |
| **Fedgchin 2019** | X,DS, RES |  |  |
| **Grunebaum 2018** |  | X,RES |  |
| **Hu 2016** |  | X,REM, RES |  |
| **Ionescu 2019** |  | X |  |
| **Lai 2014** |  |  | X,SS |
| **Lapidus 2014** | X,RES | X,RES | X,SS, REM |
| **Murrough 2013** |  | X,RES | X, REM |
| **Murrough 2015** |  | X |  |
| **Nugent 2019** |  | X |  |
| **Ochs-Ross 2019** | X,REM, DS, RES |  |  |
| **Phillips 2019** |  |  |  |
| **Popova 2019** | X,REM, DS, RES |  |  |
| **Price 2014** |  |  |  |
| **Singh 2016** |  | X |  |
| **Sos 2013** |  | X,REM, RES | X,SS, REM |
| **Su 2017** |  | X,RES |  |
| **Sinyor 2018** |  |  |  |
| **Zarate 2006** |  | X,REM, RES | X,SS, REM |
| **Zarate 2012** |  | X,REM, RES | X,SS, REM |

*Citation matrix 8*

Population (by eligibility):

- Major Depressive Disorder
- Resistant: Yes
- Suicidality: Not specified

Intervention (by eligibility): Ketamine, Esketamine

| **ID** | **Papadimitropoulou 2017** |
| --- | --- |
| **Singh 2016** | X,DS, RES |

*Citation matrix 9*

Population (by eligibility):

- Major Depressive Disorder
- Resistant: Not specified
- Suicidality: Not specified

Intervention (by eligibility): Ketamine, Esketamine

| **ID** | **Witt 2020** |
| --- | --- |
| **Anderson 2017** | X |
| **Canuso 2018** | X |
| **George 2017** | X |
| **Grunebaum 2017** | X,SS |
| **Grunebaum 2018** | X,SS |
| **Kudoh 2002** | X |
| **Hu 2016** | X,SS |
| **Loo 2016** | X |
| **Murrough 2013** | X |
| **Murrough 2015** | X |
| **Ray-Griffith 2017** | X |
| **Sos 2013** | X |
| **Su 2017** | X,SS |
| **Zarate 2012** | X |

*Citation matrix 10*

Population (by eligibility):

- Major Depressive Disorder or Bipolar Disorder
- Resistant: Not specified
- Suicidality: yes

Intervention (by eligibility): Ketamine and esketamine

| **ID** | **Xiong 2021** |
| --- | --- |
| **Burger 2016** | X |
| **Canuso 2018** | X,SS |
| **Chen 2019** | X |
| **Domany 2019** | X |
| **Fu 2020** | X |
| **Grunebaum 2017** | X,SS |
| **Grunebaum 2018** | X,SS |
| **Hu 2016** | X,SS |
| **Ionescu 2019** | X,SS |
| **Murrough 2015** | X,SS |
| **Phillips 2019** | X,SS |
| **Price 2014** | X,SS |
| **Sinyor 2018** | X,SS |
| **Zarate 2012** | X |

## Supplementary 10. Deviation from protocol

- We originally planned to limit the studies by outcomes investigated, with reference to the following: suicide risk, depressive symptoms, response to treatment, remission, relapse rate, dropout rate, side eﬀects, and dissociation. Finally, we included all studies regardless of the outcomes investigated. We decided to operate this way so as not to generate selection bias.
- We had originally planned to include only systematic reviews on patients with unipolar and bipolar depression. Finally, we also included studies focusing on suicidal ideation. The rationale for this choice was because almost all of these studies were based on original studies actually on patients with unipolar and bipolar depression.
- In view of the relatively dated protocol registration, we decided to update the search from March 2021 to December 2022.
- Due to the inability to access Embase for logistical reasons, we decided to replace the previously indicated database with Scopus.
- In accordance with the PRIOR guideline for writing Overview of Reviews, we have added Epistemonikos as a search engine for systematic reviews.
- Compared with the original protocol choice of considering only other substances as comparators, we also included studies in which somatotherapies such as electroconvulsive therapy were considered as comparators. Thus, it was decided to have a more comprehensive view of the literature by involving another treatment with the same indication as ketamine.
- The original choice to focus mainly on suicidal ideation was set aside because it would have made the presentation of literature data less all-encompassing.
- Extracting the Risk of Bias of the original studies was not originally planned. However, we added it to provide researchers with more useful data for planning future studies.
